# Reward-Guided Learning with and without Causal Attribution

**DOI:** 10.1016/j.neuron.2016.02.018

**Published:** 2016-04-06

**Authors:** Gerhard Jocham, Kay H. Brodersen, Alexandra O. Constantinescu, Martin C. Kahn, Angela M. Ianni, Mark E. Walton, Matthew F.S. Rushworth, Timothy E.J. Behrens

**Affiliations:** 1 Oxford Centre for Functional MRI of the Brain, Nuffield Department of Clinical Neurosciences, University of Oxford, John Radcliffe Hospital, Oxford OX3 9DU, UK; 2 Center for Behavioral Brain Sciences, Otto-von-Guericke-Universität Magdeburg, Universitätsplatz 2, 39106 Magdeburg, Germany; 3 Faculty of Economics and Management, Otto-von-Guericke-Universität Magdeburg, Universitätsplatz 2, 39106 Magdeburg, Germany; 4 Section on Integrative Neuroimaging, Clinical and Translational Neuroscience Branch, National Institute of Mental Health, National Institutes of Health, Intramural Research Program, Department of Health and Human Services, Bethesda, MD 20892, USA; 5 Department of Experimental Psychology, University of Oxford, South Parks Road, Oxford OX1 3UD, UK; 6 Wellcome Trust Centre for Neuroimaging, University College London, 12 Queen Square, London WC1N 3BG, UK

## Abstract

When an organism receives a reward, it is crucial to know which of many candidate actions caused this reward. However, recent work suggests that learning is possible even when this most fundamental assumption is not met. We used novel reward-guided learning paradigms in two fMRI studies to show that humans deploy separable learning mechanisms that operate in parallel. While behavior was dominated by precise contingent learning, it also revealed hallmarks of noncontingent learning strategies. These learning mechanisms were separable behaviorally and neurally. Lateral orbitofrontal cortex supported contingent learning and reflected contingencies between outcomes and their causal choices. Amygdala responses around reward times related to statistical patterns of learning. Time-based heuristic mechanisms were related to activity in sensorimotor corticostriatal circuitry. Our data point to the existence of several learning mechanisms in the human brain, of which only one relies on applying known rules about the causal structure of the task.

## Highlights

• Reward-guided learning is driven by several mechanisms operating in parallel
• These mechanisms either respect or only approximate the causal structure of the world
• Learning mechanisms respecting causal structure rely on orbitofrontal cortex
• Other regions mediate learning based on temporal proximity and statistical regularity

## Teaser

Jocham et al. demonstrate that learning is driven by several mechanisms operating in parallel, of which only one relies on knowing the relationship between outcomes and choices that cause them. This contingent learning is mediated by activity in orbitofrontal cortex.

## Introduction

An organism’s ability to learn from behavioral outcomes is central to its evolutionary success. Recent decades have seen important advances in our understanding of the computations underlying many flavors of such reinforcement learning, but these models begin with a fundamental assumption, that organisms can attribute each outcome to the behavior that caused it, that is, they can assign the credit for an outcome correctly. Results of recent lesions studies have challenged this assumption, suggesting that learning is possible even when this simplest assumption is not met, and that these noncontingent mechanisms dominate behavior when lesions are made to the lateral orbitofrontal cortex (lOFC; Walton et al., 2010).

On initial consideration, several features of these results are surprising. When learning from rewards, the brain faces many complex computational problems. However, since typical neuroscience experiments separate behavior into discrete trials, there is no ambiguity about action-reward pairings and hence no apparent computational problem to solve. It appears paradoxical, then, that a brain region as evolutionarily recent as lOFC is required for this apparently trivial attribution. It is perhaps equally surprising, however, that any learning is possible in its absence. If an agent does not know which action led to which reward, how can it learn which actions are good at all?

Insights into these seeming conundrums can perhaps be gleaned by considering real-world ecological problems that exist outside the laboratory. In the real world, agents take many actions, and only some of them have consequences. These consequences may be delayed in time with many intervening irrelevant actions. Furthermore, many important outcomes are not even consequences of the agent’s behavior. It becomes a difficult and important problem to discern which outcomes should cause learning, and on which actions (Sutton and Barto, 1998). In reinforcement learning terms, it becomes important to apply the correct state space during learning (Wilson et al., 2014).

One can think of different classes of mechanism for solving this problem. In precise contingent mechanisms, agents may be able to attribute particular outcomes to their causal actions due to external knowledge—if a cake is burned, it is more likely to be caused by the cooking time than the quantity of sugar in the recipe. Similarly, if experimental animals have extensive prior experience of outcomes following actions in a trial structure, they may learn to solve the attribution problem precisely even with a new set of experimental stimuli or type of reward.

In the absence of such external knowledge, it may still be possible to attribute outcomes to actions precisely by using heuristic mechanisms that capitalize on common features of causal relationships. For example, outcomes may be attributed to actions that immediately preceded them—a button press immediately followed by a loud explosion is unlikely to be repeated, but even a few seconds delay may prevent any such association being made. Here, agents can use a heuristic rule that is often true in real-world learning and has therefore been favored by evolution.

Even when attributions cannot be made precisely, they may still be made through statistical mechanisms. If one action has been taken more often than another, or has been pursued for a longer recent period of time, then it is more likely to be the cause of outcomes. Such considerations may lead to learning strategies familiar in ecological theories of behavior (Charnov, 1976) that state that if the time-average reward is high, then agents should continue with current behavioral policies.

Here, we show that in complex environments, healthy humans’ behavior is guided by multiple learning mechanisms operating in parallel. While behavior was dominated by learning on the basis of precise contingent associations between outcomes and their causal choices, behavior also displayed hallmarks of simpler learning mechanisms that do not rely on such contingent associations. We found signals pertaining to the different learning mechanisms in separable brain circuits. Precise contingent learning was supported by a system centered on lOFC. Amygdala activity, or the absence of amygdala suppression, was related principally to statistical learning mechanisms. Proximal heuristic mechanisms were related to circuitry in motor regions of the cortico-striatal circuitry.

## Results

**Figure 1:**
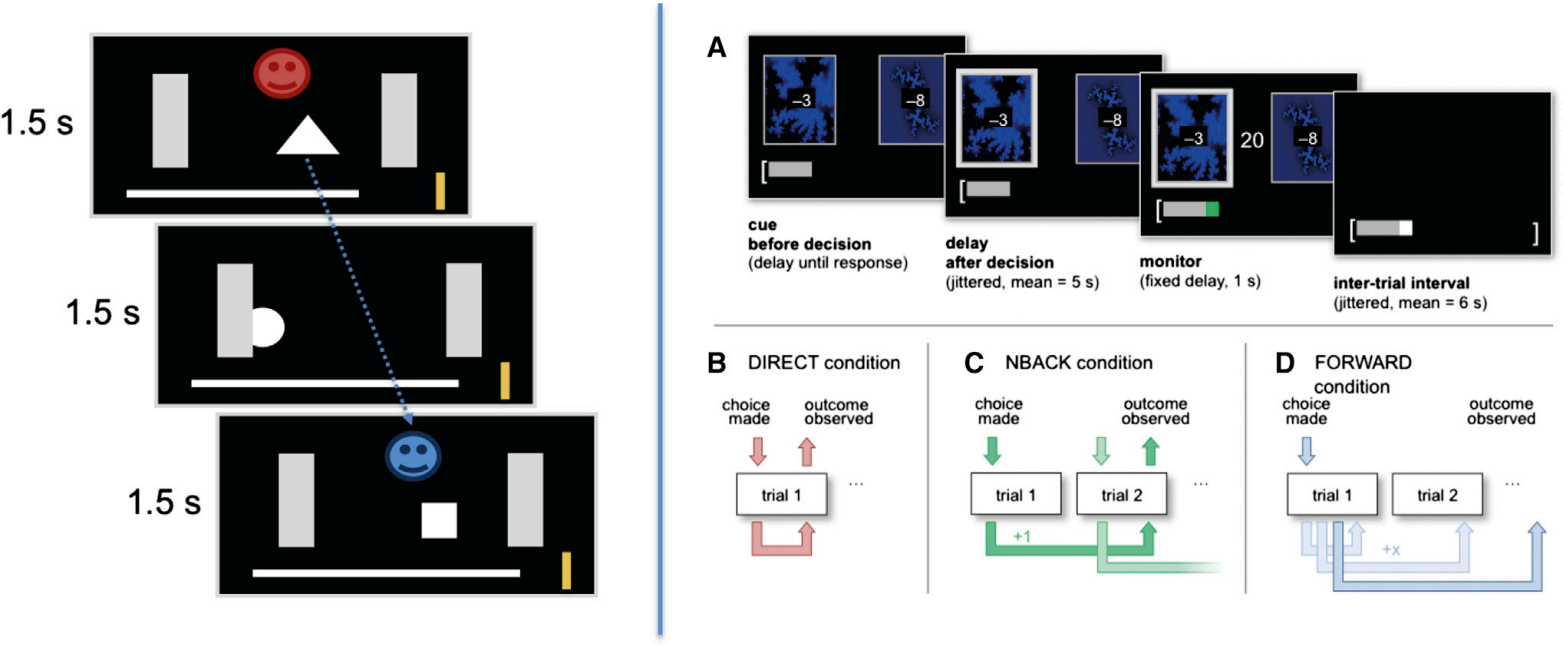
Task Schematic Task schematic for experiment 1 (left) and experiment 2 (right).

We performed two experiments to probe different mechanisms for credit assignment in the intact human brain using fMRI (Figure 1). Experiment 1 was designed to reveal signatures of each learning mechanism in normal behavior and harness fluctuations across the population to investigate their neural bases. Experiment 2 introduced manipulations that interfered with contingencies, allowing us to search for brain signals aligned with contingency, rather than reward or behavior. In both experiments, participants chose between different stimuli with independent probabilities to give rewards. These probabilities changed over time. Thus, subjects needed to continuously learn the stimulus-reward associations. To ensure that participants chose only the stimuli that were likely to give a reward, each choice incurred a cost.

In experiment 1, we aimed to simulate an ecologically realistic situation where many possible choices could be credited for a reward and only some rewards were caused by participant’s behavior. We reasoned that credit assignment by precise contingent learning would be heavily taxed in such an environment, allowing the contribution of other mechanisms to become evident. A total of 23 participants (12 female) were presented with a continuous random succession of geometrical shapes (A / B / C). Shapes were moving across the screen from left to right, one at a time, during a period of 1.5 s (Figure 1, left). While on screen, these options could either be selected by pressing a specific button (incurring a small cost) or ignored. Critically, for a rewarded choice, participants received a contingent reward 3 s after the choice that caused it (subjects were informed and extensively pretrained on this delay). Thus, among the many choices participants made, they had to assign credit only to the specific choices made 3 s prior to reward delivery. However, in addition, subjects also received noncontingent rewards in a random fashion, independent of their behavior. Crucially, these two types of rewards were distinguishable (by color, red or blue, counterbalanced across subjects), and subjects were instructed to focus on contingent rewards and to ignore the noncontingent rewards. Thus, because subjects have to link contingent rewards to the choice made 3 s before rather than to the option currently observed, this design breaks the common “trial-like” structure for reward-guided learning tasks. This design allowed us to quantify interindividual differences in learning from contingent and noncontingent rewards. The rate of noncontingent reward delivery was established during piloting to match that of contingent rewards across subjects.

### Behavior Is Guided by Separable Contingent and Noncontingent Learning Mechanisms

To separate contingent from noncontingent learning in experiment 1, we used a multiple logistic regression to test how rewards following the choice of an option influenced the probability of choosing this same option the next time it was encountered, depending on when this reward occurred relative to the choice. Given that subjects were precisely instructed that rewards were given with a 3 s delay, in a subject relying exclusively on contingent learning, only those rewards occurring around 3 s after a choice should have an impact on reselecting that same stimulus. If, in contrast, subjects relied on noncontingent learning, then credit for rewards should spread back to noncausal choices made in the recent past.

**Figure 2:**
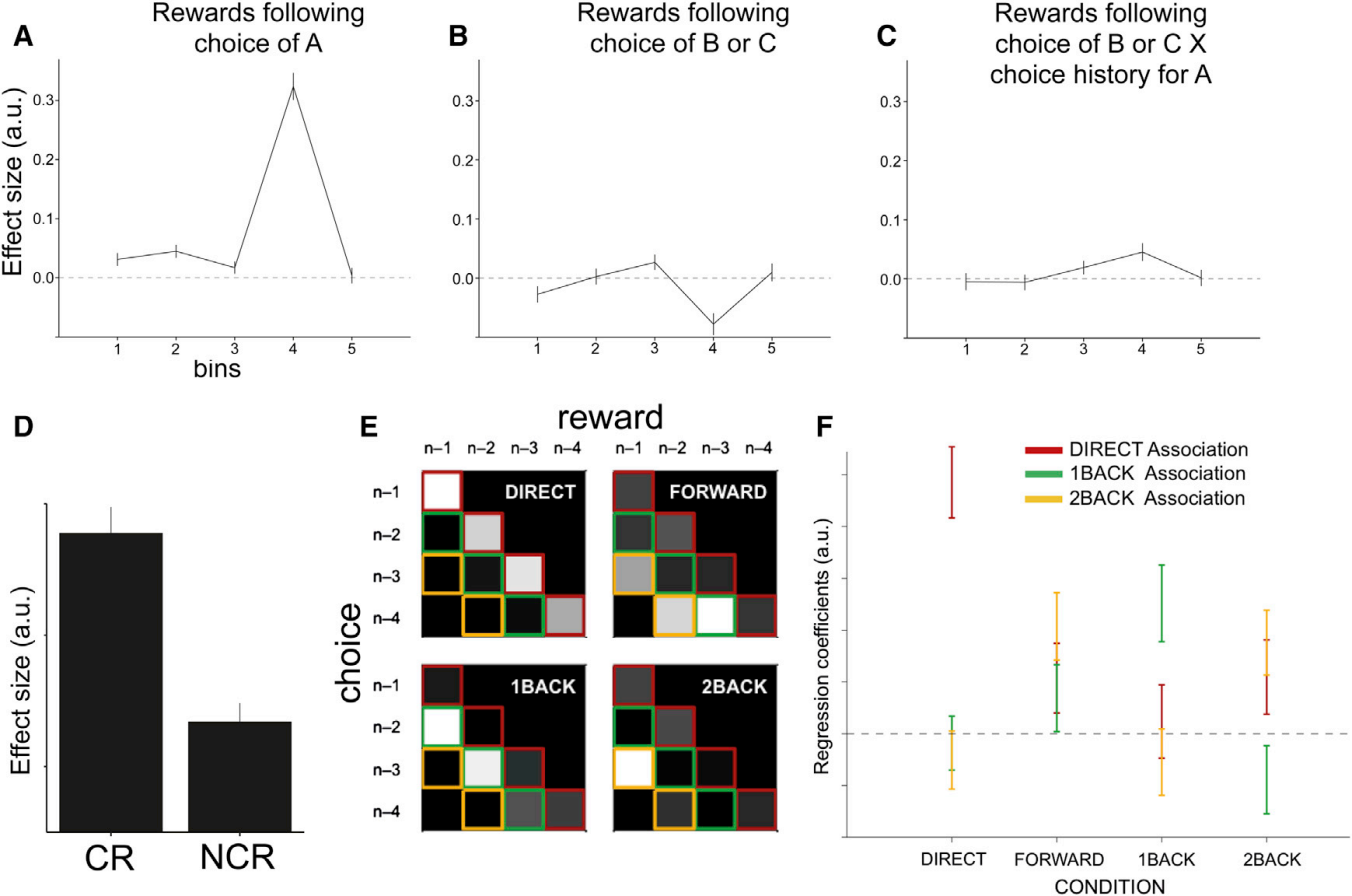
Behavioral Results (A–C) Logistic regression results of experiment 1. Figures show how choices (0/1) of the option on the current trial are influenced by past rewards following choices of same option A (A); different options B or C (B); and again different options B or C, but depending on how often same option A had been chosen in the past 30 trials (C), depending on when the reward occurred relative to choice (bin 1, 0–0.5 s; 2, 0.5–1.5 s; 3, 1.5–2.5 s; 4, 2.5–3.5 s; 5, 3.5–4.5 s before reward). Values are mean ± SEM (across participants) of the regression coefficients obtained from the logistic regression. (D) A separate linear regression shows that the average rate of responding in experiment 1 is, by definition, related to the rate of contingent rewards (CRs) but also to the rate of noncontingent rewards (NCRs), despite them being unrelated to behavior. (E and F) Behavioral results of experiment 2. Using multiple logistic regression, we tested whether our instructions reliably induced contingent and noncontingent learning. (E) Each box represents one condition, and each cell within a box represents a particular regressor. High parameter estimates are shown in white; low estimates in black. These regressors can be arranged into the lower quadrant of a square where the lead diagonal represents DIRECT learning (red), the next lower diagonal represents 1BACK learning (green), and the third diagonal 2BACK learning (yellow). For example, the first regressor in the top left box should receive loading if decisions under DIRECT instructions can be explained by a model in which any reward obtained on the previous trial (n – 1 column) is associated with the choice on that trial (n – 1 row). The plot shows that the DIRECT, 1BACK, and 2BACK conditions have predominantly yielded high parameter estimates in their respective red, green, and yellow regressors, while the FORWARD condition has led to loadings that are distributed across the different association types, as hypothesized. (F) Averaging across the corresponding associations (red, green, and yellow diagonals, respectively) shows that the three different associations load differently depending on the instructed condition. See also Figures S1–S4. All error bars represent SEM.

We looked for these effects in five time bins before a reward (time bins were as follows: bin 1, 0–0.5 s; bin 2, 0.5–1.5 s; bin 3, 1.5–2.5 s; bin 4, 2.5–3.5 s; bin 5, 3.5–4.5 s). As expected, the effects of rewards depended on the time bin in which choices fell (ANOVA, effect of bin, F_4,116_ = 94.01, p < 0.0001). Consistent with a robust contingent learning mechanism, choices of stimulus A in time bin 4 (t_29_ = 14.58, p < 0.0001) markedly increased the probability of choosing A again in the future (Figure 2A). Subjects were therefore able to assign credit for a reward to its causal choice despite the fact that there would often be another choice between the two events.

However, behavior was not exclusively driven by this precise contingent learning mechanism. Rewards following choices of A also increased the likelihood of future selections of A, but only if they occurred immediately after the choice (bin 1, t_29_ = 3.01, p = 0.0054; bin 2, t_29_ = 4.41, p = 0.0001), despite the fact that subjects were aware they were unrelated (Figure 2A). This involuntary spread of reward effect was specific to early time bins. While there was still a trend in bin 3 (t_29_ = 1.75, p = 0.091), rewards in later bin 5 had no effect on behavior (p > 0.77). Furthermore, the averaged effect in bins 1 and 2 was bigger compared to bin 3 (t_29_ = 1.76, p = 0.044, one-tailed) and to bin 5 (t_29_ > 2.43, p = 0.011, one-tailed). This effect of a reward not only reinforcing the choice that really led to its delivery but also other choices that occurred in close temporal proximity, was first described by Thorndike as early as 1933 (Thorndike, 1933) and has been termed “spread of effect.” Here, we refer to this spread of effect to proximal choices as PROX.

To examine statistical credit assignment mechanisms, we first asked whether subjects might misassign credit for a reward to the wrong choice if that choice had commonly been taken in the past (Walton et al., 2010), as if the reward is being credited to the average behavioral policy, and not to the particular choice that caused it. While contingent rewards that followed B or C choices made the future selection of A shapes less likely on average (Figure 2B), this was not true at times when the subject had selected A often in the recent past. Indeed, contingent rewards following B or C choices increased future A choices as an increasing function of the frequency of A choices in the past 30 trials (ANOVA, effect of bin, F_4,116_ = 2.45, p = 0.05; t test for bin 4, t_29_ = 3.14, p = 0.004; Figure 2C). That is, part of the credit for a reward following B or C choices was more likely to be misassigned to A the more often A had been selected in the recent past. We refer to this type of noncontingent learning as spread of effect to the recent history of choices (SoE_Ch_). Importantly, this cannot be explained by a mere autocorrelation in subjects’ choices. First, it predicts a switch away from the current choice of B onto the historical choice of A. Second, it is specific to rewarded choices. Third, it is specific to the contingent bin. Lastly, we included separate nuisance regressors in the regression model (see Experimental Procedures) to control for the main effect of choice history of A, the main effect of overall choice history, and the main effect of overall reward history. While the choice history of A had no effect (p = 0.3), the overall choice history had an effect, which, however, was negative and hence cannot explain the increased propensity to select option A (t_22_ = −4.1484, p = 0.0003). In addition, the overall rate of rewards increased subjects’ propensity to select A (t_22_ = 5.09, p < 0.00002). Next, we followed this latter effect up by asking whether subjects may be more likely to select shapes if the recent average reward rate was high, even if this was driven by noncontingent rewards that were unrelated to the subjects’ choices. We performed a separate regression that tested how the time-averaged rate of responding was dependent upon the time-averaged rate of contingent and noncontingent rewards (Supplemental Experimental Procedures, available online). By definition, the rate of responding depended on contingent rewards (as those are by design tied to responses). Importantly, however, response rates were also dependent on the rate of noncontingent rewards (t_29_ = 6.04, p < 0.00001; Figure 2D), indicating that the average rate of rewards increased the rate of responding. Thus, in addition to contingent learning, PROX, and SoE_Ch_, participants’ choices were also guided by a spread of effect to the recent history of rewards (SoE_Rew_).

Notably, despite some relations (maximum r = 0.38), the dominant contingent learning and the three noncontingent learning mechanisms (PROX, SoE_Ch_, and SoE_Rew_) were largely uncorrelated across subjects (Figure S1 for full correlation matrix), suggesting separable mechanisms. The behavioral effects reported here are derived from 30 subjects, which include the 23 subjects that underwent scanning and an additional 7 subjects that took part in the final version of the behavioral pilot. Note, however, that we obtain an identical pattern of results when repeating the same analyses following inclusion of only the 23 fMRI subjects (Figure S2A).

While in experiment 1 we aimed to investigate how multiple credit assignment strategies vary naturally in the extent they guide learning, in experiment 2 we selectively manipulated learning strategies through task instructions. Participants made choices between two fractal stimuli according to three types of instructions that changed for each block of trials (Figures 1, right, and S2B). In each block the probability of each choice leading to reward was constant (Supplemental Experimental Procedures) In DIRECT blocks, outcomes were contingent on the choice in the same trial. To dissociate contingency from choices made in the same trial with the outcome, in NBACK blocks, outcomes were delayed by a known number of trials (one or two). Hence, they were contingent on a previous, but specific, choice. In FORWARD blocks, rewards were delayed by a small random number of trials that was not known to the subject, such that outcomes could no longer be linked contingently to any specific causal choice. This ensured that unlike in NBACK blocks, it was not clear on which specific choices outcomes were contingent. Note that while subjects cannot learn contingently in the FORWARD condition, learning is still possible using statistical mechanisms. That is, while they do not know which one of the preceding four choices (current, immediately previous, two, or three trials past) caused the reward observed, they can still assign the credit to the average choice. Despite these three different types of instructions, the true contingencies were always structured according to the FORWARD condition. Thus, across all conditions, rewards were delayed, or projected forward, by a random number of trials. This simple manipulation controlled for a number of critical factors across conditions (Supplemental Experimental Procedures). This setup allowed us to interrogate fMRI signals reflecting contingency, as they contain sequences of trials that are identical between conditions in all respects except for the instructed contingency between choice and outcome. Thus, the only difference between conditions was in the instructed contingencies.

Despite the true contingencies being identical in each condition, participant behavior was consistent with the three different instruction sets. Logistic regression (Supplemental Experimental Procedures) revealed a condition-by-trial interaction (F_6,138_ = 8.62, p < 0.0001). Breaking these effects down showed that rewards increased future selections of the current choice in the DIRECT condition; the n − 1 and n − 2 choices in the 1BACK and 2BACK conditions, respectively; and all three previous choices in the FORWARD condition (Figures 2E and 2F). This demonstrates that subjects indeed deployed contingent learning in the DIRECT and NBACK conditions but noncontingent learning in the FORWARD condition, in which contingencies were unknown. Subjects were therefore able to exploit contingent learning mechanisms when contingencies were clearly discernible, but they were able to exploit noncontingent learning mechanisms when contingencies were unclear. Importantly, we ensured that the only difference between conditions was the instructed contingencies, while keeping all possible other factors comparable between conditions, such as subjects’ rate of learning, the number of rewards earned, errors committed, and response times (Supplemental Experimental Procedures; Figures S3 and S4).

### Signals Supporting Contingent Learning in lOFC

To search for brain regions linked to contingent learning, we examined the BOLD response at the time of the outcome in the conditions where subjects received instructions about the precise associations between stimuli and rewards: contingent and noncontingent rewards in experiment 1; reward and no reward in the DIRECT and NBACK conditions of experiment 2. We predicted that the lOFC would underlie precise contingent learning and, therefore, that subjects with more activity in this brain region would rely less on noncontingent learning.

**Figure 3:**
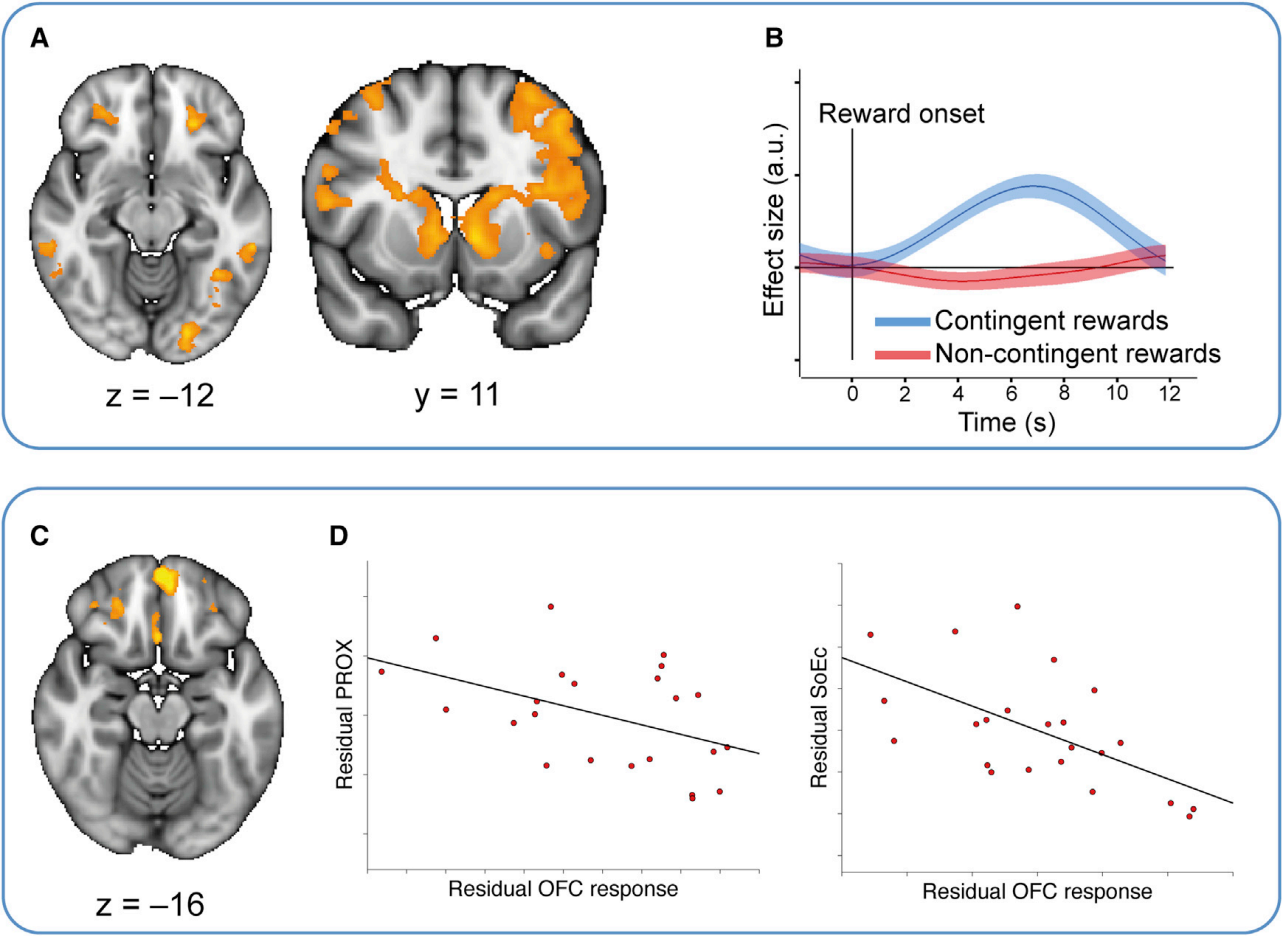
Contingent Reward Responses and Relation to Contingent Learning in Experiment 1 (A) Whole-brain results for the contrast contingent- noncontingent rewards in experiment 1. (B) Inspection of the BOLD signal at the peak coordinate in lOFC shows that this region responds selectively to contingent, but not noncontingent, rewards. Solid lines show the mean and shaded areas the SEM of the regression coefficients across subjects. The black vertical line represents the time of outcome delivery. Values are mean ± SEM of regression coefficients across subjects. (C) Regression of the contrast in (A) against contingent learning versus PROX + SoE_Ch_ reveals that contingent reward responses in lOFC correlate with contingent learning behavior. (D) Parameter estimates were extracted from the peak coordinate of the contrast in (A) and related to the different learning mechanism. The plots show that lOFC responses to contingent rewards are negatively related to noncontingent learning via PROX and SoE_Ch_. The correlations are partial correlations, that is, after regressing out the effects of the respective other learning mechanisms from both parameters of interest. See also Figure S5 and Table S1.

We first harnessed the interindividual variability in learning strategies in experiment 1 and investigated their neural correlates. We contrasted BOLD responses between contingent and noncontingent rewards. While both serve as rewards, it is only during the former that a contingency between the reward and a choice has to be established. As the contingent rewards were the principal focus of subject attention, it is not surprising that this contrast revealed a large network of brain regions (Figure 3A; Table S1; whole-brain cluster corrected at p < 0.001; cluster size threshold, p < 0.05), including the lOFC (MNI xyz = –24 mm, 35 mm, –12 mm, z max = 5.1 and xyz = 26 mm, 40 mm, –10 mm, z max = 4.59) and bilateral striatum, in particular in the ventromedial caudate nucleus (MNI xyz = –8 mm, 11 mm, –1 mm, z max = 5.16; and xyz = 10 mm, 14 mm, 1 mm, z max = 4.69; Figure 3A). Visualization of this difference effect in the lOFC revealed that it was driven exclusively by the positive (contingent rewards) portion of the contrast, and not the negative (noncontingent rewards) portion (Figure 3B). From each subject’s behavior, we computed the ratio of contingent learning to noncontingent mechanisms. We asked whether the aforementioned BOLD contrast [contingent rewards – noncontingent rewards] in any voxels would predict the extent to which subjects relied on precise contingent relative to noncontingent learning strategies (contingent learning versus PROX + SoE_Ch_). Here we considered the two noncontingent mechanisms that were contributed to by the contingent rewards (PROX and SoE_Ch_), reflecting the fact that the BOLD contrast at the first level was derived from these rewards (PROX can arise by misattribution of either contingent or noncontingent rewards to proximal choices; SoE_Ch_ specifically arises by misattribution of contingent rewards to the average choice history. In contrast, SoE_Rew_ is specifically defined as the effect of noncontingent rewards). The only brain region to show a significant effect across subjects was in the OFC, including the lOFC (p < 0.01, cluster-based correction at p < 0.05; Figure 3C). Furthermore, extracting parameter estimates from the peak lOFC coordinate (from the main contrast contingent minus noncontingent rewards) revealed that lOFC activity was inversely related to both noncontingent learning mechanisms (r = –0.46, p = 0.03 and r = –0.58, p = 0.0034, for PROX and SoE_Ch_, respectively; Figure 3D), with no significant difference (t_22_ = −0.15, p > 0.55). Subjects with strong lOFC responses to contingent rewards were therefore less likely to exhibit either form of noncontingent learning relative to accurate contingent learning.

Experiment 2 allowed us to further isolate lOFC’s role in contingent learning, as it contained sequences of trials identical in all respects except for contingency. We considered sets of three trials pertinent to any particular outcome (+ and – denote reward and nonreward outcomes, respectively): the past trial (n − 1 or n − 2), the current trial (n) and the following trial (n + 1). For example, in the sequence “BA+B,” the subject switched from a B choice in the previous trial to an A choice in the current trial, received a reward, and then switched back to B the following trial. In order to examine contingency, we examined BOLD activity at the time of this outcome and contrasted trials in which the “following” choice respected the contingencies of the outcome against those where it did not. For example, BA+A and BA-B are contingent sequences in DIRECT blocks because the subject acted in accordance with the outcome (stay with rewarded A or switch back from unrewarded A). By contrast, in NBACK blocks, these same sequences are noncontingent because the outcome pertained to the preceding B, rather than the proximal A. Similarly, [BA-A, BA+B] are noncontingent sequences in DIRECT blocks but contingent sequences in NBACK blocks. To control for block differences, [AA+A, AA-B] are contingent and [AA-A, AA+B] noncontingent in all conditions. It is notable that comparisons between contingent and noncontingent sequences are controlled both within and across conditions for choices, outcomes, and switches but, on average, distinguish outcomes that caused contingent learning from those that did not.

**Figure 4:**
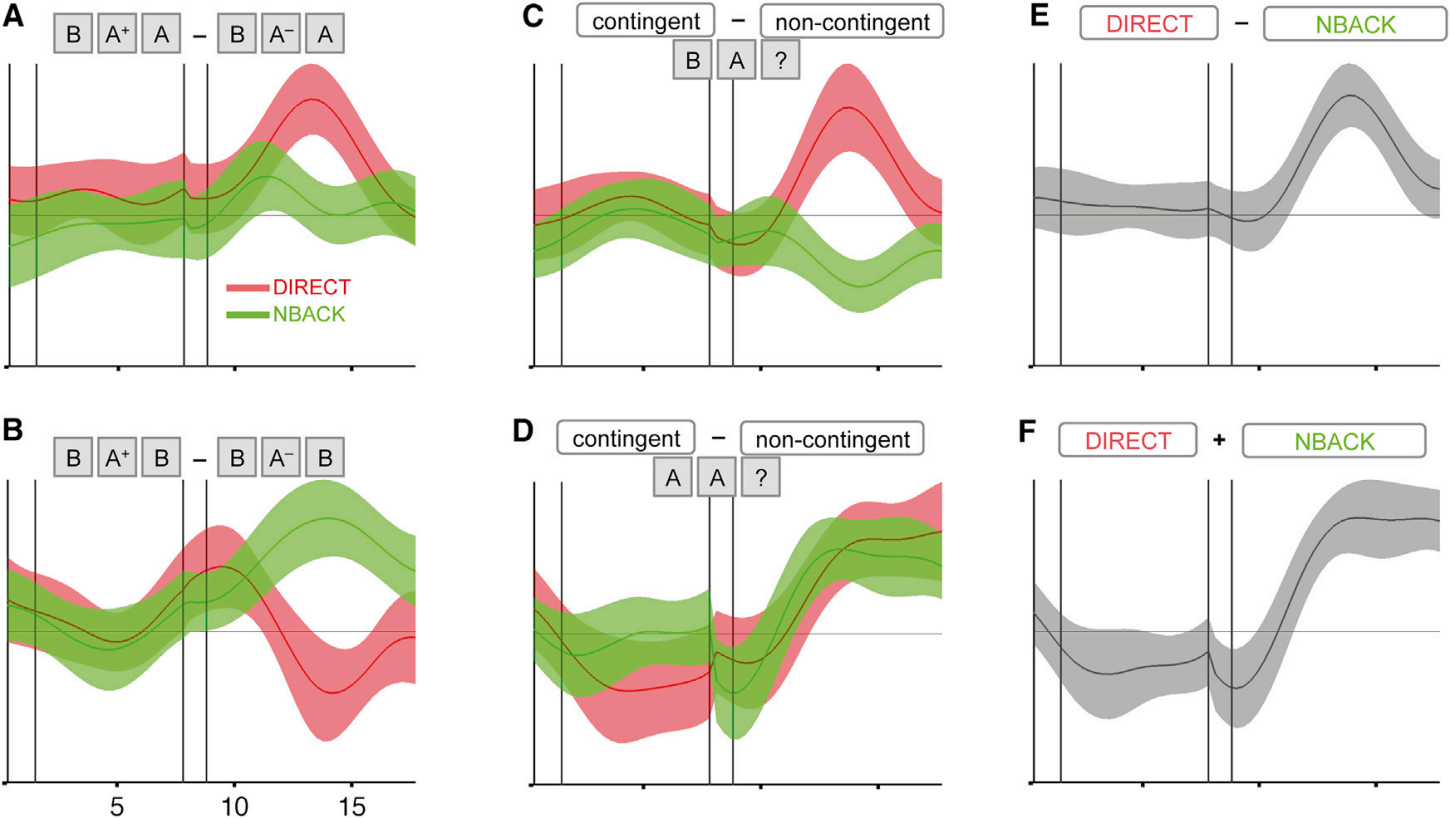
Analysis Testing Whether the OFC Signal in Experiment 2 Fulfils the Criteria of a Signal Encoding Associations between Outcomes and Their Causal Choices Each panel shows the observed temporal evolution of a GLM contrast over intratrial time (contrast parameter estimates ± SEM). Data are averaged across all OFC voxels that survived the (orthogonal) contingency contrast on AA? triplets. Outcomes (reward/nonreward) refer to the outcome of the middle trial in each triplet. Vertical bars separate decision, delay, outcome, and interval phases. (A) Consistent with a signal encoding contingent associations between choices and outcomes, only in the outcome phase of DIRECT trials do contingent associations elicit an increased lOFC signal. (B) In NBACK blocks, it is the noncontingent trials that yield lOFC activity. (C) Taken together, contingent and noncontingent trials lead to exactly opposite signals in DIRECT and NBACK blocks (addition of the contrasts [BA+B - BA−B] + [BA−B - BA+B]). (D) In AA? triplets, contingent choices are identical in DIRECT and NBACK blocks; accordingly, lOFC shows the same effect in both conditions (contrasting [BA+B - BA−B] - [BA−B - BA+B] triplets). (E) BA? triplets show a highly significant contingency effect in the lOFC. (F) Thus, AA? triplets show an equally strong contingency effect as BA? triplets. Overall, the figure shows that lOFC activity is incompatible with predictions made by the reward and reward prediction error hypotheses but corresponds precisely to the predictions made by the contingency hypothesis. Note that all plots were produced by right-aligning data from the decision phases so as to line up with the decisions themselves. The jittered duration of the delay phase thus causes a discontinuity between the delay and the monitor phases in this visualization. See also Figure S6.

We extracted data from an ROI in the lOFC selected from an orthogonal contrast (see Supplemental Experimental Procedures for ROI selection). In line with a contingency-related response, BA+A caused greater lOFC activity than BA-A in DIRECT, but not NBACK, blocks (Figure 4A; difference, t_23_ = 3.28, p = 0.002), and BA+B caused greater lOFC activity than BA-B in NBACK, but not DIRECT, blocks (Figure 4B; difference, t_23_ = 3.03, p = 0.003). Combining these two effects according to DIRECT contingencies revealed a positive effect in DIRECT blocks (t_23_ = 2.45, p = 0.01) and a negative effect in NBACK blocks (t_23_ = −2.72, p = 0.006), where contingencies were reversed (Figure 4C). Notably, repeating this analysis for sequences that began AA, where contingencies were identical across blocks (Figure 4D), revealed a positive response in both conditions (DIRECT, t_23_ = 2.56, p = 0.009; NBACK, t_23_ = 2.74, p = 0.006; difference not shown). Hence, across all tests, lOFC responses were aligned with contingencies rather than rewards or behavior (Figure 4E; t_23_ = 3.77, p = 0.0005; Figure 4F; t_23_ = 3.65,p = 0.0007). This is particularly notable in light of previous theories of lOFC function that have argued for error processing (Kringelbach and Rolls, 2004, Fellows, 2007) or behavioral switching (Jones and Mishkin, 1972, Dias et al., 1997, Chudasama and Robbins, 2003) to be cardinal functions of the region.

Further to the effects in the lOFC, it is noteworthy that at the whole-brain level, contrasting contingent with noncontingent trials revealed a network of brain regions very similar to that found in experiment 1 when contrasting contingent with noncontingent rewards (Figure S6). In particular, these included lOFC (MNI xyz = –24 mm, 40 mm, –16 mm, z max = 3.8), bilateral ventral striatum (MNI xyz = ±16 mm, 10 mm, –14 mm, z max = 4.1), and lateral prefrontal cortex (MNI xyz = 42 mm, 32 mm, 22 mm, z max = 4.1).

### Amygdala Responses Mediate Noncontingent Learning

Across experiments, neural signals were therefore consistent with contingent learning mechanisms in a network of fronto-striatal brain regions, with the strongest behavioral impact in the lOFC. However, in experiment 1, subjects also deployed three noncontingent learning strategies, PROX, SoE_Ch_, and SoE_Rew_. On the basis of previous lesion data from both macaques and rodents (Stalnaker et al., 2007, Rudebeck and Murray, 2008), we hypothesized that the amygdala might play a key role for at least some of these noncontingent mechanisms.

Amygdala lesions facilitate reversal learning in monkeys (Rudebeck and Murray, 2008) and restore the ability to perform reversals after OFC lesions in rodents (Stalnaker et al., 2007). Since OFC reversal deficits are reflective of deficits in precise contingent learning (Walton et al., 2010), it is conceivable that amygdala activity at the time of a reward might downweight precise associations in favor of statistical ones. In our experiment 1, such an argument makes two predictions. First, it predicts that amygdala activity at the time of contingent rewards would lead to less contingent and greater statistical learning (which is maladaptive in the current task). Second, it predicts that amygdala activity at the time of free rewards would mean these free rewards were less likely to be treated as contingent rewards (which is adaptive in the current task).

**Figure 5:**
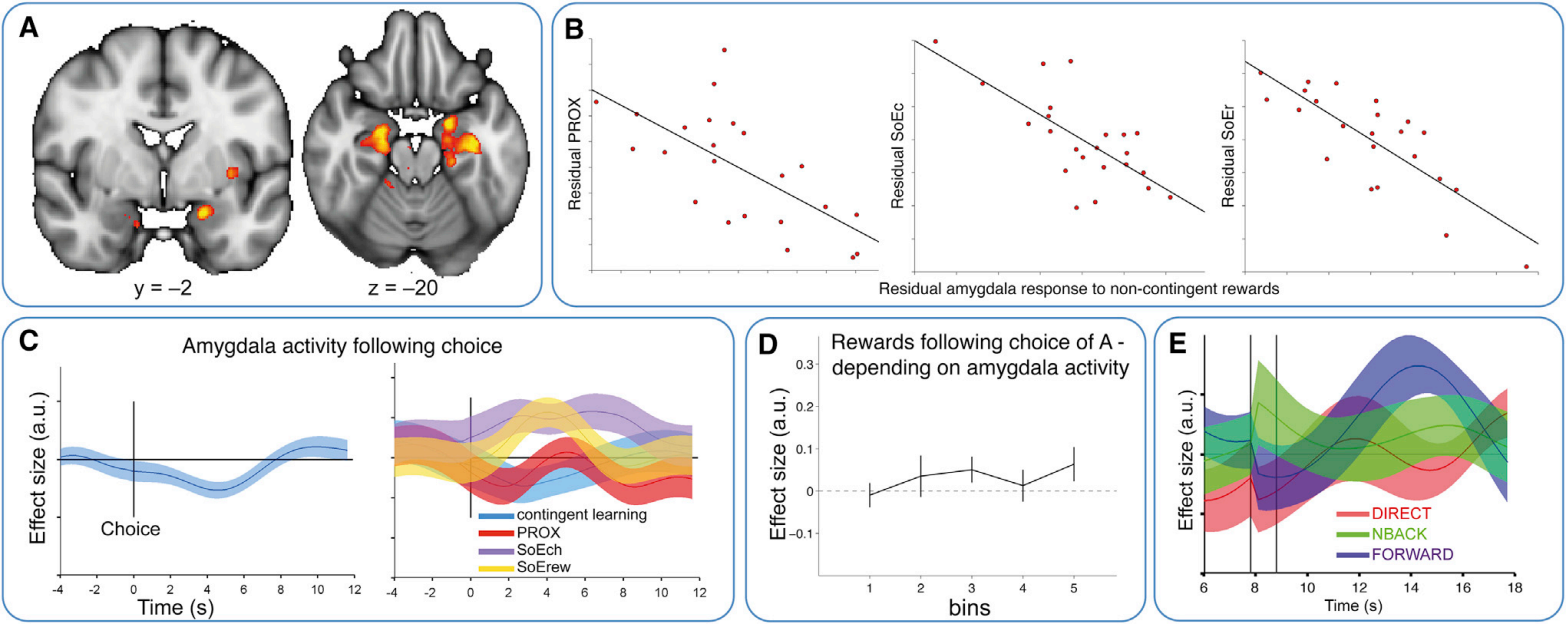
Amygdala and Noncontingent Learning (A) In experiment 1, stronger amygdala responses to noncontingent rewards correlate with better contingent relative to noncontingent learning. (B) Extraction of parameter estimates from the peak coordinate of the above contrast in (A) shows that amygdala responses to noncontingent rewards in experiment 1 correlate negatively with all three noncontingent learning mechanisms, albeit the correlation with SoE_Rew_ was more pronounced than that with either SoE_Ch_ or PROX (see main text). The correlations are partial correlations, that is, after regressing out the effects of the respective other learning mechanisms from both parameters of interest. (C) Following a choice, the amygdala signal was suppressed (left). Amygdala activity after a choice (in anticipation of a contingent reward) correlated positively with SoE_Ch_ and SoE_Rew_ (right), meaning that a lack of amygdala suppression was associated with misassignment of the following reward via one of these noncontingent mechanisms. (D) On a trial-by-trial level, credit for a reward following choice of A was likely to be misassigned to one of the noncontingent bins when amygdala activity was high in the period between choice and reward. (E) In experiment 2, amygdala was exclusively reward sensitive in the FORWARD condition, the only condition where learning was only possible from spreading credit for a reward to the average choice history. The graph shows the evolution of a simple “reward-no reward” contrast over intratrial time as in Figure 4, taken from the peak coordinate from experiment 1 shown in (A). Solid lines in (C) and (E) show the mean; shaded areas and error bars in (D) represent the SEM of the contrast estimates across subjects. The black vertical lines in (E) represent the time of choice and outcome delivery, respectively.

To address this second prediction, we searched for brain regions whose responses to noncontingent rewards were related to noncontingent learning. Consistent with the mechanism described above, we found clusters bilaterally in the amygdala and anterior hippocampus that exhibited a negative correlation with noncontingent learning (MNI xyz = –19 mm, –3 mm, –21 mm, z max = 4.06 and xyz = 24 mm, –9 mm, –18 mm, z max = 4.25; whole-brain cluster corrected at p < 0.01; cluster size threshold, p < 0.05; Figure 5A). Subjects with large responses to free rewards in these regions were thus unlikely to inaccurately treat these free rewards as contingent. Extracting parameter estimates from the peak coordinate revealed that subjects with strong amygdala responses to noncontingent rewards relied less on all three noncontingent learning mechanisms (r = –0.67, –0.71, and –0.81 for PROX, SoE_Ch_, and SoE_Rew_, respectively; all p < 0.0006; Figure 5B). Notably, while the amygdala response correlated equally strongly with PROX and SoE_Ch_ (t_22_ = 0.81, p > 0.4), it correlated more strongly with SoE_Rew_ compared to both SoE_Ch_ and PROX (t_22_ = 4.72 and t_22_ = 4.41, p < 0.0003). Moreover, in contrast to the lOFC, contingent rewards had no significant effect in the amygdala and did not predict any marker of learning (all t < 0.73, p > 0.47).

We extracted data from the peak amygdala coordinate from this cluster to test our first prediction. Amygdala activity should be suppressed to allow contingent learning from contingent rewards. We first note that when taken on average over the group, amygdala activity is indeed suppressed after subjects make a response in anticipation of a contingent reward (Figure 5C; t_22_ = –3.75, p = 0.001). Furthermore, across subjects, this suppression is negatively related to the two statistical learning mechanisms (t_22_ = 2.7 and t_22_ = 3.06, p = 0.013 and p = 0.006; SoE_Ch_ and SoE_Rew_, respectively). Subjects who do not exhibit this suppression will learn statistically, not contingently, from the contingent rewards. Despite the absence of an effect on PROX, the effect survives the averaging over all three noncontingent mechanisms (t_22_ = 3.68, p = 0.0013).

In order to strengthen this argument within subjects, we designed a novel analysis strategy that examined the relationship between this amygdala suppression and noncontingent learning on a choice-by-choice basis within a single subject. We fit separate hemodynamic response functions to the amygdala activity after every button press. This resulted in a vector of parameters describing the amygdala response to each button press. We then performed a new behavioral regression like the regression in Figures 2A–2C, but now each behavioral regressor was paired with a second regressor: the interaction of itself and the (demeaned) amygdala response. This regression therefore asks whether the amygdala responses predict how the reward will impact future behavior. Despite the noisy nature of single-trial fMRI fits, a pattern emerged in which increased amygdala activity before rewards (the absence of amygdala suppression) led to noncontingent learning. If amygdala activity was high following choice of A, then rewards in the noncontingent bins made future choices of A more likely (Figure 5D; average over all bins, t_22_ = 2.58, p = 0.017).

Together, these results suggest that amygdala responses in the anticipation and delivery of reward lead to a reduction of precise contingent learning from that reward. More activity to free rewards makes it less likely that those rewards will be falsely treated as contingent. Activity is suppressed in anticipation of contingent rewards. The absence of this suppression makes it more likely that contingent rewards will be treated statistically (rather than contingently) and more likely that intervening free rewards will be mistaken for contingent ones. We investigated this effect further by examining amygdala reward responses in experiment 2, which included an explicit experimental manipulation to control contingent learning. We extracted signal from the above peak coordinate identified in experiment 1 (MNI xyz = –19 mm, –3 mm, –21 mm) and compared responses in the DIRECT and NBACK conditions, where rewards could be attributed to particular choices in the past, to those in the FORWARD condition, where rewards could not be assigned to any particular choice but nevertheless reinforced current broad behavioral policies (statistical learning). While activity in the amygdala did not distinguish rewards from unrewarding outcomes in either of the two contingent conditions (t_23_ = 1.68 and 0.95, p > 0.1 and p > 0.34; DIRECT and NBACK, respectively), it exhibited a clear reward effect in the noncontingent FORWARD condition (Figure 5E; t_23_ = 3.46, p < 0.003; difference between FORWARD and DIRECT, t_23_ = 2.35, p = 0.014, one-tailed; difference between FORWARD and NBACK, t_23_ = 1.84, p = 0.04, one-tailed).

### lOFC Interactions with Ventral Striatum

**Figure 6:**
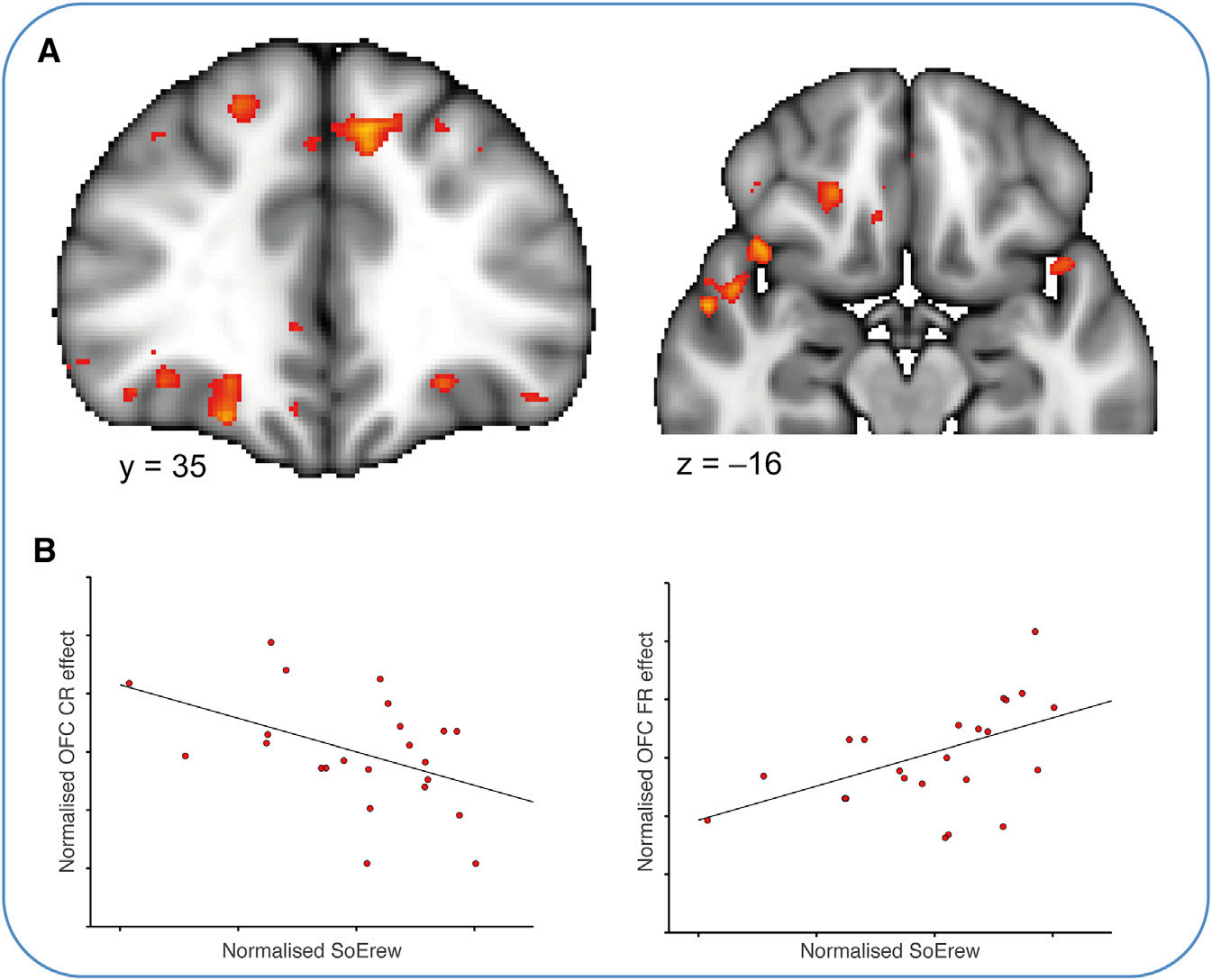
Connectivity of lOFC with VMS (A) VMS connectivity with lOFC during contingent versus free rewards is related to better contingent relative to noncontingent learning. (B) Increased VMS-lOFC connectivity during contingent rewards is related to decreased SoErew, whereas the opposite pattern is found for connectivity during free rewards. Correlations are partial correlations, that is, after regressing out the effects of the respective other learning mechanisms from both parameters of interest.

In our main contrast of contingent versus free rewards, we found, in addition to the effect in lOFC, a prominent effect in ventromedial striatum (VMS). While this effect, unlike the lOFC effect, did not correlate with behavior across subjects, it is plausible that interactions between lOFC and VMS underlie precise contingent learning. VMS receives dense projections from lOFC (Selemon and Goldman-Rakic, 1985), and, together, the two structures are part of a key circuit underlying goal-directed learning (Yin and Knowlton, 2006). We therefore performed a psychophysiological interaction analysis (PPI, see Supplemental Experimental Proceduresfor details) to test whether increased coupling between lOFC and VMS during contingent versus free rewards supports contingent learning. We extracted data from the peak coordinate in the VMS (MNI xyz = –8 mm, 11 mm, –1 mm and xyz = 10 mm, 14 mm, 1 mm) and searched for regions in which coupling with this seed region was related to individual differences in learning styles. In line with our hypothesis, we found regions in bilateral lOFC in which higher coupling with VMS during contingent versus free rewards was related to better contingent relative to noncontingent learning (uncorrected at p < 0.001, MNI xyz = –24 mm, 36 mm, –11 mm, z max = 3.17 and xyz = 22 mm, 36 mm, –19 mm, z max = 3.58; Figure 6A). We extracted parameter estimates from this coordinate to test whether this effect could be specifically related to connectivity during receipt of contingent or free rewards. We found that connectivity during contingent rewards was associated with diminished SoE_Rew_ (r = –0.47, p = 0.029), whereas free reward connectivity was related to increased SoE_Rew_ (r = 0.5, p = 0.016; Figure 6B). The other learning parameters, while generally showing a similar pattern, did not reach significance.

### Midbrain and Dorsolateral Striatal Reward Responses Promote Noncontingent Learning

**Figure 7:**
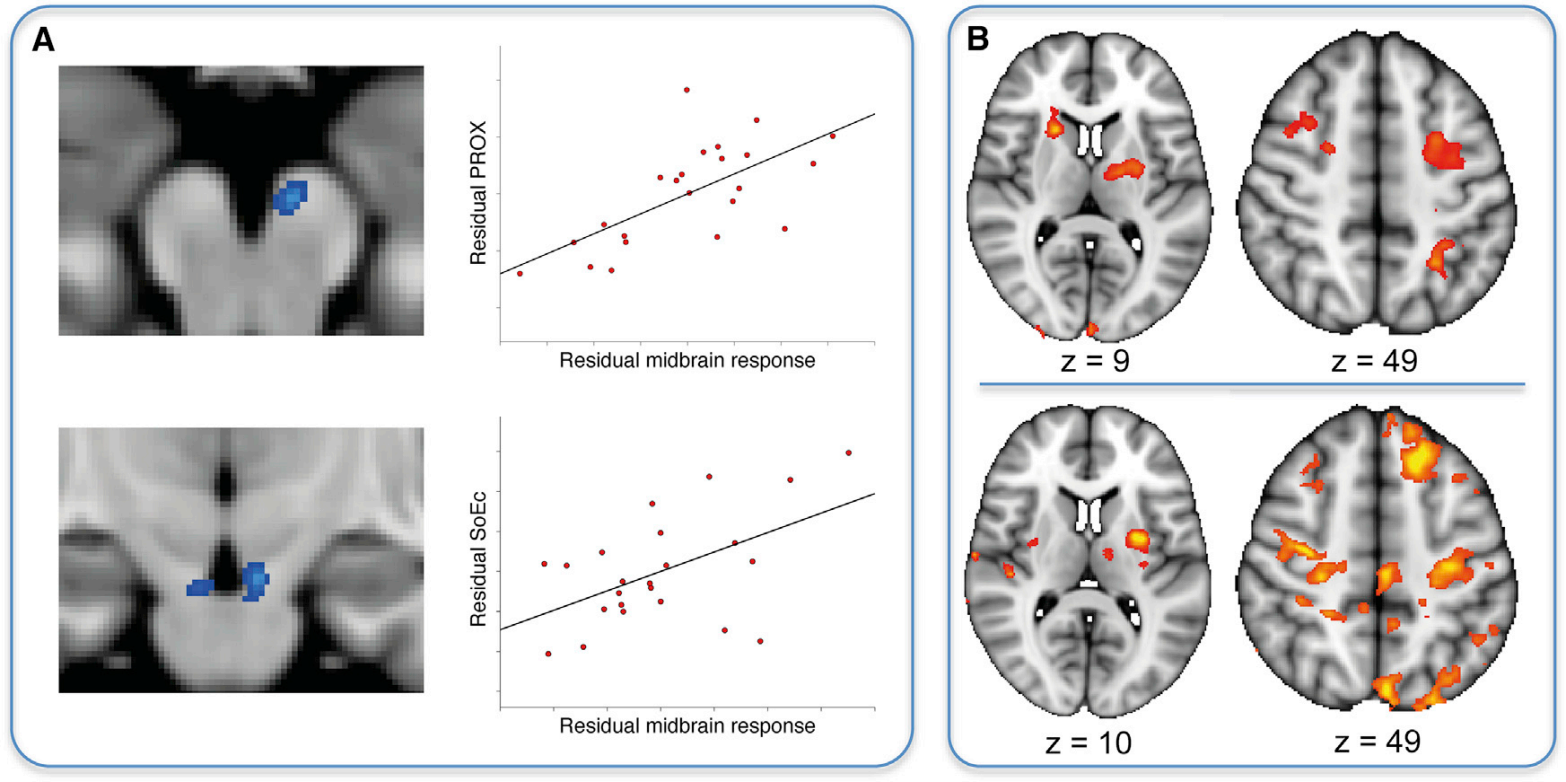
Relationship of Midbrain and Dorsal Striatal Reward Responses to Noncontingent Learning in Experiment 1 (A) Midbrain responses to contingent rewards in a region consistent with the location of substantia nigra and ventral tegmental area correlate negatively with the degree to which subjects’ behavior was guided by contingent learning as opposed to either PROX or SoE_Ch_—the exact opposite pattern of what was observed in lOFC (see Figures 3C and 3D). Contingent reward responses at the peak location were strongly related to both PROX and SoE_Ch_; however, the correlation with PROX tended to be stronger than that with SoE_Ch_. (B) A parametric contrast revealed that rewards elicited a stronger response the sooner they occurred following a choice (upper row). In areas associated with model-free learning such as putamen and associated motor cortical areas, this effect was strongly related to the extent subjects’ behavior was guided by PROX (bottom row).

In experiment 1, we found that credit for a contingent reward was not only assigned correctly to the causal choice but also incorrectly to temporally proximal choices (PROX; Figure 2A) and to the average history of recent choices (SoE_Ch_; Figure 2C). Using the same contrast [contingent rewards − noncontingent rewards] as above for lOFC, we found a region of the midbrain showing the opposite relation to behavior as lOFC. In this midbrain region, consistent with the location of dopaminergic cell groups of the ventral tegmental area (VTA) and pars compacta of the substantia nigra (SN_C_), responses to contingent rewards correlated negatively with the degree to which subjects deployed precise contingent learning as opposed to both PROX and SoE_Ch_ (MNI xyz = –5 mm, –16 mm, –19 mm, z max = –3.66 and xyz = 6 mm, –15 mm, –21 mm, z max = 3.48; p < 0.001, uncorrected; Figure 7A). Please note that this contrast did not survive cluster-based thresholding, which, however, is unsurprising given the small size expected of midbrain clusters. We extracted parameter estimates from the peak location of this correlation to test if this effect could be specifically related to PROX, or if it was more generally related to overall noncontingent learning. At this peak location, responses were strongly related to PROX (r = 0.66, p < 0.001; Figure 7A) and to SoE_Ch_ (r = 0.52, p = 0.01), but not to SoE_Rew_ (r = 0.23, p = 0.28). Furthermore, direct comparison revealed that midbrain activity was, by trend, more strongly related to PROX than to SoE_Ch_ (t_22_ = 1.65, p = 0.0566). These results suggest that VTA/SNC responses to contingent rewards may lead to part of the credit for these rewards being misassigned to both proximal choices and to the average choice history.

We reasoned that PROX might arise because the close temporal coincidence of the reward-evoked dopamine release with a motor command in regions such as dorsolateral striatum would “stamp in” such stimulus-response associations (Redgrave and Gurney, 2006, Yin and Knowlton, 2006). Following this logic, the magnitude of reward responses in sensorimotor striatal regions will depend on the delay between choice of a stimulus and the outcome, with sooner rewards being more effective. We investigated this by setting up a parametric contrast in which all rewards were modulated by the time elapsed since the last action. We found that rewards delivered soon after a choice evoked responses in the putamen, the rostral caudate, and the bilateral premotor cortex (p < 0.01, cluster corrected at p < 0.05; Figure 7B, upper row). This network, unlike the circuitry involving lOFC and ventral striatum responsive to contingent rewards, has been implicated in learning of stimulus-response habits in a habitual fashion (Yin and Knowlton, 2006). Importantly, we found that this effect in bilateral putamen (MNI xyz = –30, –3, 10, z max = 4.09 and xyz = 29, –7, 5, z max = 3.43) and bilateral motor cortex (MNI xyz = –29, –21, 45, z max = 3.9 and xyz = 35, –14, 53, z max = 4.43) was stronger in subjects who relied more on PROX (p < 0.01, cluster corrected at p < 0.05; Figure 7B, bottom row). To test whether these timing-dependent reward effects were specifically related to PROX or to both PROX and SoE_Ch_, we extracted parameter estimates from independent peak coordinates in the putamen and motor cortex to test for differential correlation. The peak was selected from a contrast of the correlation with both PROX and SoE_Ch_, thus avoiding bias toward either of the two mechanisms. In both putamen and motor cortex, the response was strongly related to both PROX (t_22_ = 5.04 and t_22_ = 5.32, p < 0.00005) and SoE_Ch_ (t_22_ = 2.82 and t_22_ = 2.65, p < 0.015), but not SoE_Rew_ (p > 0.2). Direct contrasts further revealed a significantly stronger correlation with PROX compared to SoE_Ch_ in both areas (t_22_ = 2.59 and t_22_ = 2.9, p < 0.02). Thus, reward modulated activity in both putamen and motor cortex depending on the timing relative to a choice, and this modulation was related to noncontingent learning in both structures. While proximity-based reward responses correlated with both PROX and SoE_Ch_, they did not correlate with SoE_Rew_, and the correlation with PROX was more pronounced than that with SoE_Ch_. This provides further evidence that PROX and SoE_Ch_ are not only dissociable behaviorally but also neurally. It is also important to note that this pattern is different to that found in the lOFC, where responses to contingent rewards were negatively related to both PROX and SoE_Ch_ to the same extent. Thus, while contingent reward responses in lOFC appear to generally suppress noncontingent learning from contingent rewards, proximity-dependent reward responses in putamen and motor cortex appear to be predominantly associated with PROX, i.e., with spreading credit for a reward to very recent choices.

## Discussion

We have shown that in a dynamic environment, the choices of healthy participants are guided by both precise contingent and noncontingent learning mechanisms that are separable both behaviorally and at the neural level. Behavior was dominated by learning that reflected the true choice-outcome contingencies. Such learning appeared to rely in part on lOFC. However, we were also able to identify other learning mechanisms that assigned outcomes to incorrect choices. Two of them were statistical learning mechanisms that learned through time-averaged choices and rewards. These behaviors appeared to rely in part on amygdala responses both in anticipation and receipt of rewards. Lastly, we identified a “heuristic” learning mechanism whereby rewards were inaccurately paired with choices that immediately preceded them. This direct action-outcome pairing was predicted by responses in the motor corticostriatal circuitry.

In healthy macaques, precise contingent learning is usually so powerful that it dwarves the influence of other learning mechanisms. The contribution of these noncontingent mechanisms only becomes evident after lesions to lOFC (Walton et al., 2010). Likewise, for healthy human volunteers, credit assignment is trivial on standard reinforcement learning tasks, where there is usually only one choice and one outcome per trial. By breaking with the typical trial-based structure and by randomly delivering noncontingent rewards, we were able to create a scenario that is more akin to a naturalistic environment, where several responses could be candidate actions for a given outcome. This allowed noncontingent learning mechanisms to become more pronounced and, thus, to be isolated along the dominant contingent learning mechanism. It is likely that in real-life situations with many candidate actions and intervening outcomes, the effect of these noncontingent mechanisms is even more pronounced. We identified three such noncontingent mechanisms. First, rewards that occurred very close in time to a particular action tended to reinforce that action whether or not it caused the reward (PROX). This heuristic mechanism is reminiscent of the steeply diminishing effect of reinforcement with increasing delay between conditioned stimulus or instrumental action and reinforcement (Kamin, 1961, Dickinson et al., 1992). It also bears a resemblance to the emergence of superstitious behaviors during operant conditioning, where behaviors unrelated to reward are often reinforced due to their temporal proximity to reward delivery (Skinner, 1948, Devenport, 1979). Second, subjects were likely to assign credit for a reward to a choice that was frequently selected in the recent past, whether or not it was causally related to the reward (SoE_Ch_). Third, subjects were likely to make choices more frequently during periods when they were rewarded frequently, even if those choices did not cause the reward (SoE_Rew_). While such statistical mechanisms can be catastrophic when contingencies change abruptly from one trial to the next, they do not impede learning in situations where contingencies are stable or smoothly varying (Walton et al., 2010). Indeed, related strategies based on average recent reward rates may be beneficial in foraging-style decisions, which learn only the relative value of pursuing or switching from a current ongoing strategy (Charnov, 1976). There are also many real-world examples where learning via PROX or SoE_Ch_ may be adaptive. Contingent learning may be led astray when assumptions about the causal structure of the task are inaccurate, as is the case in the confirmation bias (Doll et al., 2011). In situations such as motor learning, PROX is adaptive because causality is closely tied to temporal proximity. Likewise, statistical learning mechanisms that average long-term rewards and choices are adaptive in situations where it is unclear which precise outcomes relate to which precise choices.

The noncontingent learning mechanisms we investigate in this study do not reflect the loss of all credit assignment between stimulus and reward. Rather, credit assignment in these mechanisms happens either statistically (because stimuli have often been chosen during rewarding periods) or heuristically (because a reward happened to occur immediately after a stimulus was chosen). Indeed, what is unique about the precise contingent mechanism is that the credit for an individual reward is attributed to a precise individual selection of the relevant stimulus in an appropriate fashion, reflecting the (accurate) knowledge that the choice caused the reward to occur. This knowledge may be gained through instructions, as in the current report, or through extensive experience on the learning problem, as in the original report of lesions to macaque OFC (Walton et al., 2010). The factors that determine the relative contribution of precise contingent learning and noncontingent mechanisms are, to our knowledge, not known. It is possible that uncertainty about the causal structure of the world is one factor that promotes statistical learning.

The contingent and noncontingent learning mechanisms we identified were anatomically separable. While a large network of brain regions was more active during receipt of contingent as opposed to noncontingent rewards in experiment 1 (likely reflecting an attentional effect), lOFC was the only one of these areas to show a clear relationship to contingent learning. Subjects with the strongest responses to contingent rewards in this region were least likely to misassign these rewards via either PROX or SoE_Ch_. Similarly, connectivity between lOFC and VMS during receipt of contingent rewards was related to better contingent learning. Furthermore, experiment 2 allowed us to dissect the OFC reward signal in precise detail. By comparing triplets of trials that were identical in all respects except for the instructed contingencies, we could show that the same reward in a given triplet had opposite effects depending on whether the reward had to be associated with the choice on the current trial or with the alternative choice on the previous trial. Thus, our results are consistent with lOFC encoding the exact kind of signal required to solve the credit assignment problem, that is, to associate a reward with the choice that caused it (Sutton and Barto, 1998). These data are in agreement with studies showing that OFC neurons flexibly encode the reward-predictive properties of stimuli (Thorpe et al., 1983, Schoenbaum et al., 1998, Tremblay and Schultz, 1999, Padoa-Schioppa and Assad, 2008, Morrison and Salzman, 2009). Accordingly, lesions to the OFC reliably produce deficits in adjusting behavior to changes in stimulus-outcome associations (Mishkin, 1964, Jones and Mishkin, 1972, Dias et al., 1996, Izquierdo et al., 2004). These deficits resulted from credit being distributed inappropriately to choices that were made proximal in time to the outcome and to the average choice history (Walton et al., 2010). This strongly suggests that OFC is essential for contingent learning. Our data support this view: (1) BOLD signals in lOFC displayed the hallmarks of a signal encoding contingent associations between outcomes and the choices that caused them, and (2) lOFC responses to contingent rewards were related to learning strategies.

The ability to learn causally in reinforcement learning is reliant on correct knowledge of the state space, or causal structure, of the learning problem. Indeed, the four learning mechanisms we have described here might be interpreted mathematically as different instantiations of the task state space—only one of them correct—and there are clearly other possible instantiations. In our case, this state space defines which stimuli might lead to which outcomes. Closely related theories of OFC function posit that OFC activity is responsible for inferring and maintaining knowledge of this state space (Takahashi et al., 2011, Wilson et al., 2014). Critically, knowledge of the state space is orthogonal to another common distinction in learning theory, the division between model-based and model-free learning (Daw et al., 2005, Daw et al., 2011, Dayan and Daw, 2008, Dayan and Niv, 2008). Both model-based and model-free learning require a correct knowledge of the state space and correct contingent updating (Wilson et al., 2014). In our experiments, subjects were explicitly informed about the causal structure of the task (even though this information was misleading in experiment 2). Thus, our results speak to the lOFC’s role in leveraging this knowledge of the state space, but not to the issue of how or where in the brain this structure might be learned or inferred from experience. Furthermore, while our task was an instrumental learning task, the role of lOFC in this task likely is in representing stimulus-outcome associations (Schoenbaum et al., 2009), rather than action-outcome associations, which instead appear to rely more on anterior cingulate cortex (Kennerley et al., 2006, Rudebeck et al., 2008, Luk and Wallis, 2013).

We found a parallel but contrasting role for amygdala responses in learning. Suppression of the amygdala occurred before contingent rewards. The absence of this suppression allowed false learning from free rewards and statistical learning to take place. Counterintuitively, however, subjects with strongest amygdala responses to the free rewards were least likely to learn falsely or statistically from these rewards, perhaps because learning from these rewards also required amygdala suppression. Critically, in experiment 2, we had an entire condition where learning was only possible using statistical learning by spreading credit to the average choice. We found that amygdala became exclusively reward responsive in this condition, but not in the conditions where outcomes could be linked to a particular causative choice. The requirement for amygdala suppression to prevent statistical learning may go some way toward explaining why amygdala lesions during reversal learning lead to faster acquisition of the reversals (Rudebeck and Murray, 2008) and why reversal learning deficits following OFC lesions are abolished after subsequent lesions to the basolateral amygdala (Stalnaker et al., 2007).

Hence, activity in lOFC and amygdala was important for correctly assigning credit for contingent rewards and preventing the misassignment of noncontingent rewards. Such activity might be important as there are other brain systems where learning occurs in simpler fashions, not respecting the true causal structure of the reward environment. We found clear examples of such learning in the putamen and associated motor cortex. Here, rewards evoked stronger responses the sooner they were delivered following a choice, and subjects that exhibited this pattern of activity most strongly were most likely to exhibit noncontingent learning patterns, particularly by learning via proximal choices. Furthermore, we found that responses to contingent rewards in a midbrain region consistent with the location of dopaminergic cell bodies were negatively related to contingent learning. Specifically, midbrain responses to contingent reward were associated with a misattribution of these rewards to both proximal choices (PROX) and the average choice history (SoE_Ch_), the exact opposite relationship to that observed in lOFC.

A number of neuronal mechanisms have been suggested to underlie credit assignment via contingent and noncontingent learning. Neurons in OFC carry representations of outcome identity over delay periods (Lara et al., 2009), and they encode the choice made by an animal at the time of outcome delivery (Tsujimoto et al., 2009). This might be a mechanism to link outcomes to their causal choices. Alternatively, neurons in primate dorsolateral prefrontal cortex (dlPFC) carry representations of both the current choice and previous choices (Seo et al., 2007). This might be used by reinforcement learning mechanisms in the basal ganglia to bridge temporal gaps when outcomes are delayed. Noncontingent learning mechanisms likely recruit different mechanisms, of which those underlying PROX are arguably best understood. It has been shown that a dopamine burst will only promote spike-timing-dependent plasticity at striatal dendritic spines if that burst occurs within a narrow time window of 0.3–2 s after the sensorimotor input (Yagishita et al., 2014), which is remarkably consistent with the time window during which PROX occurred in our data. Learning via such eligibility traces (Sutton and Barto, 1998) might also be leveraged for learning using SoE_Ch_ when the broad history of choices is reinforced, rather than a single action (Bogacz et al., 2007). Again, coding of past choices by dlPFC neurons might play a role in such eligibility traces spanning multiple actions.

Taken together, we have shown that in a complex environment, behavior is guided by separable contingent and noncontingent learning mechanisms that compete for control over behavior. The lOFC takes a key position in guiding the balance between these mechanisms. It supports contingent learning by encoding contingent associations between outcomes and their causal choices and suppresses the contribution of noncontingent mechanisms. Amygdala activity following a choice plays a role in noncontingent learning via statistical mechanisms, whereas noncontingent learning via heuristic mechanisms is related to reward responses in motor corticostriatal circuitry and regions of the dopaminergic midbrain.

## Experimental Procedures

Ethical approval for methods and procedures was obtained from the Central University Research Ethics Committee of the University of Oxford.

### Behavioral Analyses Experiment 1

In order to estimate the contribution of different learning mechanisms to behavior, we used a multiple logistic regression that tested the impact of past rewards on future selections of an option, depending on when these rewards occurred relative to choice. We set up the following model:

$$\text{Y}={\text{β}}_{\text{0}}{\text{X}}_{\text{0}}+{\text{β}}_{\text{a}}{\text{X}}_{\text{a}}+{\text{β}}_{\text{b}}{\text{X}}_{\text{b}}+{\text{β}}_{\text{c}}{\text{X}}_{\text{c}}+\text{η,}$$

where Y is the dependent outcome “choice of current option” (0/1); X_0_ is a constant term; and X_a_, X_b_, and X_c_ represent three matrices that each contained 40 regressors (8 × 5) coding for eight past rewards, each split into five bins. Each regressor represented choice of an option in the corresponding time bin (0–0.5, 0.5–1.5, 1.5–2.5, 2.5–3.5, and 3.5–4.5 s prior to reward onset. Matrix X_a_ represented choices of the “same” option A, whereas X_b_ represented choices of “different” options B or C. The shape on the current trial was always designated as A, whereas the other shapes were labeled B and C. Matrix X_c_ was identical to X_b_ but was interacted with the frequency of previous choices of option A during the past 30 shape presentations. This allowed us to assess how credit for a reward following one choice, B or C, was more likely to be misassigned to A as a function of how often A had been selected in the past. The nuisance term η represents three further regressors coding for the frequency of previous A choices, the number of overall choices, and the overall number of rewards observed during the past 30 symbol presentations. These nuisance regressors therefore controlled for simple autocorrelation in choice (1) specific to the particular option and (2) generally regardless of what choice was made and additionally for the effects of the number of rewards earned in the recent past. For subsequent analyses (Figures 2A–2C), we summed the resulting regression coefficients over the eight past rewards for each of the five time bins in X_a_, X_b_, and X_c_.

A separate logistic regression was performed to estimate the effect of the average rate of noncontingent rewards on the average rate of responding, termed SoE_Rew_ in the manuscript (Supplemental Experimental Procedures).

### Acquisition and Analysis of fMRI Data

MRI data were acquired on a 3T Siemens Verio (experiment 1) and on a 3T Siemens Trio (experiment 2, Siemens Germany) system equipped with a 32-channel phased-array head coil as described in detail previously (Jocham et al., 2012). A total of 514 (experiment 1) or 933 (experiment 2) volumes was acquired on average, depending on subjects’ reaction times, thus resulting in total task durations of about 26 and 44 min, respectively. We used Presentation (Neurobehavioral Systems) to present the task and record subjects’ behavior.

Analysis of fMRI data was performed using tools from the Functional Magnetic Resonance Imaging of the Brain (FMRIB) Software Library (FSL; Smith et al., 2004). Functional data were motion corrected using rigid-body registration to the central volume (Jenkinson et al., 2002), corrected for geometric distortions using the field maps and an n-dimensional phase-unwrapping algorithm (Jenkinson, 2003), and high-pass filtered using a Gaussian-weighted lines filter (1/100 Hz and 1/50 Hz for experiments 1 and 2), and spatial smoothing was applied using a Gaussian filter with 6 (experiment 1) and 5 (experiment 2) mm full width at half maximum. EPI images were registered with the high-resolution brain images and normalized into standard (MNI) space using affine registration (Jenkinson and Smith, 2001). A general linear model was fitted into prewhitened data space to account for local autocorrelations (Woolrich et al., 2001).

For experiment 1, we set up a single GLM that contained two regressors that coded for the onsets of contingent and noncontingent rewards, respectively. Another regressor contained the onsets of all rewards, but with the time elapsed since last action as a parametric modulator. The duration was modeled with 0.4 s, corresponding to the actual reward display. Two further regressors were included to model the main effect of stimulus presentation (duration 1.5 s) and response (modeled as stick function). In addition, the six motion parameters from the motion correction were included in the model to account for residual head motion. For experiment 2, we constructed a GLM that contained eight separate regressors that accounted for the four triplets of interest (AAA, AAB, BAB, and BAA), split up by the outcome (reward or nonreward) on the second trial, each aligned to the outcome of the triplet’s second trial. Contrast images from the first level were then taken to the group level using a random effects analysis. Results are reported at p < 0.01, cluster-based correction for multiple comparisons using a cluster-extent threshold of p < 0.05, unless stated otherwise.

## Author Contributions

G.J. designed experiments, acquired data, analyzed data, and wrote the manuscript. K.H.B. designed experiments, acquired data, analyzed data, and wrote the manuscript. A.O.C. acquired data, analyzed data, and wrote the manuscript. M.C.K. and A.M.I. designed experiments and analyzed data. M.E.W. designed experiments and wrote the manuscript. M.F.S.R. designed experiments and wrote the manuscript. T.E.J.B. designed experiments, analyzed data, and wrote the manuscript.

## Supplemental Information

Document S1. Supplemental Experimental Procedures, Figures S1–S6, and Table S1

Document S2. Article plus Supplemental Information

## References

[bib1] Bogacz R., McClure S.M., Li J., Cohen J.D., Montague P.R. (2007). Short-term memory traces for action bias in human reinforcement learning. Brain Res..

[bib2] Charnov E.L. (1976). Optimal foraging, the marginal value theorem. Theor. Popul. Biol..

[bib3] Chudasama Y., Robbins T.W. (2003). Dissociable contributions of the orbitofrontal and infralimbic cortex to pavlovian autoshaping and discrimination reversal learning: further evidence for the functional heterogeneity of the rodent frontal cortex. J. Neurosci..

[bib4] Daw N.D., Niv Y., Dayan P. (2005). Uncertainty-based competition between prefrontal and dorsolateral striatal systems for behavioral control. Nat. Neurosci..

[bib5] Daw N.D., Gershman S.J., Seymour B., Dayan P., Dolan R.J. (2011). Model-based influences on humans’ choices and striatal prediction errors. Neuron.

[bib6] Dayan P., Daw N.D. (2008). Decision theory, reinforcement learning, and the brain. Cogn. Affect. Behav. Neurosci..

[bib7] Dayan P., Niv Y. (2008). Reinforcement learning: the good, the bad and the ugly. Curr. Opin. Neurobiol..

[bib8] Devenport L.D. (1979). Superstitious bar pressing in hippocampal and septal rats. Science.

[bib9] Dias R., Robbins T.W., Roberts A.C. (1996). Dissociation in prefrontal cortex of affective and attentional shifts. Nature.

[bib10] Dias R., Robbins T.W., Roberts A.C. (1997). Dissociable forms of inhibitory control within prefrontal cortex with an analog of the Wisconsin Card Sort Test: restriction to novel situations and independence from “on-line” processing. J. Neurosci..

[bib11] Dickinson A., Watt A., Griffiths W.J.H. (1992). Free-operant acquisition with delayed reinforcement. Q. J. Exp. Psychol..

[bib12] Doll B.B., Hutchison K.E., Frank M.J. (2011). Dopaminergic genes predict individual differences in susceptibility to confirmation bias. J. Neurosci..

[bib13] Fellows L.K. (2007). The role of orbitofrontal cortex in decision making: a component process account. Ann. N Y Acad. Sci..

[bib14] Izquierdo A., Suda R.K., Murray E.A. (2004). Bilateral orbital prefrontal cortex lesions in rhesus monkeys disrupt choices guided by both reward value and reward contingency. J. Neurosci..

[bib15] Jenkinson M. (2003). Fast, automated, N-dimensional phase-unwrapping algorithm. Magn. Reson. Med..

[bib16] Jenkinson M., Smith S. (2001). A global optimisation method for robust affine registration of brain images. Med. Image Anal..

[bib17] Jenkinson M., Bannister P., Brady M., Smith S. (2002). Improved optimization for the robust and accurate linear registration and motion correction of brain images. Neuroimage.

[bib18] Jocham G., Hunt L.T., Near J., Behrens T.E. (2012). A mechanism for value-guided choice based on the excitation-inhibition balance in prefrontal cortex. Nat. Neurosci..

[bib19] Jones B., Mishkin M. (1972). Limbic lesions and the problem of stimulus--reinforcement associations. Exp. Neurol..

[bib20] Kamin L.J. (1961). Trace conditioning of the conditioned emotional response. J. Comp. Physiol. Psychol..

[bib21] Kennerley S.W., Walton M.E., Behrens T.E., Buckley M.J., Rushworth M.F. (2006). Optimal decision making and the anterior cingulate cortex. Nat. Neurosci..

[bib22] Kringelbach M.L., Rolls E.T. (2004). The functional neuroanatomy of the human orbitofrontal cortex: evidence from neuroimaging and neuropsychology. Prog. Neurobiol..

[bib23] Lara A.H., Kennerley S.W., Wallis J.D. (2009). Encoding of gustatory working memory by orbitofrontal neurons. J. Neurosci..

[bib24] Luk C.H., Wallis J.D. (2013). Choice coding in frontal cortex during stimulus-guided or action-guided decision-making. J. Neurosci..

[bib25] Mishkin M., Warren J.M., Akert K. (1964). Perseveration of central sets after frontal lesions in monkeys. The Frontal Granular Cortex and Behavior.

[bib26] Morrison S.E., Salzman C.D. (2009). The convergence of information about rewarding and aversive stimuli in single neurons. J. Neurosci..

[bib27] Padoa-Schioppa C., Assad J.A. (2008). The representation of economic value in the orbitofrontal cortex is invariant for changes of menu. Nat. Neurosci..

[bib28] Redgrave P., Gurney K. (2006). The short-latency dopamine signal: a role in discovering novel actions?. Nat. Rev. Neurosci..

[bib29] Rudebeck P.H., Murray E.A. (2008). Amygdala and orbitofrontal cortex lesions differentially influence choices during object reversal learning. J. Neurosci..

[bib30] Rudebeck P.H., Behrens T.E., Kennerley S.W., Baxter M.G., Buckley M.J., Walton M.E., Rushworth M.F. (2008). Frontal cortex subregions play distinct roles in choices between actions and stimuli. J. Neurosci..

[bib31] Schoenbaum G., Chiba A.A., Gallagher M. (1998). Orbitofrontal cortex and basolateral amygdala encode expected outcomes during learning. Nat. Neurosci..

[bib32] Schoenbaum G., Roesch M.R., Stalnaker T.A., Takahashi Y.K. (2009). A new perspective on the role of the orbitofrontal cortex in adaptive behaviour. Nat. Rev. Neurosci..

[bib33] Selemon L.D., Goldman-Rakic P.S. (1985). Longitudinal topography and interdigitation of corticostriatal projections in the rhesus monkey. J. Neurosci..

[bib34] Seo H., Barraclough D.J., Lee D. (2007). Dynamic signals related to choices and outcomes in the dorsolateral prefrontal cortex. Cereb. Cortex.

[bib35] Skinner B.F. (1948). Superstition in the pigeon. J. Exp. Psychol..

[bib36] Smith S.M., Jenkinson M., Woolrich M.W., Beckmann C.F., Behrens T.E., Johansen-Berg H., Bannister P.R., De Luca M., Drobnjak I., Flitney D.E. (2004). Advances in functional and structural MR image analysis and implementation as FSL. Neuroimage.

[bib37] Stalnaker T.A., Franz T.M., Singh T., Schoenbaum G. (2007). Basolateral amygdala lesions abolish orbitofrontal-dependent reversal impairments. Neuron.

[bib38] Sutton R.S., Barto A.G. (1998). Reinforcement learning: an introduction.

[bib39] Takahashi Y.K., Roesch M.R., Wilson R.C., Toreson K., O’Donnell P., Niv Y., Schoenbaum G. (2011). Expectancy-related changes in firing of dopamine neurons depend on orbitofrontal cortex. Nat. Neurosci..

[bib40] Thorndike E.L. (1933). A proof of the law of effect. Science.

[bib41] Thorpe S.J., Rolls E.T., Maddison S. (1983). The orbitofrontal cortex: neuronal activity in the behaving monkey. Exp. Brain Res..

[bib42] Tremblay L., Schultz W. (1999). Relative reward preference in primate orbitofrontal cortex. Nature.

[bib43] Tsujimoto S., Genovesio A., Wise S.P. (2009). Monkey orbitofrontal cortex encodes response choices near feedback time. J. Neurosci..

[bib44] Walton M.E., Behrens T.E., Buckley M.J., Rudebeck P.H., Rushworth M.F. (2010). Separable learning systems in the macaque brain and the role of orbitofrontal cortex in contingent learning. Neuron.

[bib45] Wilson R.C., Takahashi Y.K., Schoenbaum G., Niv Y. (2014). Orbitofrontal cortex as a cognitive map of task space. Neuron.

[bib46] Woolrich M.W., Ripley B.D., Brady M., Smith S.M. (2001). Temporal autocorrelation in univariate linear modeling of FMRI data. Neuroimage.

[bib47] Yagishita S., Hayashi-Takagi A., Ellis-Davies G.C., Urakubo H., Ishii S., Kasai H. (2014). A critical time window for dopamine actions on the structural plasticity of dendritic spines. Science.

[bib48] Yin H.H., Knowlton B.J. (2006). The role of the basal ganglia in habit formation. Nat. Rev. Neurosci..

