# Supplementary material for: Reward-Guided Learning with and without Causal Attribution
Source: Neuron. 2016 Apr 6;90(1):177–90. doi: 10.1016/j.neuron.2016.02.018 (PMC4826429; doi:10.1016/j.neuron.2016.02.018)
Supplement: Document S1. Supplemental Experimental Procedures, Figures S1–S6, and Table S1 [file mmc1.pdf]

Neuron, Volume *90*

Supplemental Information

Reward-Guided Learning with

and without Causal Attribution

Gerhard Jocham, Kay H. Brodersen, Alexandra O. Constantinescu, Martin C. Kahn, Angela M. Ianni, Mark E. Walton, Matthew F.S. Rushworth, and Timothy E.J. Behrens

SUPPLEMENTAL DATA

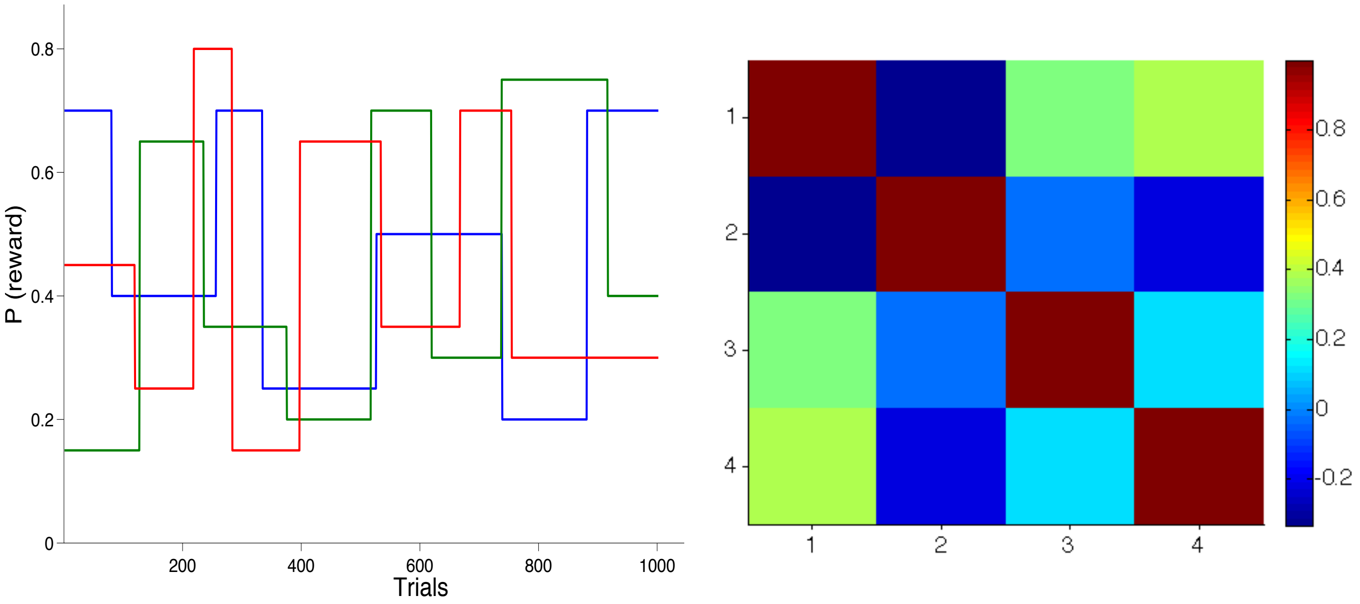

Figure S1 (related to main figure 2). Left: Underlying reward probabilities for the three options in experiment 1. Right: correlation matrix for the behavioural effects in experiment 1 (1 = contingent learning, 2 = PROX, 3 = SoE_{Ch}, 4 = SoE_{Rew}). Subjects responded to 467 ± 21.8 (mean ±SEM) of the 1002 shape presentations and earned 237 ± 9 contingent rewards and 240 ± 6 non-contingent rewards. The number of contingent rewards earned did not differ from the number of non-contingent rewards ($p = 0.81$). Subjects allocated their choices primarily to the best option, where "best" option refers to the option with the highest reward probability of the underlying reward schedule. Even under this conservative performance criterion (the underlying probabilistic reward structure is unknown to participants and due to the frequent reversals several trials are required following each change to obtain a reliable estimate of the options' values) subjects allocated 49% (± 1.48) of their choices to the currently best option, 28% (± 0.61) to the second best, and the remaining 23% (± 1.07) to the third best option. Thus, while far from ceiling, subjects clearly were able to perform the task: selection of the best option was higher than chance (33%) level ($t_{29} = 10.64$, $p < 0.0000000001$) and subjects allocated a higher percentage of their choices to the best compared to the second best option ($t_{29} = 10.22$, $p < 0.0000000001$) and in turn a higher percentage to the second compared to the third best option($t_{29} = 5.96$, $p < 0.00001$).

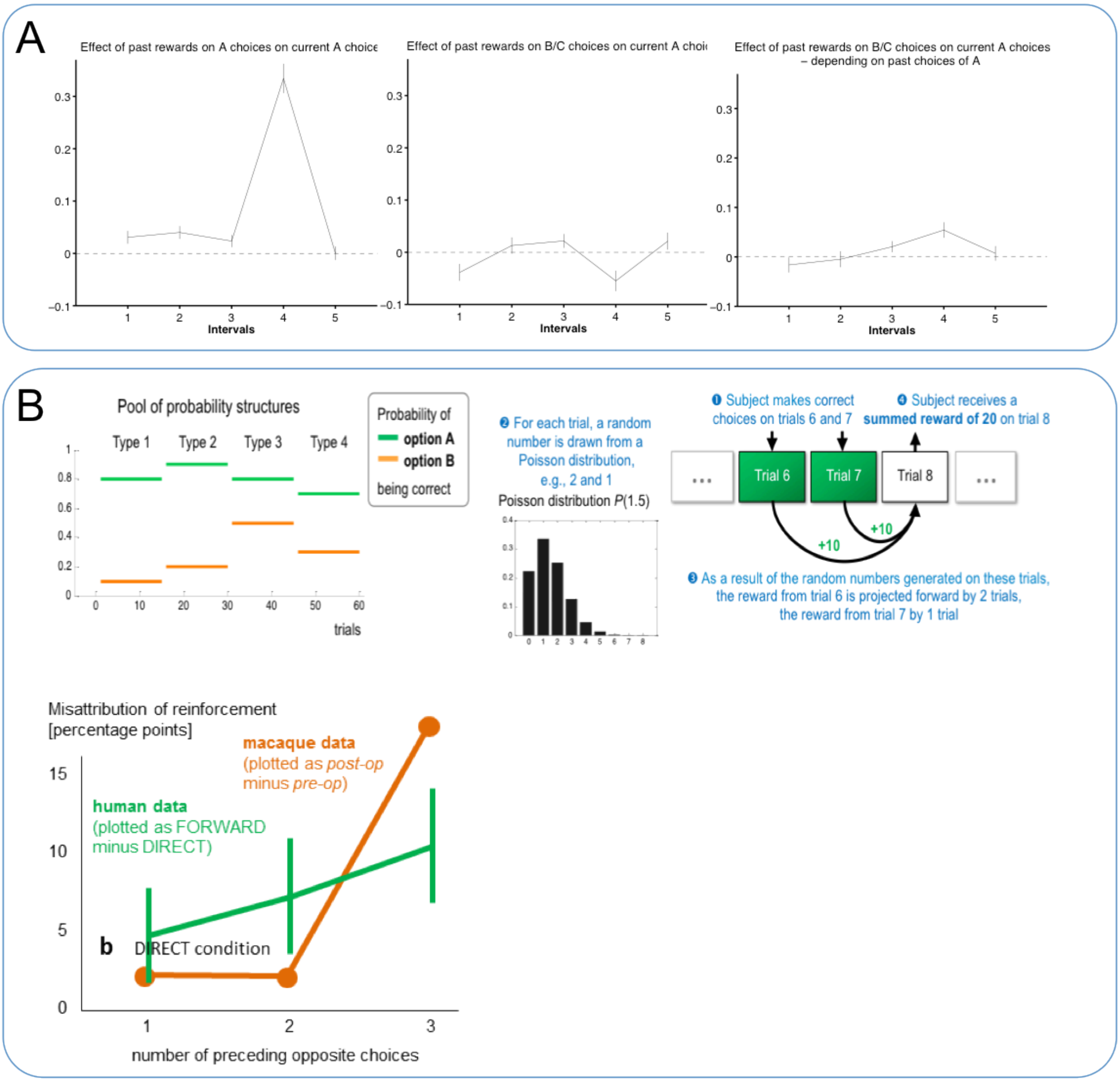

Figure S2 (related to main figure 2). A) Same analysis as reported in main figure 2 A–C replicated without the seven pilot subjects (Considering only the subjects that performed the fMRI experiment, n = 23). Again, rewards depended on the time bin in which they fell (ANOVA, effect of bin, $F_{4,88}$ = 71.6, p < 0.0001). Rewards had a particularly strong influence on the later selection of the choice made 3 seconds prior to the reward (t_{22} = 11.94 p < 0.0001, bin 4). Likewise, rewards that occurred in the early bins 1 and 2 (t_{22} = 2.12 and t_{22} = 3.38, p < 0.02) and even in the intermediate bin 3 (t_{22} = 2.12, p = 0.046) increased the likelihood of repeating these same choices. Rewards in late bin 5 had no effect on behaviour (p > 0.98). Furthermore, the effects in the early bins 1 (t_{22} = 2.05, p = 0.026, one-tailed) and bin 2 (t_{22} = 2.35, p = 0.014, one-tailed) were bigger compared to late bin 5. Contingent rewards following *B or C* choices increased future *A* choices as an increasing function of the frequency of A choices in the past 30 trials (ANOVA effect of bin $F_{4,88}$ = 3.29, p = 0.0146, t-test for bin 4: t_{22} = 3.58, p = 0.0017). The separate regression testing the effects of the time-averaged rate of contingent and noc-contingent rewards on the time-averaged rate of responding showed that the rate of responding strongly depended on the rate of non-contingent rewards (t_{22} = 5.28, p < 0.00005). The number of contingent rewards obtained (247 ± 10) did not differ from the number of non-contingent rewards (226 ± 4.33, p > 0.14). Subjects allocated 48% (± 1.64) of their choices to the currently best option, 28% (± 0.73) to the second best, and the remaining 24% (± 1.16) to the third best option. Selection of the best option was higher than chance (33%) level (t_{22} = 9.15, p < 0.00000001) and subjects allocated a higher percentage of their choices to the best compared to the second best option (t_{22} = 8.68, p < 0.0000001) and in turn a higher percentage to the second compared to the third best option(t_{29} = 4.7, p < 0.0002).

B) Top: Block design, probability structure and forward projection of rewards in experiment 2. Bottom: Learning strategies under DIRECT and FORWARD instructions. We used the measure described in the section "validation of instruction manipulation" to delineate the two hypothesized ways of forming associations. When adopting normal contingency learning (as intended in DIRECT blocks), subjects should associate any credit or blame they received after the B choice with B itself; this means that the above measure would be negative. By contrast, when adopting temporal contingency learning (as intended in FORWARD blocks), subjects should associate any credit or blame after their B choice with the history of previous choices, that is, with A; the above measure would be positive. Particularly strong evidence for temporal association learning would be provided by a positive correlation between the length of the history of A choices and the above measure. It would be strongly suggested that a subject forms indirect associations if it was observed that their likelihood of returning to A (staying away from A) increased with a growing number of A choices before a single rewarded (unrewarded) B choice. This is exactly what we observed (bottom). All error bars represent SEM.

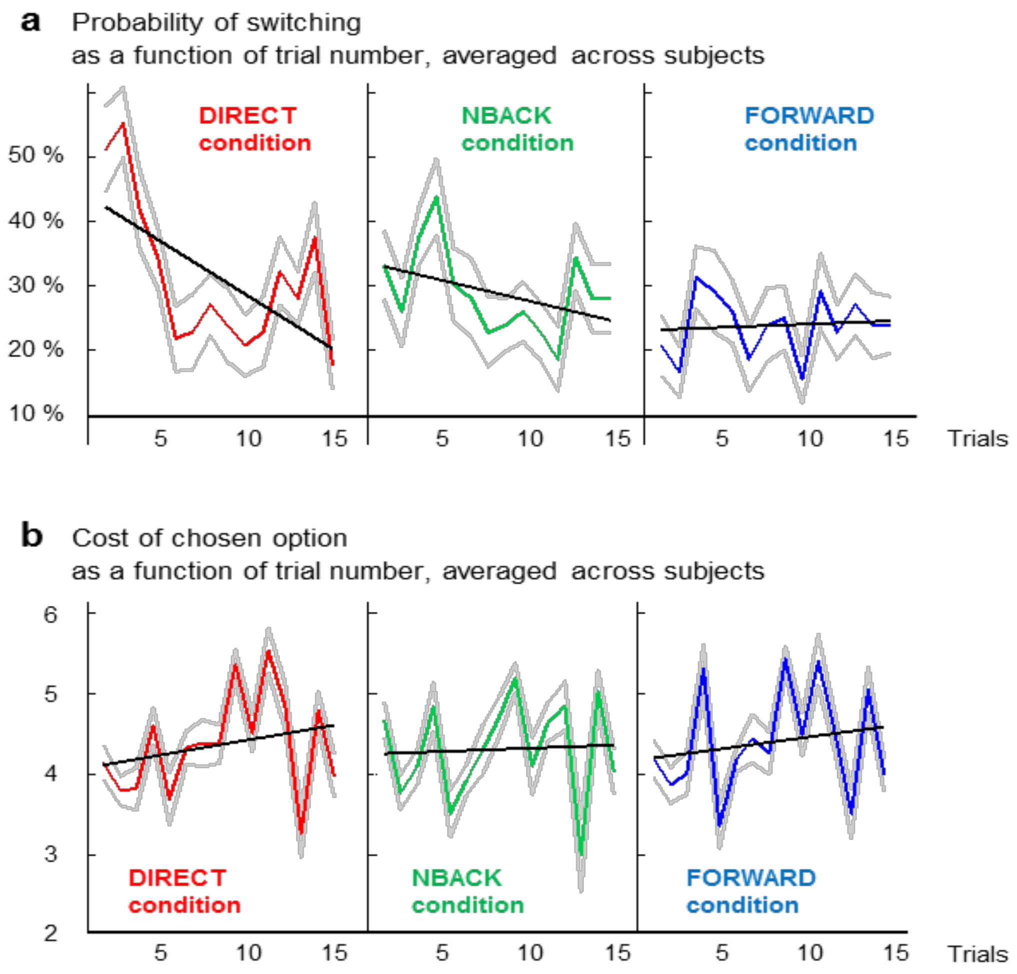

Figure S3 (related to main figure 2). Validation of switching behaviour and costs. Subjects were expected to adopt different policies in different blocks. We tested three hypotheses about how likely subjects should be to switch options *within* a block of trials. In DIRECT blocks, subjects should initially sample the two options freely, potentially attracted towards low costs. Then, towards the end of a block, they were expected to have learned which option had a higher reward probability, and should consequently switch much less frequently. In FORWARD blocks, in which the outcome of an option could only be judged on a longer temporal scale, subjects were expected to stick with an option for a longer time. Their switching probability should be lower than in DIRECT blocks. In NBACK blocks, finally, subjects were expected to display some intermediate switching behaviour in between DIRECT and FORWARD blocks. To test these hypotheses, we averaged switching likelihoods for each trial within a block (Figure S3A). The figure shows the relative number of blocks in which subjects switched from option A to option B, or vice versa, on a given trial. Values are plotted as mean (coloured) +/– one standard error (grey). Only in the case of DIRECT instructions is the slope of a linear least-squares regression through the data (black) significantly different from zero ($p_D < 0.001$, $p_F = 0.73$, $p_M = 0.12$). The diagrams are based on data obtained during fMRI scanning (n = 24). These data allowed us to confirm all three hypotheses. In particular, DIRECT blocks, but not FORWARD or NBACK blocks, showed a significant decline in switching likelihood over the course of a block. In addition, an intriguing effect was observed in the first few trials. In DIRECT blocks, a strong initial peak was followed by a subsequent fall of the average switching likelihood. This pattern reoccurred precisely, albeit in a slightly weaker form, in NBACK blocks – with a delay of about 2 trials (Figure S3A).

Care was taken in the design of the experiment to control for random variations in costs: the same randomly generated sequence of costs was used exactly once within each type of block. In the fMRI variant, for example, with its total number of twelve blocks, only four different sequences of cost pairs were generated for each subject. Therefore, if the costs of both cards offered on a particular trial happened to be very high, this should directly show up in the averaged chosen cost on that trial, across all instructions (Figure S3B). To test this, the figure shows the cost of the chosen card on each trial, averaged for DIRECT, NBACK, and FORWARD blocks, respectively. Since the same randomly generated sequences of costs were used in all block types, the same pattern of peaks and troughs shows up in all subplots, so that

differences between the blocks are solely due to subtle variations in subjects' decision policies. An additional test was made about the evolution of costs during a block, examining the following hypothesis: the easier it is for subjects to learn, the more quickly should they be prepared to choose expensive options which they believe lead to a reward that justifies the cost. The hypothesis was confirmed: DIRECT blocks were the only blocks in which the slope of a linear least-squares regression was significantly greater than zero ($p < 0.05$, figure S3B).

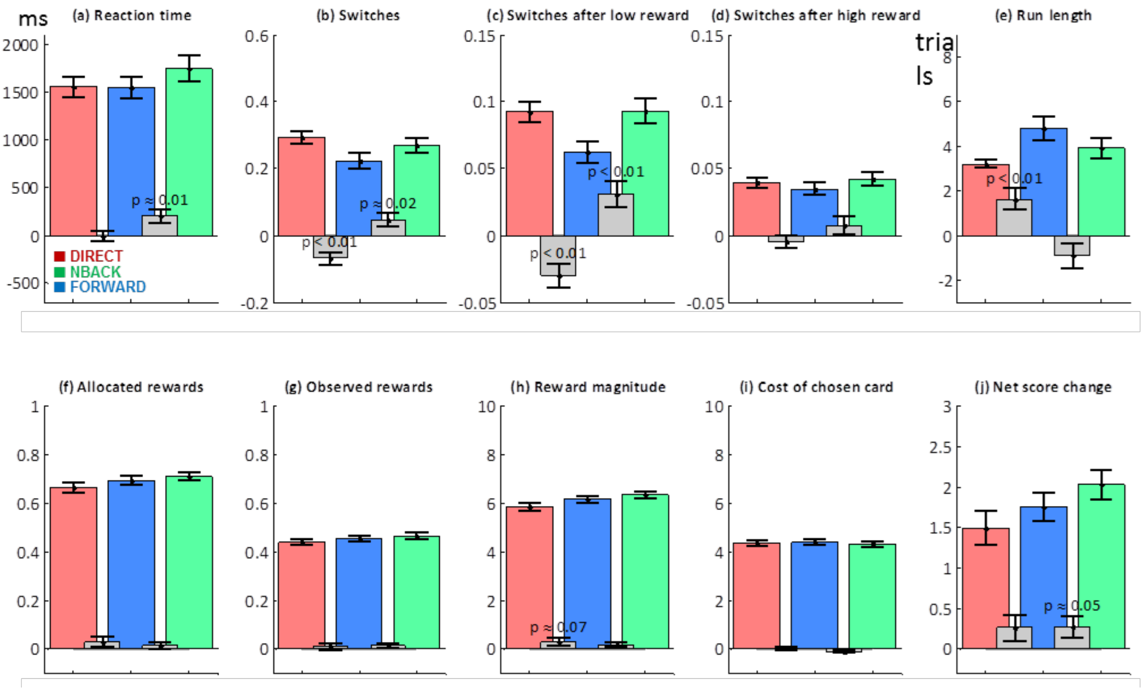

Figure S4 (related to main figure 2). Validation of additional behavioural indices. All experimental blocks were based on the same principle of forward projection of rewards, whereas instructions differed from block to block. As shown above, instructions predicted the form of association learning induced in human subjects. However, in order to allow for meaningful interpretation of any fMRI results, the paradigm had to control for several additional behavioural characteristics that might otherwise introduce confounds in the measured haemodynamic response. Each diagram shows how a particular behavioural descriptor differs between blocks of different instruction types. All values are given as mean +/– one standard error and, unless indicated otherwise on the y-axis, were calculated as `average per-trial' quantities. The three coloured bars represent DIRECT blocks (red), FORWARD blocks (blue), and NBACK blocks (green). The grey bars represent the averaged paired differences between DIRECT and FORWARD (first grey bar) and between FORWARD and NBACK blocks (second grey bar). Wherever a difference was significantly different from zero (with respect to $\alpha=0.1$), the p-value of a two-tailed t-test is given above the respective bar. The diagrams are based on behavioural data obtained during fMRI scanning (n=24).

(a) Reaction times. The average reaction time was calculated as the mean per-trial time between cue and response, that is, between the display of the two options and the subject making a decision. Subjects took longest to respond in NBACK blocks ($p = 0.01$). Increased response times in these blocks could be considered a result of more time required to access working memory and associate the outcome of the previous trial with the choice that led to it. By contrast, no significant difference in reaction times was found between DIRECT and FORWARD blocks, providing a strong control for the fMRI experiment.

(b, e) Switches and run lengths. The average number of switches per trial represents the subjects' mean likelihood of switching from one option to the other on any given trial. It is inversely proportional to the run length, the number of trials for which subjects would, on average, stick with the same option. In order to find out about the reward probabilities of the two options, subjects were expected to sample the same option for longer consecutive periods of time in FORWARD blocks than in DIRECT blocks, in which subjects were expected to choose among the two options in a more volatile way. This is exactly what was found: the average switching likelihood was highest in DIRECT blocks and lowest in FORWARD blocks; equivalently, the mean run length was found to be shortest in DIRECT blocks and longest in FORWARD blocks, differing by approximately two trials. This shows that the paradigm significantly influenced subjects' behaviour but, at the same time, provided a one-way control for the fMRI experiment: should a brain area be found to be more active in FORWARD blocks than in DIRECT blocks, then this finding could not be explained away by a higher frequency of behavioural switches (Crone et al., 2006).

(c, d) Switches after low/high reward. An unexpected result was obtained in the dependency of switches on reward magnitudes. In DIRECT and NBACK blocks, subjects had been instructed only to take into account whether a particular option was rewarded, but not to be distracted by the reward magnitude - an ostensibly 'random' number $r \in \{10, 20, 30,..\}$. However, splitting up trials into those following a low reward ($r = 10$) and those following a high reward ($r > 10$) revealed that subjects, perhaps unconsciously, did take into account reward magnitudes: they were more likely to switch away from their current option after a low reward than after a high reward. This effect is easiest to interpret in DIRECT blocks, in which a switch represents a rejection of the last option.

(f, g, h) Rewards. The experimental paradigm was designed to lead to similar reward levels under DIRECT and FORWARD conditions. Here, this notion was once more confirmed by looking at three more detailed descriptors of reward. In these diagrams, the number of allocated rewards describes how often a rewarded card was chosen, whereas the number of observed rewards describes how often a reward was actually displayed on the screen. This number necessarily had to be smaller than the number of allocated rewards: rewards were allowed to pile up on the same trial, and were sometimes projected forward beyond the end of a block. Reassuringly, no significant differences were found between different instructions in allocated rewards, observed rewards, or (observed) reward magnitudes. This finding provided a strong control for the fMRI experiment: differences in activity between the different instructions would not be explicable in terms of differences in rewards.

(i) Costs. An ever more stringent test of the validity of the experimental paradigm was made by looking at the costs of the chosen cards, which were, unlike rewards, under the subjects' direct control. As before, no significant differences between the blocks were found. In particular, no type of instruction led subjects to ignore reward probabilities and behave in a mere cost-minimizing fashion. Both findings provided strong controls for the fMRI experiment.

(j) Net score changes. The difference, on each trial, between the observed reward and the cost yielded the net score change. Although slightly more frequent observed rewards, slightly higher reward magnitudes, and slightly lower average costs of the chosen card led to a net score change in NBACK blocks that was higher than in both other types of block ($p < 0.05$), no significant difference was found between DIRECT and FORWARD blocks, providing a strong control for fMRI.

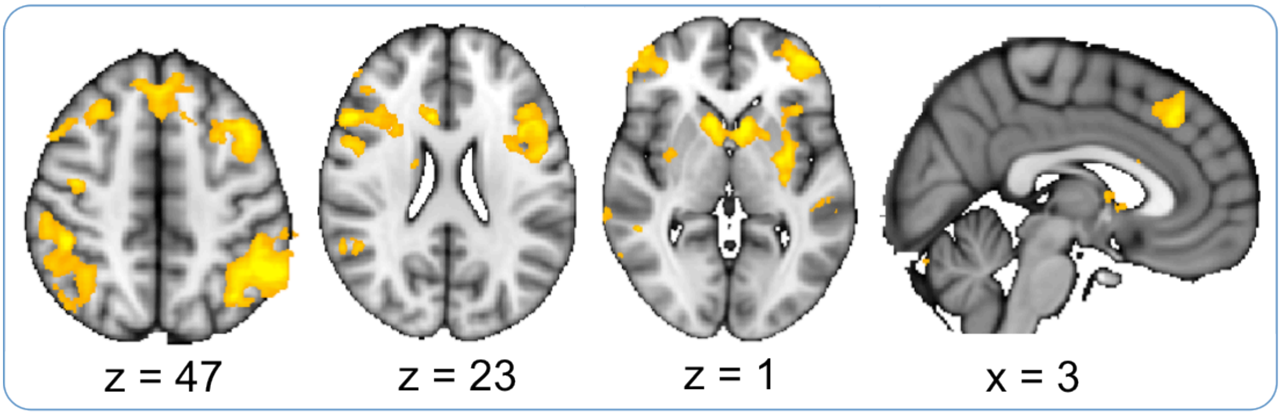

Figure S5 (related to main figure 3). Additional areas found active in the main contrast contingent – non-contingent rewards in experiment 1.

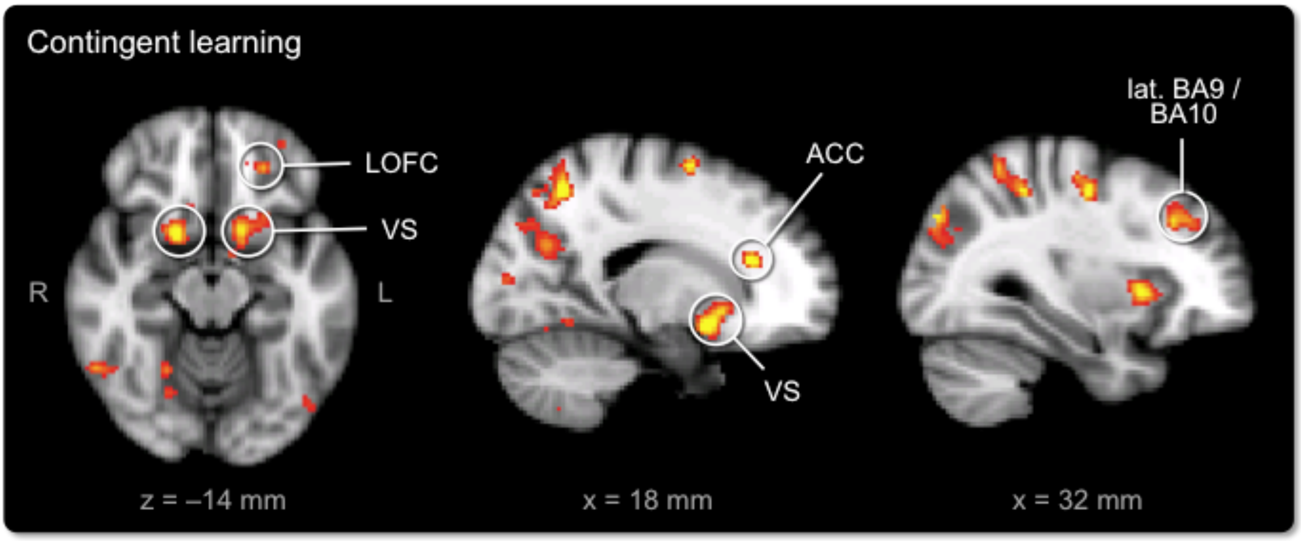

Figure S6 (related to main figure 4). Whole-brain contingency effects (experiment 2). Several areas were found to reflect, at the time of observing the outcome of a decision, whether a correct choice-outcome-update contingency is being applied ($z > 3.1$). Of particular interest in this result are the ventral striatum (VS), lateral orbitofrontal cortex (OFC), anterior cingulate cortex (ACC), and the border between lateral Brodmann areas (BA) 9 and 10.

Table S1 (related to main figure 3). List of peak activation for clusters found in the main contrast [contingent – non-contingent rewards in experiment 1. This table provides a full list of cluster maxima for this contrast. Please note however that despite the conservative thresholding approach ($p < 0.001$ activation threshold, cluster-based threshold at $p < 0.05$), many of the activations found here are rather large and span more than a single brain area. For this reason, the lables we assigned to these clusters should be taken with care. We additionally display several views (figure S5) to give a more comprehensive picture of the pattern of brain activity found here.

Region	MNI coordinates (xyz)	peak Z-score	cluster size (mm3)
Lateral prefrontal cortex	-33 8 20	5.24	54563
Posterior parietal cortex	-51 -51 44	5.4	14510
Posterior parietal cortex	45 -38 49	4.85	8352
Inferior/middle temporal gyrus	-58 -36 -12	4.96	6502
Lateral orbitofrontal cortex	-24 35 -12	5.1	5192
Inferior/middle temporal gyrus	63 -39 -6	5.13	3802
Cerebellum	33 -64 -32	4.77	2360
Posterior superior temporal sulcus	-56 -52 17	4.46	1365
Visual cortex (V2/V3/V4)	-20 -87 -11	5.22	1325
Primary motor cortex	41 -9 48	4.4	1030
Temperoparietal junction	57 -50 23	3.77	811

SUPPLMENTAL EXPERIMENTAL PROCEDURES

Experiment 1

Behavioural task

Subjects observed geometrical shapes (circle, square, triangle) moving across the screen from left to right in random succession. Every shape appeared from underneath a "curtain" (a grey rectangle on the left side) and moved rightwards during a period of 1.5 s until it disappeared under another "curtain" on the right-hand side. Subjects could choose to either select the shape present on the screen or ignore it. A shape could be chosen by pressing its associated button (index, middle, and ring finger) with the right hand. Shapes were associated with a probability of reward, when selected, that was independent between options (shapes) and changed several times during the experiment (Figure S1). If a shape was chosen, a response cost equivalent to 1/3 point was deducted which was visualised to subjects by shrinking of the progress bar at the bottom of the screen. This manipulation was implemented to discourage subjects from responding to every option. If a reward was earned for a choice, the equivalent of 1 point was added to the progress bar. The subjects' aim was to hit the gold target line to the lower right of the screen to win £2, after which the progress bar was reset to zero and subjects started afresh. Importantly, if a subject's choice resulted in a reward, this reward was delayed by a fixed interval of 3 s. This meant that whenever participants observed a reward, they had to link this with the option chosen 3 s before, not with the shape currently onscreen. In addition to these contingent rewards,

there was also a fixed probability $p = 0.3$ during every symbol presentation that a non-contingent reward could be delivered. If such a non-contingent reward was scheduled, it was present with random timing during the shape presentation. Both types of rewards were presented for 400 ms and consisted of a picture of Scrooge McDuck diving in gold. To make contingent and non-contingent rewards visually distinguishable, they were presented in different colours. For half the participants, contingent rewards were in blue and non-contingent rewards were in red, and the reverse was true for the other half of volunteers. Subjects were explicitly instructed about all these task features. Before they entered the scanner, they first performed a time estimation task where they learnt to estimate a 3 s interval. Next, they learnt the mapping of response fingers to shapes. Following this, they performed a training version of the task that included only the contingent rewards. Thereafter, they performed a version of the task that was identical to the experimental task, including contingent and non-contingent rewards, only with a simpler probabilistic structure. The experimental task consisted of 1002 shape presentations and lasted approximately 25 minutes.

Linear regession testing the effect of average reward rate on average rate of responding

To test whether subjects' average rate of responding depended on the average rate of rewards, we set up the following linear model:

$$Y = \beta_0 X_0 + \beta_1 X_{NCR} + \beta_2 X_{CR}$$

where the dependent variable Y is response on each trial obtained by smoothing with a Gaussian window of 30 trials, X_0 is a constant, and X_{NCR} and X_{CR} represent the rate of non-contingent and contingent rewards, respectively, again obtained by smoothing with a Gaussian window of 30 trials.

Task-related functional connectivity (PPI) analyses between ventral striatum and OFC

Raw BOLD signal timecourses were extracted from the ventromedial striatum (VMS) at the peak coordinate identified in the main contrast of contingent – non-contingent rewards in experiment 1 (see main text). Timecourses were extracted from a maks that contained the peak coordinates in both hemispheres. A general linear model was set up that was identical to that described in the main text for experiment 1, but additionally contained the (demeaned) VMS BOLD timecourse and two PPI regressors that were generated by interacting the contingent and non-contingent reward regressors, respectively, with the VMS timecourse. Differential connectivity during contingent versus non-contingent rewards was then assessed by directly contrasting the two PPI regressors.

Experiment 2

Behavioural task

To test competing hypotheses about the neural mechanisms underlying contingency learning, we designed a novel probabilistic sequential decision-making task. In this task, participants had to learn, by trial and error, the reward probabilities of two options. While undergoing fMRI scanning, each subject completed 12 blocks of our learning task with 15 trials each. In every block, two previously unseen fractals had different probabilities of being associated with a reward; these probabilities varied between blocks. On each trial, participants were asked to choose between two alternative options, represented by two fractal stimulus patterns (Figure 1, right). Unknown to participants, the two

options had different probabilities of leading to a reward. For example, if one of the options had a reward probability of 80%, then that card would, on average, be rewarded on 8 out of 10 trials. The reward probabilities of the two options were mutually independent. Thus, on any given trial, neither card, one card, or both cards could be rewarded. Probabilities varied throughout the experiment, inducing the requirement for continuous learning (Figure S2B).

To encourage subjects to switch options above and beyond normal exploration behaviour (Macready and Wolpert, 1998), the two cards were associated with costs randomly sampled from the interval [1....9]. Subjects began the experiment with an initial baseline score. When choosing a card, its associated cost would be deducted from this score. If the card was rewarded, the score would then be increased by the magnitude of the reward. The overall score subjects had reached by the end of the experiment was directly translated into monetary reimbursement. The task comprised three conditions. In the DIRECT condition, subjects were instructed that, whenever a rewarded option had been chosen, its reward was presented on the same trial. In the NBACK condition, subjects were told that rewards were projected forward by a known number of trials (one or two) such that any given outcome would have to be linked to a previous choice. In the FORWARD condition, finally, subjects were instructed that rewards were delayed by a random number of trials such that outcomes could no longer be linked to their underlying causative decisions (Figure 1, right). In other words, in FORWARD, subjects only know that one of four choices is likely responsible (forward projection of rewards by more than three trials is very unlikely, see supplementary figure S2B). While subjects in FORWARD thus do not know which action caused a particular reward, they can nevertheless learn by assigning credit to the average choice. Unknown to subjects, all three conditions (DIRECT, NBACK, and FORWARD) followed the FORWARD scheme. Thus, across all conditions, rewards were not delivered immediately; instead, they were delayed, or projected forward, by a small random number of trials. This number was, on each trial, drawn from a Poisson distribution P(1.5). The number of trials by which a reward was projected forward could be any non-negative integer, with an average of 1.5 trials. Individual rewards were worth 10 points. If two rewards happened to be projected forward to the same future trial, their values were summed (Figure S2B). This design ensured a delayed and interleaved order of reinforcements, preventing subjects from being able to benefit from forging direct associations (Figure S2B). Since the underlying structure was identical throughout (forward projection and summation of rewards), subjects would inevitably observe varying reward magnitudes in all blocks (10, 20, 30, …). In DIRECT and NBACK blocks, these were explained to them as random multiples of 10 generated on each rewarded trial. Only in FORWARD blocks were subjects accurately told that rewards greater than 10 were a result of the summation of multiple rewards that happened to be projected forward onto the same trial. The fMRI experiment consisted of 12 blocks with varying instructions. Each block contained 15 trials only. Their probabilities followed one out of four predefined probability schemes. Since left/right positions of the two options were randomized within and across subjects, probabilities were decorrelated both from the side of presentation and from the fractal patterns representing the two options.

Validation of instruction manipulation

For the purpose of the behavioural validation of our paradigm, we acquired an initial dataset from 12 healthy volunteers (8 male, 4 female), aged between 22 and 36. This initial validation experiment consisted of one DIRECT block, one NBACK block, and one FORWARD block, each containing 350 trials. The reward probabilities of the two options followed a scheme that was identical for all three blocks. It was designed to contain stretches of fixed, drifting, and reversing probabilities. As shown by this initial behavioural pilot, our instruction manipulation did not only robustly induce the different behaviours that one would expect under the three different instruction types. Critically, this design also provided a number of strong controls for the subsequent imaging analysis. Most importantly, there was no significant difference in reward levels across conditions.

The principal aim of the experimental paradigm was to induce different forms of contingency learning in human subjects. Specifically, FORWARD instructions, compared to DIRECT instructions, were to replicate the behavioural effect observed in OFC-lesioned monkeys . This had led to two specific questions. First, did DIRECT blocks make subjects form associations between choices and their immediate outcomes? Second, did FORWARD blocks lead subjects to associate a reinforcement outcome with the recent history of choices?

In order to answer these questions, a measure for the likelihood of choosing A after a series of A choices and a single B choice was calculated as

$$\frac{\#(\overbrace{\mathrm{A}\cdots\mathrm{A}}^{k}\mathrm{B}^{+}\mathrm{A})}{\#(\underbrace{\mathrm{A}\cdots\mathrm{A}}_{k}\mathrm{B}^{+})} - \frac{\#(\overbrace{\mathrm{A}\cdots\mathrm{A}}^{k}\mathrm{B}^{-}\mathrm{A})}{\#(\underbrace{\mathrm{A}\cdots\mathrm{A}}_{k}\mathrm{B}^{-})} \quad \forall k = 1,2,3,\ldots,$$

where B^+ denotes a rewarded, and B^- an unrewarded, choice of option B. The term $\#(\mathrm{AAB}^{+}\mathrm{A})$, for instance, denotes the number of times the sequence $\mathrm{AAB}^{+}\mathrm{A}$ was found in the entire sequence of choices of a subject. It represents all instances when a subject returned to option A after two consecutive A choices and a single rewarded B choice.

Assessment of how different instructions induce different forms of learning

Despite the true contingencies being identical in each block (FORWARD), we hoped that different instructed contingencies would induce different behaviours. In order to examine whether this was the case, we constructed a simple logistic regression model to explain each subject's choices in terms of their previous choices and rewards. If subjects were indeed learning according to our instructed contingencies, credit for a reward should be assigned: to the immediately preceding choice in the DIRECT condition; to the previous choice in the 1BACK condition; to the choice before the previous choice in the 2BACK condition; and credit should be distributed across the different choices in the FORWARD condition. Hence, in order to explain each choice, we included 4 regressors that indicated the previous 4 choices, and 3 sets of regressors that interacted these choices with the occurrence of rewards: at the current trial; at the following trial; and at the following-but-one trial. These regressors can be arranged into the lower quadrant of a square (Main figure 2E) where the lead diagonal represents DIRECT learning (red), the next lower diagonal represents 1BACK learning (green), and the third diagonal 2BACK learning (yellow).

When estimating this model, we found that a different set of regressors received loading in each instructed condition, despite the true contingencies being identical across all blocks. Specifically, when subjects were instructed that contingencies were DIRECT, the DIRECT regressors were loaded; when 1BACK and 2BACK instructions were given, 1BACK and 2BACK regressors were loaded, respectively; and when subjects were instructed that rewards would be projected FORWARD by a random number of trials, all three sets of regressors were loaded. This statistical interaction between instruction and regressor type was then assessed by a 2-way ANOVA on regressor loadings.

Selection of independent OFC ROI

Our design allowed us to compute contrast from one set of trials and use the peak location to extract data from a different set of trials, thus avoiding any selection bias. More specifically, the data used for the BA-contrasts shown in main figure 4 A, B, C and E are derived from a contrast obtained from using AA trials only. Conversely, the data used for the AA contrasts depicted in main figure 4D and F are derived from the peak coordinate of the contrast obtained from using BA trials only.

SUPPLMENTAL REFERENCES

Crone EA, Wendelken C, Donohue SE, Bunge SA (2006) Neural evidence for dissociable components of task-switching. Cereb Cortex 16:475-486.

Macready WG, Wolpert DH (1998) Bandit Problems and the Exploration/Exploitation Tradeoff. IEEE Transactions on Evolutionary Computations 2:2-22.
