# Supplementary material for: Reward-Guided Learning with and without Causal Attribution
Source: Neuron. 2016 Apr 6;90(1):177–90. doi: 10.1016/j.neuron.2016.02.018 (PMC4826429; doi:10.1016/j.neuron.2016.02.018)
Supplement: Document S2. Article plus Supplemental Information [file mmc2.pdf]

Neuron

Article

Reward-Guided Learning with and without Causal Attribution

Highlights

- Reward-guided learning is driven by several mechanisms operating in parallel
- These mechanisms either respect or only approximate the causal structure of the world
- Learning mechanisms respecting causal structure rely on orbitofrontal cortex
- Other regions mediate learning based on temporal proximity and statistical regularity

Authors

Gerhard Jocham, Kay H. Brodersen, Alexandra O. Constantinescu, ..., Mark E. Walton, Matthew F.S. Rushworth, Timothy E.J. Behrens

Correspondence

jocham@ovgu.de (G.J.), khbrodersen@gmail.com (K.H.B.)

In Brief

Jocham et al. demonstrate that learning is driven by several mechanisms operating in parallel, of which only one relies on knowing the relationship between outcomes and choices that cause them. This contingent learning is mediated by activity in orbitofrontal cortex.

Jocham et al., 2016, Neuron *90*, 177–190
April 6, 2016 ©2016 The Authors
http://dx.doi.org/10.1016/j.neuron.2016.02.018

Neuron
Article

Reward-Guided Learning with and without Causal Attribution

Gerhard Jocham,[1,2,3,7,*] Kay H. Brodersen,[1,5,7,*] Alexandra O. Constantinescu,[1] Martin C. Kahn,[1] Angela M. Ianni,[1,4] Mark E. Walton,[5] Matthew F.S. Rushworth,[5] and Timothy E.J. Behrens[1,6]
[1]Oxford Centre for Functional MRI of the Brain, Nuffield Department of Clinical Neurosciences, University of Oxford, John Radcliffe Hospital, Oxford OX3 9DU, UK
[2]Center for Behavioral Brain Sciences, Otto-von-Guericke-Universität Magdeburg, Universitätsplatz 2, 39106 Magdeburg, Germany
[3]Faculty of Economics and Management, Otto-von-Guericke-Universität Magdeburg, Universitätsplatz 2, 39106 Magdeburg, Germany
[4]Section on Integrative Neuroimaging, Clinical and Translational Neuroscience Branch, National Institute of Mental Health, National Institutes of Health, Intramural Research Program, Department of Health and Human Services, Bethesda, MD 20892, USA
[5]Department of Experimental Psychology, University of Oxford, South Parks Road, Oxford OX1 3UD, UK
[6]Wellcome Trust Centre for Neuroimaging, University College London, 12 Queen Square, London WC1N 3BG, UK
[7]Co-first author
*Correspondence: jocham@ovgu.de (G.J.), khbrodersen@gmail.com (K.H.B.)
http://dx.doi.org/10.1016/j.neuron.2016.02.018
This is an open access article under the CC BY license (http://creativecommons.org/licenses/by/4.0/).

SUMMARY

When an organism receives a reward, it is crucial to know which of many candidate actions caused this reward. However, recent work suggests that learning is possible even when this most fundamental assumption is not met. We used novel reward-guided learning paradigms in two fMRI studies to show that humans deploy separable learning mechanisms that operate in parallel. While behavior was dominated by precise contingent learning, it also revealed hallmarks of noncontingent learning strategies. These learning mechanisms were separable behaviorally and neurally. Lateral orbitofrontal cortex supported contingent learning and reflected contingencies between outcomes and their causal choices. Amygdala responses around reward times related to statistical patterns of learning. Time-based heuristic mechanisms were related to activity in sensorimotor corticostriatal circuitry. Our data point to the existence of several learning mechanisms in the human brain, of which only one relies on applying known rules about the causal structure of the task.

INTRODUCTION

An organism's ability to learn from behavioral outcomes is central to its evolutionary success. Recent decades have seen important advances in our understanding of the computations underlying many flavors of such reinforcement learning, but these models begin with a fundamental assumption, that organisms can attribute each outcome to the behavior that caused it, that is, they can assign the credit for an outcome correctly. Results of recent lesions studies have challenged this assumption, suggesting that learning is possible even when this simplest assumption is not met, and that these noncontingent mechanisms dominate behavior when lesions are made to the lateral orbitofrontal cortex (lOFC; Walton et al., 2010).

On initial consideration, several features of these results are surprising. When learning from rewards, the brain faces many complex computational problems. However, since typical neuroscience experiments separate behavior into discrete trials, there is no ambiguity about action-reward pairings and hence no apparent computational problem to solve. It appears paradoxical, then, that a brain region as evolutionarily recent as lOFC is required for this apparently trivial attribution. It is perhaps equally surprising, however, that any learning is possible in its absence. If an agent does not know which action led to which reward, how can it learn which actions are good at all?

Insights into these seeming conundrums can perhaps be gleaned by considering real-world ecological problems that exist outside the laboratory. In the real world, agents take many actions, and only some of them have consequences. These consequences may be delayed in time with many intervening irrelevant actions. Furthermore, many important outcomes are not even consequences of the agent's behavior. It becomes a difficult and important problem to discern which outcomes should cause learning, and on which actions (Sutton and Barto, 1998). In reinforcement learning terms, it becomes important to apply the correct state space during learning (Wilson et al., 2014).

One can think of different classes of mechanism for solving this problem. In precise contingent mechanisms, agents may be able to attribute particular outcomes to their causal actions due to external knowledge—if a cake is burned, it is more likely to be caused by the cooking time than the quantity of sugar in the recipe. Similarly, if experimental animals have extensive prior experience of outcomes following actions in a trial structure, they may learn to solve the attribution problem precisely even with a new set of experimental stimuli or type of reward.

In the absence of such external knowledge, it may still be possible to attribute outcomes to actions precisely by using

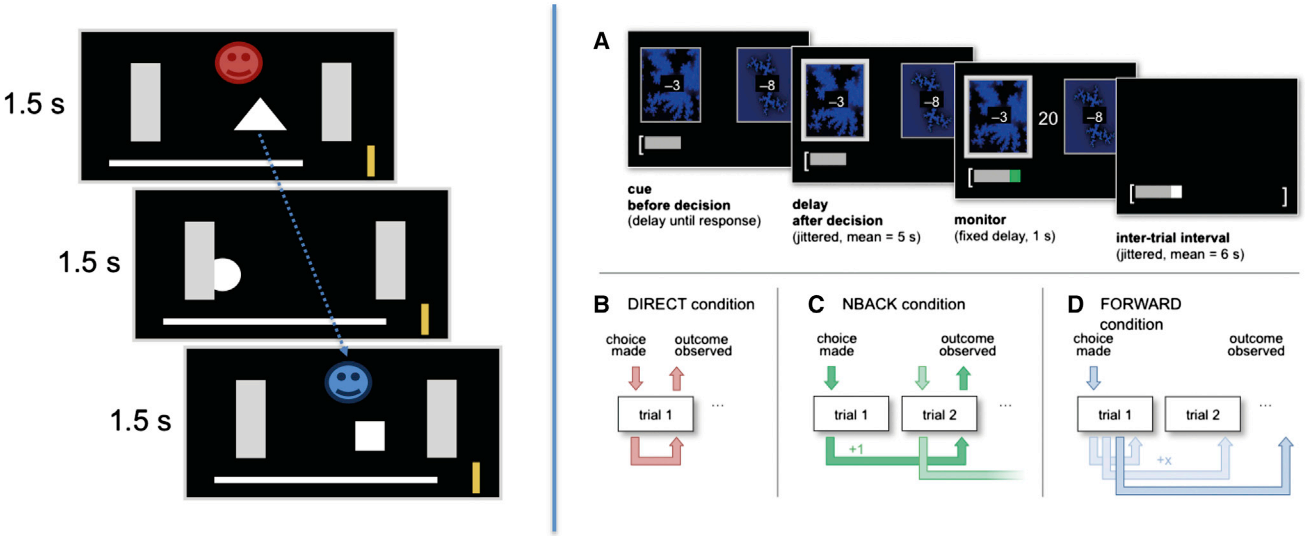

Figure 1. Task Schematic
Task schematic for experiment 1 (left) and experiment 2 (right).

heuristic mechanisms that capitalize on common features of causal relationships. For example, outcomes may be attributed to actions that immediately preceded them—a button press immediately followed by a loud explosion is unlikely to be repeated, but even a few seconds delay may prevent any such association being made. Here, agents can use a heuristic rule that is often true in real-world learning and has therefore been favored by evolution.

Even when attributions cannot be made precisely, they may still be made through statistical mechanisms. If one action has been taken more often than another, or has been pursued for a longer recent period of time, then it is more likely to be the cause of outcomes. Such considerations may lead to learning strategies familiar in ecological theories of behavior (Charnov, 1976) that state that if the time-average reward is high, then agents should continue with current behavioral policies.

Here, we show that in complex environments, healthy humans' behavior is guided by multiple learning mechanisms operating in parallel. While behavior was dominated by learning on the basis of precise contingent associations between outcomes and their causal choices, behavior also displayed hallmarks of simpler learning mechanisms that do not rely on such contingent associations. We found signals pertaining to the different learning mechanisms in separable brain circuits. Precise contingent learning was supported by a system centered on lOFC. Amygdala activity, or the absence of amygdala suppression, was related principally to statistical learning mechanisms. Proximal heuristic mechanisms were related to circuitry in motor regions of the cortico-striatal circuitry.

RESULTS

We performed two experiments to probe different mechanisms for credit assignment in the intact human brain using fMRI (Figure 1). Experiment 1 was designed to reveal signatures of each learning mechanism in normal behavior and harness fluctuations across the population to investigate their neural bases. Experiment 2 introduced manipulations that interfered with contingencies, allowing us to search for brain signals aligned with contingency, rather than reward or behavior. In both experiments, participants chose between different stimuli with independent probabilities to give rewards. These probabilities changed over time. Thus, subjects needed to continuously learn the stimulus-reward associations. To ensure that participants chose only the stimuli that were likely to give a reward, each choice incurred a cost.

In experiment 1, we aimed to simulate an ecologically realistic situation where many possible choices could be credited for a reward and only some rewards were caused by participant's behavior. We reasoned that credit assignment by precise contingent learning would be heavily taxed in such an environment, allowing the contribution of other mechanisms to become evident. A total of 23 participants (12 female) were presented with a continuous random succession of geometrical shapes (A / B / C). Shapes were moving across the screen from left to right, one at a time, during a period of 1.5 s (Figure 1, left). While on screen, these options could either be selected by pressing a specific button (incurring a small cost) or ignored. Critically, for a rewarded choice, participants received a contingent reward 3 s after the choice that caused it (subjects were informed and extensively pretrained on this delay). Thus, among the many choices participants made, they had to assign credit only to the specific choices made 3 s prior to reward delivery. However, in addition, subjects also received noncontingent rewards in a random fashion, independent of their behavior. Crucially, these two types of rewards were distinguishable (by color, red or blue, counterbalanced across subjects), and subjects were instructed to focus on contingent rewards and to ignore the noncontingent rewards. Thus, because subjects have to link contingent rewards to the choice made 3 s before rather than to the option currently observed, this design breaks the common "trial-like" structure for reward-guided learning

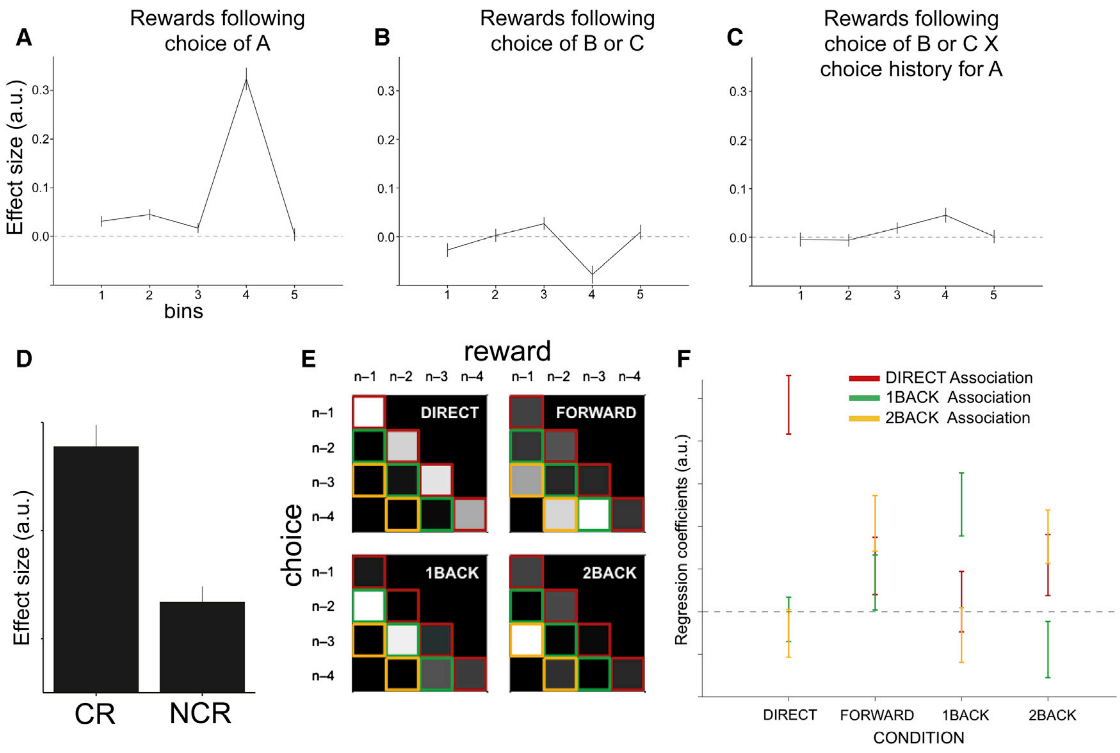

Figure 2. Behavioral Results

(A–C) Logistic regression results of experiment 1. Figures show how choices (0/1) of the option on the current trial are influenced by past rewards following choices of same option A (A); different options B or C (B); and again different options B or C, but depending on how often same option A had been chosen in the past 30 trials (C), depending on when the reward occurred relative to choice (bin 1, 0–0.5 s; 2, 0.5–1.5 s; 3, 1.5–2.5 s; 4, 2.5–3.5 s; 5, 3.5–4.5 s before reward). Values are mean ± SEM (across participants) of the regression coefficients obtained from the logistic regression.

(D) A separate linear regression shows that the average rate of responding in experiment 1 is, by definition, related to the rate of contingent rewards (CRs) but also to the rate of noncontingent rewards (NCRs), despite them being unrelated to behavior.

(E and F) Behavioral results of experiment 2. Using multiple logistic regression, we tested whether our instructions reliably induced contingent and noncontingent learning.

(E) Each box represents one condition, and each cell within a box represents a particular regressor. High parameter estimates are shown in white; low estimates in black. These regressors can be arranged into the lower quadrant of a square where the lead diagonal represents DIRECT learning (red), the next lower diagonal represents 1BACK learning (green), and the third diagonal 2BACK learning (yellow). For example, the first regressor in the top left box should receive loading if decisions under DIRECT instructions can be explained by a model in which any reward obtained on the previous trial (n – 1 column) is associated with the choice on that trial (n – 1 row). The plot shows that the DIRECT, 1BACK, and 2BACK conditions have predominantly yielded high parameter estimates in their respective red, green, and yellow regressors, while the FORWARD condition has led to loadings that are distributed across the different association types, as hypothesized.

(F) Averaging across the corresponding associations (red, green, and yellow diagonals, respectively) shows that the three different associations load differently depending on the instructed condition. See also Figures S1–S4.

All error bars represent SEM.

tasks. This design allowed us to quantify interindividual differences in learning from contingent and noncontingent rewards. The rate of noncontingent reward delivery was established during piloting to match that of contingent rewards across subjects.

Behavior Is Guided by Separable Contingent and Noncontingent Learning Mechanisms

To separate contingent from noncontingent learning in experiment 1, we used a multiple logistic regression to test how rewards following the choice of an option influenced the probability of choosing this same option the next time it was encountered, depending on when this reward occurred relative to the choice. Given that subjects were precisely instructed that rewards were given with a 3 s delay, in a subject relying exclusively on contingent learning, only those rewards occurring around 3 s after a choice should have an impact on reselecting that same stimulus. If, in contrast, subjects relied on noncontingent learning, then credit for rewards should spread back to noncausal choices made in the recent past.

We looked for these effects in five time bins before a reward (time bins were as follows: bin 1, 0–0.5 s; bin 2, 0.5–1.5 s; bin 3, 1.5–2.5 s; bin 4, 2.5–3.5 s; bin 5, 3.5–4.5 s). As expected, the effects of rewards depended on the time bin in which choices fell (ANOVA, effect of bin, $F_{4,116} = 94.01$, $p < 0.0001$). Consistent with a robust contingent learning mechanism, choices of stimulus A in time bin 4 ($t_{29} = 14.58$, $p < 0.0001$) markedly increased the probability of choosing A again in the future (Figure 2A). Subjects were therefore able to assign credit for a reward to its

causal choice despite the fact that there would often be another choice between the two events.

However, behavior was not exclusively driven by this precise contingent learning mechanism. Rewards following choices of A also increased the likelihood of future selections of A, but only if they occurred immediately after the choice (bin 1, t_{29} = 3.01, p = 0.0054; bin 2, t_{29} = 4.41, p = 0.0001), despite the fact that subjects were aware they were unrelated (Figure 2A). This involuntary spread of reward effect was specific to early time bins. While there was still a trend in bin 3 (t_{29} = 1.75, p = 0.091), rewards in later bin 5 had no effect on behavior (p > 0.77). Furthermore, the averaged effect in bins 1 and 2 was bigger compared to bin 3 (t_{29} = 1.76, p = 0.044, one-tailed) and to bin 5 (t_{29} > 2.43, p = 0.011, one-tailed). This effect of a reward not only reinforcing the choice that really led to its delivery but also other choices that occurred in close temporal proximity, was first described by Thorndike as early as 1933 (Thorndike, 1933) and has been termed "spread of effect." Here, we refer to this spread of effect to proximal choices as PROX.

To examine statistical credit assignment mechanisms, we first asked whether subjects might misassign credit for a reward to the wrong choice if that choice had commonly been taken in the past (Walton et al., 2010), as if the reward is being credited to the average behavioral policy, and not to the particular choice that caused it. While contingent rewards that followed B or C choices made the future selection of A shapes less likely on average (Figure 2B), this was not true at times when the subject had selected A often in the recent past. Indeed, contingent rewards following B or C choices increased future A choices as an increasing function of the frequency of A choices in the past 30 trials (ANOVA, effect of bin, $F_{4,116}$ = 2.45, p = 0.05; t test for bin 4, t_{29} = 3.14, p = 0.004; Figure 2C). That is, part of the credit for a reward following B or C choices was more likely to be misassigned to A the more often A had been selected in the recent past. We refer to this type of noncontingent learning as spread of effect to the recent history of choices (SoE_{Ch}). Importantly, this cannot be explained by a mere autocorrelation in subjects' choices. First, it predicts a switch away from the current choice of B onto the historical choice of A. Second, it is specific to rewarded choices. Third, it is specific to the contingent bin. Lastly, we included separate nuisance regressors in the regression model (see Experimental Procedures) to control for the main effect of choice history of A, the main effect of overall choice history, and the main effect of overall reward history. While the choice history of A had no effect (p = 0.3), the overall choice history had an effect, which, however, was negative and hence cannot explain the increased propensity to select option A (t_{22} = −4.1484, p = 0.0003). In addition, the overall rate of rewards increased subjects' propensity to select A (t_{22} = 5.09, p < 0.00002). Next, we followed this latter effect up by asking whether subjects may be more likely to select shapes if the recent average reward rate was high, even if this was driven by noncontingent rewards that were unrelated to the subjects' choices. We performed a separate regression that tested how the time-averaged rate of responding was dependent upon the time-averaged rate of contingent and noncontingent rewards (Supplemental Experimental Procedures, available online). By definition, the rate of responding depended on contingent rewards (as those are by design tied to responses). Importantly, however, response rates were also dependent on the rate of noncontingent rewards (t_{29} = 6.04, p < 0.00001; Figure 2D), indicating that the average rate of rewards increased the rate of responding. Thus, in addition to contingent learning, PROX, and SoE_{Ch}, participants' choices were also guided by a spread of effect to the recent history of rewards (SoE_{Rew}).

Notably, despite some relations (maximum r = 0.38), the dominant contingent learning and the three noncontingent learning mechanisms (PROX, SoE_{Ch}, and SoE_{Rew}) were largely uncorrelated across subjects (Figure S1 for full correlation matrix), suggesting separable mechanisms. The behavioral effects reported here are derived from 30 subjects, which include the 23 subjects that underwent scanning and an additional 7 subjects that took part in the final version of the behavioral pilot. Note, however, that we obtain an identical pattern of results when repeating the same analyses following inclusion of only the 23 fMRI subjects (Figure S2A).

While in experiment 1 we aimed to investigate how multiple credit assignment strategies vary naturally in the extent they guide learning, in experiment 2 we selectively manipulated learning strategies through task instructions. Participants made choices between two fractal stimuli according to three types of instructions that changed for each block of trials (Figures 1, right, and S2B). In each block the probability of each choice leading to reward was constant (Supplemental Experimental Procedures) In DIRECT blocks, outcomes were contingent on the choice in the same trial. To dissociate contingency from choices made in the same trial with the outcome, in NBACK blocks, outcomes were delayed by a known number of trials (one or two). Hence, they were contingent on a previous, but specific, choice. In FORWARD blocks, rewards were delayed by a small random number of trials that was not known to the subject, such that outcomes could no longer be linked contingently to any specific causal choice. This ensured that unlike in NBACK blocks, it was not clear on which specific choices outcomes were contingent. Note that while subjects cannot learn contingently in the FORWARD condition, learning is still possible using statistical mechanisms. That is, while they do not know which one of the preceding four choices (current, immediately previous, two, or three trials past) caused the reward observed, they can still assign the credit to the average choice. Despite these three different types of instructions, the true contingencies were always structured according to the FORWARD condition. Thus, across all conditions, rewards were delayed, or projected forward, by a random number of trials. This simple manipulation controlled for a number of critical factors across conditions (Supplemental Experimental Procedures). This setup allowed us to interrogate fMRI signals reflecting contingency, as they contain sequences of trials that are identical between conditions in all respects except for the instructed contingency between choice and outcome. Thus, the only difference between conditions was in the instructed contingencies.

Despite the true contingencies being identical in each condition, participant behavior was consistent with the three different instruction sets. Logistic regression (Supplemental Experimental

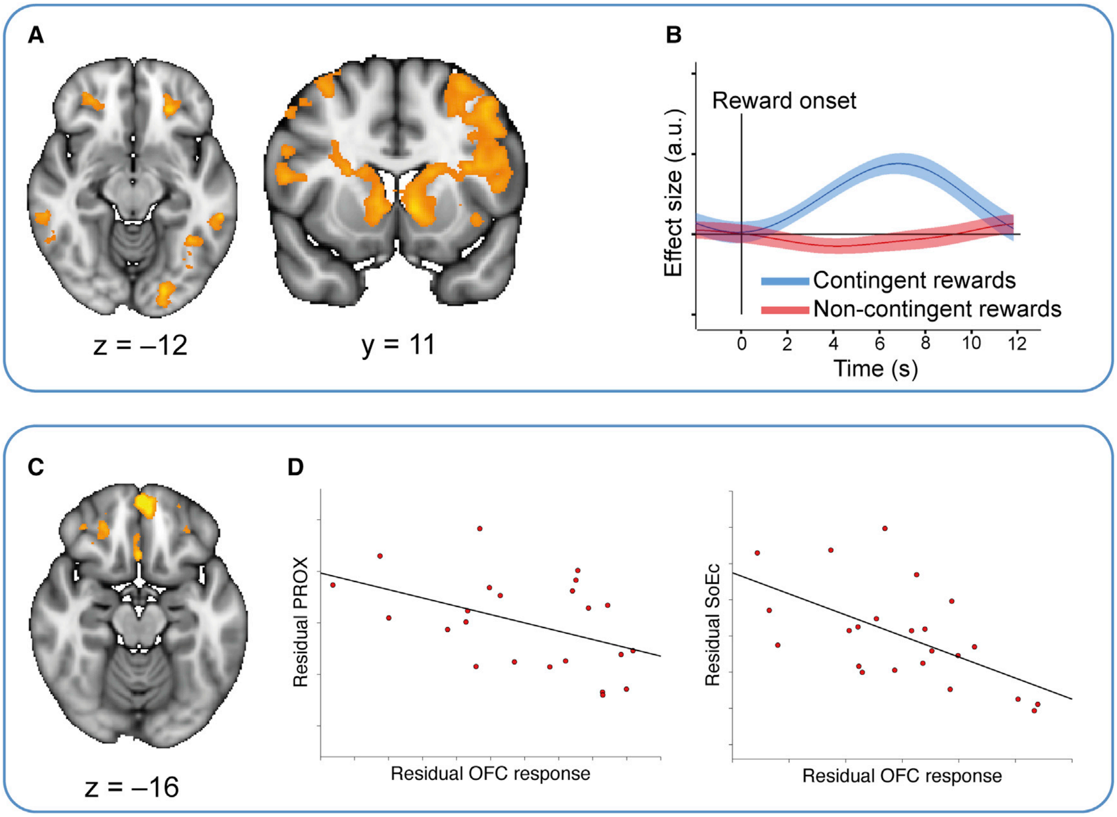

Figure 3. Contingent Reward Responses and Relation to Contingent Learning in Experiment 1

(A) Whole-brain results for the contrast contingent- noncontingent rewards in experiment 1.

(B) Inspection of the BOLD signal at the peak coordinate in lOFC shows that this region responds selectively to contingent, but not noncontingent, rewards. Solid lines show the mean and shaded areas the SEM of the regression coefficients across subjects. The black vertical line represents the time of outcome delivery. Values are mean ± SEM of regression coefficients across subjects

(C) Regression of the contrast in (A) against contingent learning versus PROX + SoE_{Ch} reveals that contingent reward responses in lOFC correlate with contingent learning behavior.

(D) Parameter estimates were extracted from the peak coordinate of the contrast in (A) and related to the different learning mechanism. The plots show that lOFC responses to contingent rewards are negatively related to noncontingent learning via PROX and SoE_{Ch}. The correlations are partial correlations, that is, after regressing out the effects of the respective other learning mechanisms from both parameters of interest. See also Figure S5 and Table S1.

Procedures) revealed a condition-by-trial interaction ($F_{6,138}$ = 8.62, $p < 0.0001$). Breaking these effects down showed that rewards increased future selections of the current choice in the DIRECT condition; the n – 1 and n – 2 choices in the 1BACK and 2BACK conditions, respectively; and all three previous choices in the FORWARD condition (Figures 2E and 2F). This demonstrates that subjects indeed deployed contingent learning in the DIRECT and NBACK conditions but noncontingent learning in the FORWARD condition, in which contingencies were unknown. Subjects were therefore able to exploit contingent learning mechanisms when contingencies were clearly discernible, but they were able to exploit noncontingent learning mechanisms when contingencies were unclear. Importantly, we ensured that the only difference between conditions was the instructed contingencies, while keeping all possible other factors comparable between conditions, such as subjects' rate of learning, the number of rewards earned, errors committed, and response times (Supplemental Experimental Procedures; Figures S3 and S4).

Signals Supporting Contingent Learning in lOFC

To search for brain regions linked to contingent learning, we examined the BOLD response at the time of the outcome in the conditions where subjects received instructions about the precise associations between stimuli and rewards: contingent and noncontingent rewards in experiment 1; reward and no reward in the DIRECT and NBACK conditions of experiment 2. We predicted that the lOFC would underlie precise contingent learning and, therefore, that subjects with more activity in this brain region would rely less on noncontingent learning.

We first harnessed the interindividual variability in learning strategies in experiment 1 and investigated their neural correlates. We contrasted BOLD responses between contingent and noncontingent rewards. While both serve as rewards, it is only during the former that a contingency between the reward and a choice has to be established. As the contingent rewards were the principal focus of subject attention, it is not surprising that this contrast revealed a large network of brain regions (Figure 3A; Table S1; whole-brain cluster corrected at $p < 0.001$;

cluster size threshold, $p < 0.05$), including the lOFC (MNI xyz = –24 mm, 35 mm, –12 mm, z max = 5.1 and xyz = 26 mm, 40 mm, –10 mm, z max = 4.59) and bilateral striatum, in particular in the ventromedial caudate nucleus (MNI xyz = –8 mm, 11 mm, –1 mm, z max = 5.16; and xyz = 10 mm, 14 mm, 1 mm, z max = 4.69; Figure 3A). Visualization of this difference effect in the lOFC revealed that it was driven exclusively by the positive (contingent rewards) portion of the contrast, and not the negative (noncontingent rewards) portion (Figure 3B). From each subject's behavior, we computed the ratio of contingent learning to noncontingent mechanisms. We asked whether the aforementioned BOLD contrast [contingent rewards – noncontingent rewards] in any voxels would predict the extent to which subjects relied on precise contingent relative to noncontingent learning strategies (contingent learning versus PROX + SoE_{Ch}). Here we considered the two noncontingent mechanisms that were contributed to by the contingent rewards (PROX and SoE_{Ch}), reflecting the fact that the BOLD contrast at the first level was derived from these rewards (PROX can arise by misattribution of either contingent or noncontingent rewards to proximal choices; SoE_{Ch} specifically arises by misattribution of contingent rewards to the average choice history. In contrast, SoE_{Rew} is specifically defined as the effect of noncontingent rewards). The only brain region to show a significant effect across subjects was in the OFC, including the lOFC ($p < 0.01$, cluster-based correction at $p < 0.05$; Figure 3C). Furthermore, extracting parameter estimates from the peak lOFC coordinate (from the main contrast contingent minus noncontingent rewards) revealed that lOFC activity was inversely related to both noncontingent learning mechanisms ($r = -0.46$, $p = 0.03$ and $r = -0.58$, $p = 0.0034$, for PROX and SoE_{Ch}, respectively; Figure 3D), with no significant difference ($t_{22} = -0.15$, $p > 0.55$). Subjects with strong lOFC responses to contingent rewards were therefore less likely to exhibit either form of noncontingent learning relative to accurate contingent learning.

Experiment 2 allowed us to further isolate lOFC's role in contingent learning, as it contained sequences of trials identical in all respects except for contingency. We considered sets of three trials pertinent to any particular outcome (+ and – denote reward and nonreward outcomes, respectively): the past trial ($n-1$ or $n-2$), the current trial (n) and the following trial ($n+1$). For example, in the sequence "BA+B," the subject switched from a B choice in the previous trial to an A choice in the current trial, received a reward, and then switched back to B the following trial. In order to examine contingency, we examined BOLD activity at the time of this outcome and contrasted trials in which the "following" choice respected the contingencies of the outcome against those where it did not. For example, BA+A and BA-B are contingent sequences in DIRECT blocks because the subject acted in accordance with the outcome (stay with rewarded A or switch back from unrewarded A). By contrast, in NBACK blocks, these same sequences are noncontingent because the outcome pertained to the preceding B, rather than the proximal A. Similarly, [BA-A, BA+B] are noncontingent sequences in DIRECT blocks but contingent sequences in NBACK blocks. To control for block differences, [AA+A, AA-B] are contingent and [AA-A, AA+B] noncontingent in all conditions. It is notable that comparisons between contingent and noncontingent sequences are controlled both within and across conditions for choices, outcomes, and switches but, on average, distinguish outcomes that caused contingent learning from those that did not.

We extracted data from an ROI in the lOFC selected from an orthogonal contrast (see Supplemental Experimental Procedures for ROI selection). In line with a contingency-related response, BA+A caused greater lOFC activity than BA-A in DIRECT, but not NBACK, blocks (Figure 4A; difference, $t_{23} = 3.28$, $p = 0.002$), and BA+B caused greater lOFC activity than BA-B in NBACK, but not DIRECT, blocks (Figure 4B; difference, $t_{23} = 3.03$, $p = 0.003$). Combining these two effects according to DIRECT contingencies revealed a positive effect in DIRECT blocks ($t_{23} = 2.45$, $p = 0.01$) and a negative effect in NBACK blocks ($t_{23} = -2.72$, $p = 0.006$), where contingencies were reversed (Figure 4C). Notably, repeating this analysis for sequences that began AA, where contingencies were identical across blocks (Figure 4D), revealed a positive response in both conditions (DIRECT, $t_{23} = 2.56$, $p = 0.009$; NBACK, $t_{23} = 2.74$, $p = 0.006$; difference not shown). Hence, across all tests, lOFC responses were aligned with contingencies rather than rewards or behavior (Figure 4E; $t_{23} = 3.77$, $p = 0.0005$; Figure 4F; $t_{23} = 3.65$, $p = 0.0007$). This is particularly notable in light of previous theories of lOFC function that have argued for error processing (Kringelbach and Rolls, 2004; Fellows, 2007) or behavioral switching (Jones and Mishkin, 1972; Dias et al., 1997; Chudasama and Robbins, 2003) to be cardinal functions of the region.

Further to the effects in the lOFC, it is noteworthy that at the whole-brain level, contrasting contingent with noncontingent trials revealed a network of brain regions very similar to that found in experiment 1 when contrasting contingent with noncontingent rewards (Figure S6). In particular, these included lOFC (MNI xyz = –24 mm, 40 mm, –16 mm, z max = 3.8), bilateral ventral striatum (MNI xyz = ±16 mm, 10 mm, –14 mm, z max = 4.1), and lateral prefrontal cortex (MNI xyz = 42 mm, 32 mm, 22 mm, z max = 4.1).

Amygdala Responses Mediate Noncontingent Learning

Across experiments, neural signals were therefore consistent with contingent learning mechanisms in a network of frontostriatal brain regions, with the strongest behavioral impact in the lOFC. However, in experiment 1, subjects also deployed three noncontingent learning strategies, PROX, SoE_{Ch}, and SoE_{Rew}. On the basis of previous lesion data from both macaques and rodents (Stalnaker et al., 2007; Rudebeck and Murray, 2008), we hypothesized that the amygdala might play a key role for at least some of these noncontingent mechanisms.

Amygdala lesions facilitate reversal learning in monkeys (Rudebeck and Murray, 2008) and restore the ability to perform reversals after OFC lesions in rodents (Stalnaker et al., 2007). Since OFC reversal deficits are reflective of deficits in precise contingent learning (Walton et al., 2010), it is conceivable that amygdala activity at the time of a reward might downweight precise associations in favor of statistical ones. In our experiment 1, such an argument makes two predictions. First, it predicts that amygdala activity at the time of contingent rewards would lead to less contingent and greater statistical learning (which is maladaptive in the current task). Second, it predicts that amygdala

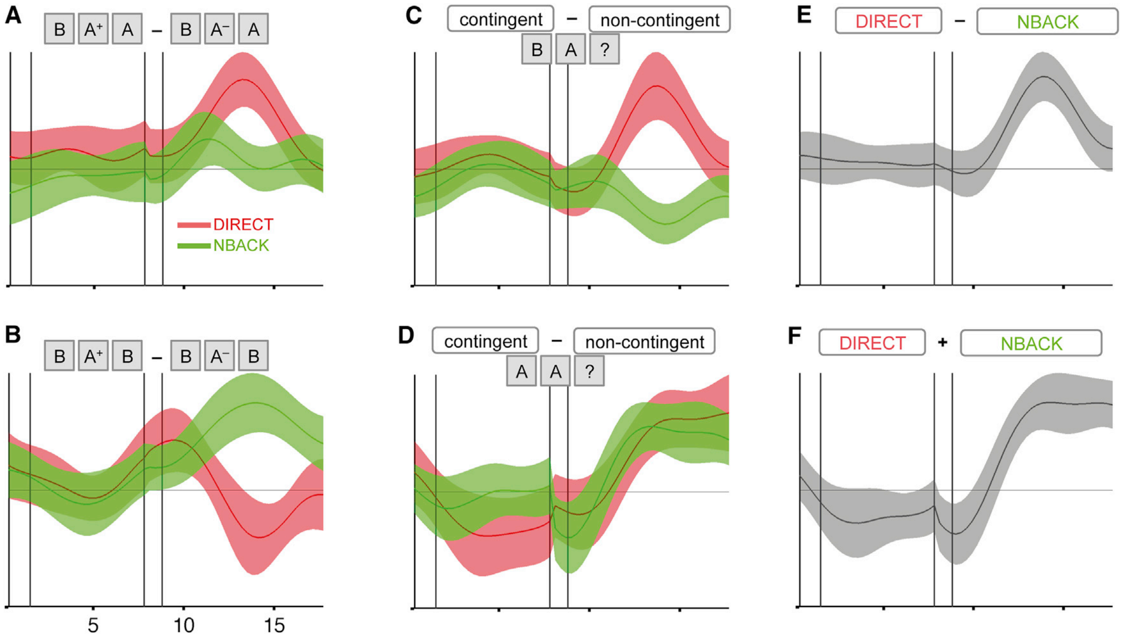

Figure 4. Analysis Testing Whether the OFC Signal in Experiment 2 Fulfils the Criteria of a Signal Encoding Associations between Outcomes and Their Causal Choices

Each panel shows the observed temporal evolution of a GLM contrast over intratrial time (contrast parameter estimates ± SEM). Data are averaged across all OFC voxels that survived the (orthogonal) contingency contrast on AA? triplets. Outcomes (reward/nonreward) refer to the outcome of the middle trial in each triplet. Vertical bars separate decision, delay, outcome, and interval phases.

(A) Consistent with a signal encoding contingent associations between choices and outcomes, only in the outcome phase of DIRECT trials do contingent associations elicit an increased lOFC signal.

(B) In NBACK blocks, it is the noncontingent trials that yield lOFC activity.

(C) Taken together, contingent and noncontingent trials lead to exactly opposite signals in DIRECT and NBACK blocks (addition of the contrasts [BA+B - BA−B] + [BA−B - BA+B]).

(D) In AA? triplets, contingent choices are identical in DIRECT and NBACK blocks; accordingly, lOFC shows the same effect in both conditions (contrasting [BA+B - BA−B] - [BA−B - BA+B] triplets).

(E) BA? triplets show a highly significant contingency effect in the lOFC.

(F) Thus, AA? triplets show an equally strong contingency effect as BA? triplets. Overall, the figure shows that lOFC activity is incompatible with predictions made by the reward and reward prediction error hypotheses but corresponds precisely to the predictions made by the contingency hypothesis. Note that all plots were produced by right-aligning data from the decision phases so as to line up with the decisions themselves. The jittered duration of the delay phase thus causes a discontinuity between the delay and the monitor phases in this visualization. See also Figure S6.

activity at the time of free rewards would mean these free rewards were less likely to be treated as contingent rewards (which is adaptive in the current task).

To address this second prediction, we searched for brain regions whose responses to noncontingent rewards were related to noncontingent learning. Consistent with the mechanism described above, we found clusters bilaterally in the amygdala and anterior hippocampus that exhibited a negative correlation with noncontingent learning (MNI xyz = –19 mm, –3 mm, –21 mm, z max = 4.06 and xyz = 24 mm, –9 mm, –18 mm, z max = 4.25; whole-brain cluster corrected at $p < 0.01$; cluster size threshold, $p < 0.05$; Figure 5A). Subjects with large responses to free rewards in these regions were thus unlikely to inaccurately treat these free rewards as contingent. Extracting parameter estimates from the peak coordinate revealed that subjects with strong amygdala responses to noncontingent rewards relied less on all three noncontingent learning mechanisms ($r = -0.67$, -0.71, and -0.81 for PROX, SoE_{Ch}, and SoE_{Rew}, respectively; all $p < 0.0006$; Figure 5B). Notably, while the amygdala response correlated equally strongly with PROX and SoE_{Ch} ($t_{22} = 0.81$, $p > 0.4$), it correlated more strongly with SoE_{Rew} compared to both SoE_{Ch} and PROX ($t_{22} = 4.72$ and $t_{22} = 4.41$, $p < 0.0003$). Moreover, in contrast to the lOFC, contingent rewards had no significant effect in the amygdala and did not predict any marker of learning (all $t < 0.73$, $p > 0.47$).

We extracted data from the peak amygdala coordinate from this cluster to test our first prediction. Amygdala activity should be suppressed to allow contingent learning from contingent rewards. We first note that when taken on average over the group, amygdala activity is indeed suppressed after subjects make a response in anticipation of a contingent reward (Figure 5C; $t_{22} = -3.75$, $p = 0.001$). Furthermore, across subjects, this suppression is negatively related to the two statistical learning mechanisms ($t_{22} = 2.7$ and $t_{22} = 3.06$, $p = 0.013$ and $p = 0.006$; SoE_{Ch} and SoE_{Rew}, respectively). Subjects who do not exhibit this suppression will learn statistically, not contingently, from

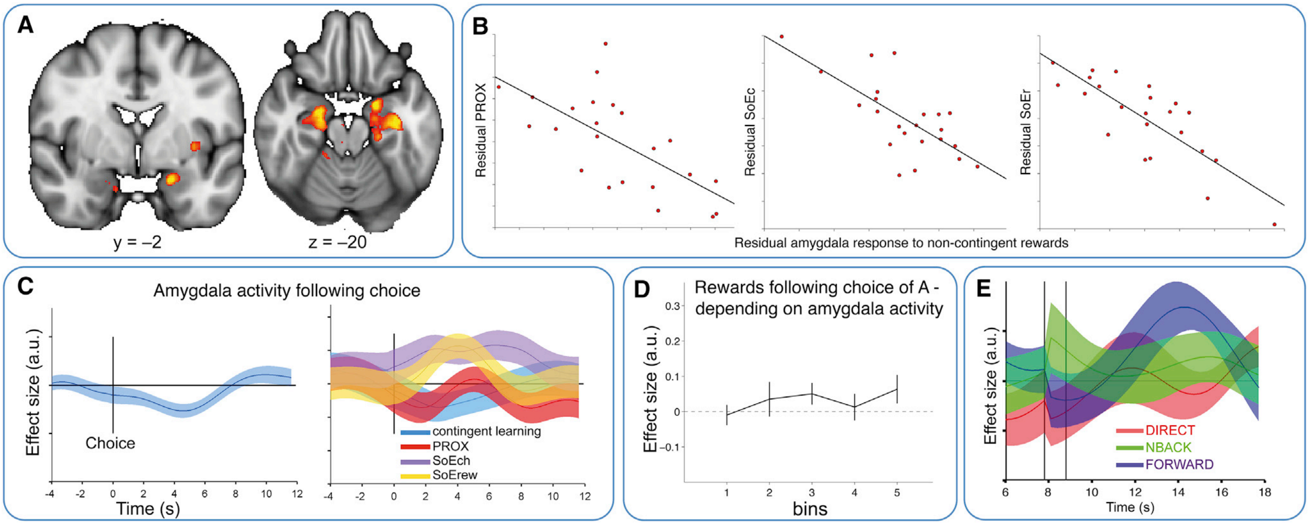

Figure 5. Amygdala and Noncontingent Learning

(A) In experiment 1, stronger amygdala responses to noncontingent rewards correlate with better contingent relative to noncontingent learning.

(B) Extraction of parameter estimates from the peak coordinate of the above contrast in (A) shows that amygdala responses to noncontingent rewards in experiment 1 correlate negatively with all three noncontingent learning mechanisms, albeit the correlation with SoE_{Rew} was more pronounced than that with either SoE_{Ch} or PROX (see main text). The correlations are partial correlations, that is, after regressing out the effects of the respective other learning mechanisms from both parameters of interest.

(C) Following a choice, the amygdala signal was suppressed (left). Amygdala activity after a choice (in anticipation of a contingent reward) correlated positively with SoE_{Ch} and SoE_{Rew} (right), meaning that a lack of amygdala suppression was associated with misassignment of the following reward via one of these noncontingent mechanisms.

(D) On a trial-by-trial level, credit for a reward following choice of A was likely to be misassigned to one of the noncontingent bins when amygdala activity was high in the period between choice and reward.

(E) In experiment 2, amygdala was exclusively reward sensitive in the FORWARD condition, the only condition where learning was only possible from spreading credit for a reward to the average choice history. The graph shows the evolution of a simple "reward-no reward" contrast over intratrial time as in Figure 4, taken from the peak coordinate from experiment 1 shown in (A).

Solid lines in (C) and (E) show the mean; shaded areas and error bars in (D) represent the SEM of the contrast estimates across subjects. The black vertical lines in (E) represent the time of choice and outcome delivery, respectively

the contingent rewards. Despite the absence of an effect on PROX, the effect survives the averaging over all three noncontingent mechanisms ($t_{22} = 3.68$, $p = 0.0013$).

In order to strengthen this argument within subjects, we designed a novel analysis strategy that examined the relationship between this amygdala suppression and noncontingent learning on a choice-by-choice basis within a single subject. We fit separate hemodynamic response functions to the amygdala activity after every button press. This resulted in a vector of parameters describing the amygdala response to each button press. We then performed a new behavioral regression like the regression in Figures 2A–2C, but now each behavioral regressor was paired with a second regressor: the interaction of itself and the (demeaned) amygdala response. This regression therefore asks whether the amygdala responses predict how the reward will impact future behavior. Despite the noisy nature of single-trial fMRI fits, a pattern emerged in which increased amygdala activity before rewards (the absence of amygdala suppression) led to noncontingent learning. If amygdala activity was high following choice of A, then rewards in the noncontingent bins made future choices of A more likely (Figure 5D; average over all bins, $t_{22} = 2.58$, $p = 0.017$).

Together, these results suggest that amygdala responses in the anticipation and delivery of reward lead to a reduction of precise contingent learning from that reward. More activity to free rewards makes it less likely that those rewards will be falsely treated as contingent. Activity is suppressed in anticipation of contingent rewards. The absence of this suppression makes it more likely that contingent rewards will be treated statistically (rather than contingently) and more likely that intervening free rewards will be mistaken for contingent ones. We investigated this effect further by examining amygdala reward responses in experiment 2, which included an explicit experimental manipulation to control contingent learning. We extracted signal from the above peak coordinate identified in experiment 1 (MNI xyz = –19 mm, –3 mm, –21 mm) and compared responses in the DIRECT and NBACK conditions, where rewards could be attributed to particular choices in the past, to those in the FORWARD condition, where rewards could not be assigned to any particular choice but nevertheless reinforced current broad behavioral policies (statistical learning). While activity in the amygdala did not distinguish rewards from unrewarding outcomes in either of the two contingent conditions ($t_{23} = 1.68$ and 0.95, $p > 0.1$ and $p > 0.34$; DIRECT and NBACK, respectively), it exhibited a clear reward effect in the noncontingent FORWARD condition (Figure 5E; $t_{23} = 3.46$, $p < 0.003$; difference between FORWARD and DIRECT, $t_{23} = 2.35$, $p = 0.014$, one-tailed; difference between FORWARD and NBACK, $t_{23} = 1.84$, $p = 0.04$, one-tailed).

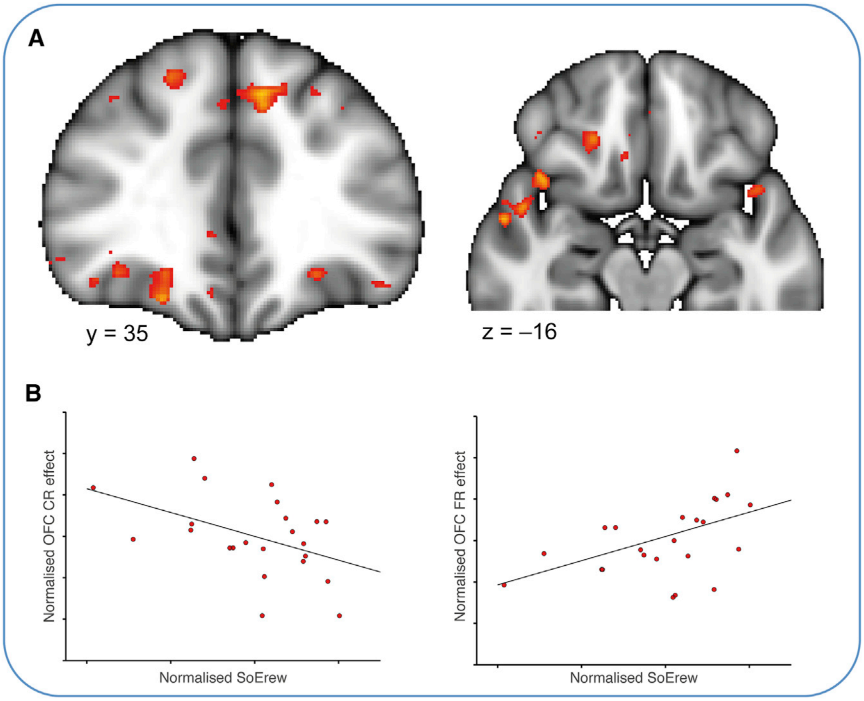

Figure 6. Connectivity of lOFC with VMS

(A) VMS connectivity with lOFC during contingent versus free rewards is related to better contingent relative to noncontingent learning.

(B) Increased VMS-lOFC connectivity during contingent rewards is related to decreased SoErew, whereas the opposite pattern is found for connectivity during free rewards. Correlations are partial correlations, that is, after regressing out the effects of the respective other learning mechanisms from both parameters of interest.

lOFC Interactions with Ventral Striatum

In our main contrast of contingent versus free rewards, we found, in addition to the effect in lOFC, a prominent effect in ventromedial striatum (VMS). While this effect, unlike the lOFC effect, did not correlate with behavior across subjects, it is plausible that interactions between lOFC and VMS underlie precise contingent learning. VMS receives dense projections from lOFC (Selemon and Goldman-Rakic, 1985), and, together, the two structures are part of a key circuit underlying goal-directed learning (Yin and Knowlton, 2006). We therefore performed a psychophysiological interaction analysis (PPI, see Supplemental Experimental Proceduresfor details) to test whether increased coupling between lOFC and VMS during contingent versus free rewards supports contingent learning. We extracted data from the peak coordinate in the VMS (MNI xyz = –8 mm, 11 mm, –1 mm and xyz = 10 mm, 14 mm, 1 mm) and searched for regions in which coupling with this seed region was related to individual differences in learning styles. In line with our hypothesis, we found regions in bilateral lOFC in which higher coupling with VMS during contingent versus free rewards was related to better contingent relative to noncontingent learning (uncorrected at $p < 0.001$, MNI xyz = –24 mm, 36 mm, –11 mm, z max = 3.17 and xyz = 22 mm, 36 mm, –19 mm, z max = 3.58; Figure 6A). We extracted parameter estimates from this coordinate to test whether this effect could be specifically related to connectivity during receipt of contingent or free rewards. We found that connectivity during contingent rewards was associated with diminished SoE_{Rew} ($r = -0.47$, $p = 0.029$), whereas free reward connectivity was related to increased SoE_{Rew} ($r = 0.5$, $p = 0.016$; Figure 6B). The other learning parameters, while generally showing a similar pattern, did not reach significance.

Midbrain and Dorsolateral Striatal Reward Responses Promote Noncontingent Learning

In experiment 1, we found that credit for a contingent reward was not only assigned correctly to the causal choice but also incorrectly to temporally proximal choices (PROX; Figure 2A) and to the average history of recent choices (SoE_{Ch}; Figure 2C). Using the same contrast [contingent rewards – noncontingent rewards] as above for lOFC, we found a region of the midbrain showing the opposite relation to behavior as lOFC. In this midbrain region, consistent with the location of dopaminergic cell groups of the ventral tegmental area (VTA) and pars compacta of the substantia nigra (SN_C), responses to contingent rewards correlated negatively with the degree to which subjects deployed precise contingent learning as opposed to both PROX and SoE_{Ch} (MNI xyz = –5 mm, –16 mm, –19 mm, z max = –3.66 and xyz = 6 mm, –15 mm, –21 mm, z max = 3.48; $p < 0.001$, uncorrected; Figure 7A). Please note that this contrast did not survive cluster-based thresholding, which, however, is unsurprising given the small size expected of midbrain clusters. We extracted parameter estimates from the peak location of this correlation to test if this effect could be specifically related to PROX, or if it was more generally related to overall noncontingent learning. At this peak location, responses were strongly related to PROX ($r = 0.66$, $p < 0.001$; Figure 7A) and to SoE_{Ch} ($r = 0.52$, $p = 0.01$), but not to SoE_{Rew} ($r = 0.23$, $p = 0.28$). Furthermore, direct comparison revealed that midbrain activity was, by trend, more strongly related to PROX than to SoE_{Ch} ($t_{22} = 1.65$, $p = 0.0566$). These results suggest that VTA/SNC responses to contingent rewards may lead to part of the credit for these rewards being misassigned to both proximal choices and to the average choice history.

We reasoned that PROX might arise because the close temporal coincidence of the reward-evoked dopamine release with a motor command in regions such as dorsolateral striatum would “stamp in” such stimulus-response associations (Redgrave and Gurney, 2006; Yin and Knowlton, 2006). Following this logic, the magnitude of reward responses in sensorimotor striatal regions will depend on the delay between choice of a stimulus and the outcome, with sooner rewards being more effective. We investigated this by setting up a parametric contrast in which all rewards were modulated by the time elapsed since the last action.

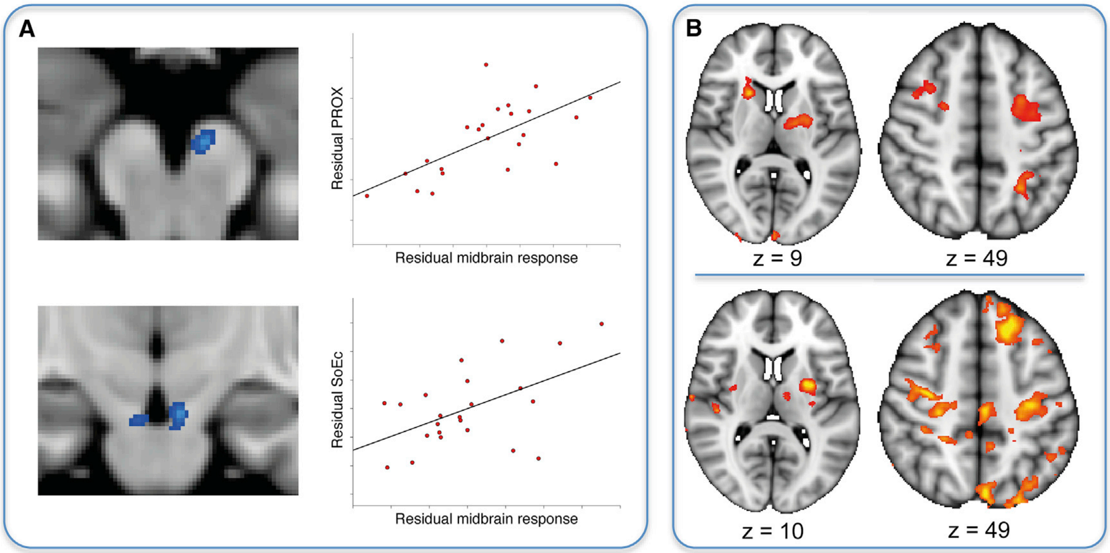

Figure 7. Relationship of Midbrain and Dorsal Striatal Reward Responses to Noncontingent Learning in Experiment 1

(A) Midbrain responses to contingent rewards in a region consistent with the location of substantia nigra and ventral tegmental area correlate negatively with the degree to which subjects' behavior was guided by contingent learning as opposed to either PROX or SoE_{Ch}—the exact opposite pattern of what was observed in lOFC (see Figures 3C and 3D). Contingent reward responses at the peak location were strongly related to both PROX and SoE_{Ch}; however, the correlation with PROX tended to be stronger than that with SoE_{Ch}.

(B) A parametric contrast revealed that rewards elicited a stronger response the sooner they occurred following a choice (upper row). In areas associated with model-free learning such as putamen and associated motor cortical areas, this effect was strongly related to the extent subjects' behavior was guided by PROX (bottom row).

We found that rewards delivered soon after a choice evoked responses in the putamen, the rostral caudate, and the bilateral premotor cortex ($p < 0.01$, cluster corrected at $p < 0.05$; Figure 7B, upper row). This network, unlike the circuitry involving lOFC and ventral striatum responsive to contingent rewards, has been implicated in learning of stimulus-response habits in a habitual fashion (Yin and Knowlton, 2006). Importantly, we found that this effect in bilateral putamen (MNI xyz = –30, –3, 10, z max = 4.09 and xyz = 29, –7, 5, z max = 3.43) and bilateral motor cortex (MNI xyz = –29, –21, 45, z max = 3.9 and xyz = 35, –14, 53, z max = 4.43) was stronger in subjects who relied more on PROX ($p < 0.01$, cluster corrected at $p < 0.05$; Figure 7B, bottom row). To test whether these timing-dependent reward effects were specifically related to PROX or to both PROX and SoE_{Ch}, we extracted parameter estimates from independent peak coordinates in the putamen and motor cortex to test for differential correlation. The peak was selected from a contrast of the correlation with both PROX and SoE_{Ch}, thus avoiding bias toward either of the two mechanisms. In both putamen and motor cortex, the response was strongly related to both PROX (t_{22} = 5.04 and t_{22} = 5.32, $p < 0.00005$) and SoE_{Ch} (t_{22} = 2.82 and t_{22} = 2.65, $p < 0.015$), but not SoE_{Rew} ($p > 0.2$). Direct contrasts further revealed a significantly stronger correlation with PROX compared to SoE_{Ch} in both areas (t_{22} = 2.59 and t_{22} = 2.9, $p < 0.02$). Thus, reward modulated activity in both putamen and motor cortex depending on the timing relative to a choice, and this modulation was related to noncontingent learning in both structures. While proximity-based reward responses correlated with both PROX and SoE_{Ch}, they did not correlate with SoE_{Rew}, and the correlation with PROX was more pronounced than that with SoE_{Ch}. This provides further evidence that PROX and SoE_{Ch} are not only dissociable behaviorally but also neurally. It is also important to note that this pattern is different to that found in the lOFC, where responses to contingent rewards were negatively related to both PROX and SoE_{Ch} to the same extent. Thus, while contingent reward responses in lOFC appear to generally suppress noncontingent learning from contingent rewards, proximity-dependent reward responses in putamen and motor cortex appear to be predominantly associated with PROX, i.e., with spreading credit for a reward to very recent choices.

DISCUSSION

We have shown that in a dynamic environment, the choices of healthy participants are guided by both precise contingent and noncontingent learning mechanisms that are separable both behaviorally and at the neural level. Behavior was dominated by learning that reflected the true choice-outcome contingencies. Such learning appeared to rely in part on lOFC. However, we were also able to identify other learning mechanisms that assigned outcomes to incorrect choices. Two of them were statistical learning mechanisms that learned through time-averaged choices and rewards. These behaviors appeared to rely in part on amygdala responses both in anticipation and receipt of rewards. Lastly, we identified a "heuristic" learning mechanism whereby rewards were inaccurately paired with choices that immediately preceded them. This direct

action-outcome pairing was predicted by responses in the motor corticostriatal circuitry.

In healthy macaques, precise contingent learning is usually so powerful that it dwarves the influence of other learning mechanisms. The contribution of these noncontingent mechanisms only becomes evident after lesions to lOFC (Walton et al., 2010). Likewise, for healthy human volunteers, credit assignment is trivial on standard reinforcement learning tasks, where there is usually only one choice and one outcome per trial. By breaking with the typical trial-based structure and by randomly delivering noncontingent rewards, we were able to create a scenario that is more akin to a naturalistic environment, where several responses could be candidate actions for a given outcome. This allowed noncontingent learning mechanisms to become more pronounced and, thus, to be isolated along the dominant contingent learning mechanism. It is likely that in real-life situations with many candidate actions and intervening outcomes, the effect of these noncontingent mechanisms is even more pronounced. We identified three such noncontingent mechanisms. First, rewards that occurred very close in time to a particular action tended to reinforce that action whether or not it caused the reward (PROX). This heuristic mechanism is reminiscent of the steeply diminishing effect of reinforcement with increasing delay between conditioned stimulus or instrumental action and reinforcement (Kamin, 1961; Dickinson et al., 1992). It also bears a resemblance to the emergence of superstitious behaviors during operant conditioning, where behaviors unrelated to reward are often reinforced due to their temporal proximity to reward delivery (Skinner, 1948; Devenport, 1979). Second, subjects were likely to assign credit for a reward to a choice that was frequently selected in the recent past, whether or not it was causally related to the reward (SoE_{Ch}). Third, subjects were likely to make choices more frequently during periods when they were rewarded frequently, even if those choices did not cause the reward (SoE_{Rew}). While such statistical mechanisms can be catastrophic when contingencies change abruptly from one trial to the next, they do not impede learning in situations where contingencies are stable or smoothly varying (Walton et al., 2010). Indeed, related strategies based on average recent reward rates may be beneficial in foraging-style decisions, which learn only the relative value of pursuing or switching from a current ongoing strategy (Charnov, 1976). There are also many real-world examples where learning via PROX or SoE_{Ch} may be adaptive. Contingent learning may be led astray when assumptions about the causal structure of the task are inaccurate, as is the case in the confirmation bias (Doll et al., 2011). In situations such as motor learning, PROX is adaptive because causality is closely tied to temporal proximity. Likewise, statistical learning mechanisms that average long-term rewards and choices are adaptive in situations where it is unclear which precise outcomes relate to which precise choices.

The noncontingent learning mechanisms we investigate in this study do not reflect the loss of all credit assignment between stimulus and reward. Rather, credit assignment in these mechanisms happens either statistically (because stimuli have often been chosen during rewarding periods) or heuristically (because a reward happened to occur immediately after a stimulus was chosen). Indeed, what is unique about the precise contingent mechanism is that the credit for an individual reward is attributed to a precise individual selection of the relevant stimulus in an appropriate fashion, reflecting the (accurate) knowledge that the choice caused the reward to occur. This knowledge may be gained through instructions, as in the current report, or through extensive experience on the learning problem, as in the original report of lesions to macaque OFC (Walton et al., 2010). The factors that determine the relative contribution of precise contingent learning and noncontingent mechanisms are, to our knowledge, not known. It is possible that uncertainty about the causal structure of the world is one factor that promotes statistical learning.

The contingent and noncontingent learning mechanisms we identified were anatomically separable. While a large network of brain regions was more active during receipt of contingent as opposed to noncontingent rewards in experiment 1 (likely reflecting an attentional effect), lOFC was the only one of these areas to show a clear relationship to contingent learning. Subjects with the strongest responses to contingent rewards in this region were least likely to misassign these rewards via either PROX or SoE_{Ch}. Similarly, connectivity between lOFC and VMS during receipt of contingent rewards was related to better contingent learning. Furthermore, experiment 2 allowed us to dissect the OFC reward signal in precise detail. By comparing triplets of trials that were identical in all respects except for the instructed contingencies, we could show that the same reward in a given triplet had opposite effects depending on whether the reward had to be associated with the choice on the current trial or with the alternative choice on the previous trial. Thus, our results are consistent with lOFC encoding the exact kind of signal required to solve the credit assignment problem, that is, to associate a reward with the choice that caused it (Sutton and Barto, 1998). These data are in agreement with studies showing that OFC neurons flexibly encode the reward-predictive properties of stimuli (Thorpe et al., 1983; Schoenbaum et al., 1998; Tremblay and Schultz, 1999; Padoa-Schioppa and Assad, 2008; Morrison and Salzman, 2009). Accordingly, lesions to the OFC reliably produce deficits in adjusting behavior to changes in stimulus-outcome associations (Mishkin, 1964; Jones and Mishkin, 1972; Dias et al., 1996; Izquierdo et al., 2004). These deficits resulted from credit being distributed inappropriately to choices that were made proximal in time to the outcome and to the average choice history (Walton et al., 2010). This strongly suggests that OFC is essential for contingent learning. Our data support this view: (1) BOLD signals in lOFC displayed the hallmarks of a signal encoding contingent associations between outcomes and the choices that caused them, and (2) lOFC responses to contingent rewards were related to learning strategies.

The ability to learn causally in reinforcement learning is reliant on correct knowledge of the state space, or causal structure, of the learning problem. Indeed, the four learning mechanisms we have described here might be interpreted mathematically as different instantiations of the task state space—only one of them correct—and there are clearly other possible instantiations. In our case, this state space defines which stimuli might lead to which outcomes. Closely related theories of OFC function posit that OFC activity is responsible for inferring and maintaining

knowledge of this state space (Takahashi et al., 2011; Wilson et al., 2014). Critically, knowledge of the state space is orthogonal to another common distinction in learning theory, the division between model-based and model-free learning (Daw et al., 2005, 2011; Dayan and Daw, 2008; Dayan and Niv, 2008). Both model-based and model-free learning require a correct knowledge of the state space and correct contingent updating (Wilson et al., 2014). In our experiments, subjects were explicitly informed about the causal structure of the task (even though this information was misleading in experiment 2). Thus, our results speak to the lOFC's role in leveraging this knowledge of the state space, but not to the issue of how or where in the brain this structure might be learned or inferred from experience. Furthermore, while our task was an instrumental learning task, the role of lOFC in this task likely is in representing stimulus-outcome associations (Schoenbaum et al., 2009), rather than action-outcome associations, which instead appear to rely more on anterior cingulate cortex (Kennerley et al., 2006; Rudebeck et al., 2008; Luk and Wallis, 2013).

We found a parallel but contrasting role for amygdala responses in learning. Suppression of the amygdala occurred before contingent rewards. The absence of this suppression allowed false learning from free rewards and statistical learning to take place. Counterintuitively, however, subjects with strongest amygdala responses to the free rewards were least likely to learn falsely or statistically from these rewards, perhaps because learning from these rewards also required amygdala suppression. Critically, in experiment 2, we had an entire condition where learning was only possible using statistical learning by spreading credit to the average choice. We found that amygdala became exclusively reward responsive in this condition, but not in the conditions where outcomes could be linked to a particular causative choice. The requirement for amygdala suppression to prevent statistical learning may go some way toward explaining why amygdala lesions during reversal learning lead to faster acquisition of the reversals (Rudebeck and Murray, 2008) and why reversal learning deficits following OFC lesions are abolished after subsequent lesions to the basolateral amygdala (Stalnaker et al., 2007).

Hence, activity in lOFC and amygdala was important for correctly assigning credit for contingent rewards and preventing the misassignment of noncontingent rewards. Such activity might be important as there are other brain systems where learning occurs in simpler fashions, not respecting the true causal structure of the reward environment. We found clear examples of such learning in the putamen and associated motor cortex. Here, rewards evoked stronger responses the sooner they were delivered following a choice, and subjects that exhibited this pattern of activity most strongly were most likely to exhibit noncontingent learning patterns, particularly by learning via proximal choices. Furthermore, we found that responses to contingent rewards in a midbrain region consistent with the location of dopaminergic cell bodies were negatively related to contingent learning. Specifically, midbrain responses to contingent reward were associated with a misattribution of these rewards to both proximal choices (PROX) and the average choice history (SoE_{Ch}), the exact opposite relationship to that observed in lOFC.

A number of neuronal mechanisms have been suggested to underlie credit assignment via contingent and noncontingent learning. Neurons in OFC carry representations of outcome identity over delay periods (Lara et al., 2009), and they encode the choice made by an animal at the time of outcome delivery (Tsujimoto et al., 2009). This might be a mechanism to link outcomes to their causal choices. Alternatively, neurons in primate dorsolateral prefrontal cortex (dlPFC) carry representations of both the current choice and previous choices (Seo et al., 2007). This might be used by reinforcement learning mechanisms in the basal ganglia to bridge temporal gaps when outcomes are delayed. Noncontingent learning mechanisms likely recruit different mechanisms, of which those underlying PROX are arguably best understood. It has been shown that a dopamine burst will only promote spike-timing-dependent plasticity at striatal dendritic spines if that burst occurs within a narrow time window of 0.3–2 s after the sensorimotor input (Yagishita et al., 2014), which is remarkably consistent with the time window during which PROX occurred in our data. Learning via such eligibility traces (Sutton and Barto, 1998) might also be leveraged for learning using SoE_{Ch} when the broad history of choices is reinforced, rather than a single action (Bogacz et al., 2007). Again, coding of past choices by dlPFC neurons might play a role in such eligibility traces spanning multiple actions.

Taken together, we have shown that in a complex environment, behavior is guided by separable contingent and noncontingent learning mechanisms that compete for control over behavior. The lOFC takes a key position in guiding the balance between these mechanisms. It supports contingent learning by encoding contingent associations between outcomes and their causal choices and suppresses the contribution of noncontingent mechanisms. Amygdala activity following a choice plays a role in noncontingent learning via statistical mechanisms, whereas noncontingent learning via heuristic mechanisms is related to reward responses in motor corticostriatal circuitry and regions of the dopaminergic midbrain.

EXPERIMENTAL PROCEDURES

Ethical approval for methods and procedures was obtained from the Central University Research Ethics Committee of the University of Oxford.

Behavioral Analyses Experiment 1

In order to estimate the contribution of different learning mechanisms to behavior, we used a multiple logistic regression that tested the impact of past rewards on future selections of an option, depending on when these rewards occurred relative to choice. We set up the following model:

$$Y = \beta_0 X_0 + \beta_a X_a + \beta_b X_b + \beta_c X_c + \eta,$$

where Y is the dependent outcome "choice of current option" (0/1); X_0 is a constant term; and X_a, X_b, and X_c represent three matrices that each contained 40 regressors (8 × 5) coding for eight past rewards, each split into five bins. Each regressor represented choice of an option in the corresponding time bin (0–0.5, 0.5–1.5, 1.5–2.5, 2.5–3.5, and 3.5–4.5 s prior to reward onset. Matrix X_a represented choices of the "same" option A, whereas X_b represented choices of "different" options B or C. The shape on the current trial was always designated as A, whereas the other shapes were labeled B and C. Matrix X_c was identical to X_b but was interacted with the frequency of previous choices of option A during the past 30 shape presentations. This allowed us to assess how credit for a reward following one choice, B or C, was more likely to be misassigned to A as a function of how often A had been selected in the past. The

nuisance term η represents three further regressors coding for the frequency of previous A choices, the number of overall choices, and the overall number of rewards observed during the past 30 symbol presentations. These nuisance regressors therefore controlled for simple autocorrelation in choice (1) specific to the particular option and (2) generally regardless of what choice was made and additionally for the effects of the number of rewards earned in the recent past. For subsequent analyses (Figures 2A–2C), we summed the resulting regression coefficients over the eight past rewards for each of the five time bins in X_a, X_b, and X_c.

A separate logistic regression was performed to estimate the effect of the average rate of noncontingent rewards on the average rate of responding, termed SoE_{Rew} in the manuscript (Supplemental Experimental Procedures).

Acquisition and Analysis of fMRI Data

MRI data were acquired on a 3T Siemens Verio (experiment 1) and on a 3T Siemens Trio (experiment 2, Siemens Germany) system equipped with a 32-channel phased-array head coil as described in detail previously (Jocham et al., 2012). A total of 514 (experiment 1) or 933 (experiment 2) volumes was acquired on average, depending on subjects' reaction times, thus resulting in total task durations of about 26 and 44 min, respectively. We used Presentation (Neurobehavioral Systems) to present the task and record subjects' behavior.

Analysis of fMRI data was performed using tools from the Functional Magnetic Resonance Imaging of the Brain (FMRIB) Software Library (FSL; Smith et al., 2004). Functional data were motion corrected using rigid-body registration to the central volume (Jenkinson et al., 2002), corrected for geometric distortions using the field maps and an n-dimensional phase-unwrapping algorithm (Jenkinson, 2003), and high-pass filtered using a Gaussian-weighted lines filter (1/100 Hz and 1/50 Hz for experiments 1 and 2), and spatial smoothing was applied using a Gaussian filter with 6 (experiment 1) and 5 (experiment 2) mm full width at half maximum. EPI images were registered with the high-resolution brain images and normalized into standard (MNI) space using affine registration (Jenkinson and Smith, 2001). A general linear model was fitted into prewhitened data space to account for local autocorrelations (Woolrich et al., 2001).

For experiment 1, we set up a single GLM that contained two regressors that coded for the onsets of contingent and noncontingent rewards, respectively. Another regressor contained the onsets of all rewards, but with the time elapsed since last action as a parametric modulator. The duration was modeled with 0.4 s, corresponding to the actual reward display. Two further regressors were included to model the main effect of stimulus presentation (duration 1.5 s) and response (modeled as stick function). In addition, the six motion parameters from the motion correction were included in the model to account for residual head motion. For experiment 2, we constructed a GLM that contained eight separate regressors that accounted for the four triplets of interest (AAA, AAB, BAB, and BAA), split up by the outcome (reward or nonreward) on the second trial, each aligned to the outcome of the triplet's second trial. Contrast images from the first level were then taken to the group level using a random effects analysis. Results are reported at $p < 0.01$, cluster-based correction for multiple comparisons using a cluster-extent threshold of $p < 0.05$, unless stated otherwise.

SUPPLEMENTAL INFORMATION

Supplemental Information includes Supplemental Experimental Procedures, six figures, and one table and can be found with this article online at http://dx.doi.org/10.1016/j.neuron.2016.02.018.

AUTHOR CONTRIBUTIONS

G.J. designed experiments, acquired data, analyzed data, and wrote the manuscript. K.H.B. designed experiments, acquired data, analyzed data, and wrote the manuscript. A.O.C. acquired data, analyzed data, and wrote the manuscript. M.C.K. and A.M.I. designed experiments and analyzed data. M.E.W. designed experiments and wrote the manuscript. M.F.S.R. designed experiments and wrote the manuscript. T.E.J.B. designed experiments, analyzed data, and wrote the manuscript.

ACKNOWLEDGMENTS

This work was supported by a grant from the Federal State of Saxony Anhalt to G.J., project: Center for Behavioral Brain Sciences; a Wellcome Trust 4-year PhD studentship (099715/Z/12/Z) to A.O.C. and a Wellcome Trust Research Career Development Fellowship (WT088312AIA); a Wellcome Trust Senior Research Fellowship (WT104765MA) and a James S. McDonnel Foundation award (JSMF220020372) to T.E.J.B.; and a Wellcome Trust Research Career Development Fellowship (WT090051MA) to M.E.W.

Received: May 21, 2015
Revised: October 19, 2015
Accepted: January 19, 2016
Published: March 10, 2016

REFERENCES

Bogacz, R., McClure, S.M., Li, J., Cohen, J.D., and Montague, P.R. (2007). Short-term memory traces for action bias in human reinforcement learning. Brain Res. *1153*, 111–121.

Charnov, E.L. (1976). Optimal foraging, the marginal value theorem. Theor. Popul. Biol. *9*, 129–136.

Chudasama, Y., and Robbins, T.W. (2003). Dissociable contributions of the orbitofrontal and infralimbic cortex to pavlovian autoshaping and discrimination reversal learning: further evidence for the functional heterogeneity of the rodent frontal cortex. J. Neurosci. *23*, 8771–8780.

Daw, N.D., Niv, Y., and Dayan, P. (2005). Uncertainty-based competition between prefrontal and dorsolateral striatal systems for behavioral control. Nat. Neurosci. *8*, 1704–1711.

Daw, N.D., Gershman, S.J., Seymour, B., Dayan, P., and Dolan, R.J. (2011). Model-based influences on humans' choices and striatal prediction errors. Neuron *69*, 1204–1215.

Dayan, P., and Daw, N.D. (2008). Decision theory, reinforcement learning, and the brain. Cogn. Affect. Behav. Neurosci. *8*, 429–453.

Dayan, P., and Niv, Y. (2008). Reinforcement learning: the good, the bad and the ugly. Curr. Opin. Neurobiol. *18*, 185–196.

Devenport, L.D. (1979). Superstitious bar pressing in hippocampal and septal rats. Science *205*, 721–723.

Dias, R., Robbins, T.W., and Roberts, A.C. (1996). Dissociation in prefrontal cortex of affective and attentional shifts. Nature *380*, 69–72.

Dias, R., Robbins, T.W., and Roberts, A.C. (1997). Dissociable forms of inhibitory control within prefrontal cortex with an analog of the Wisconsin Card Sort Test: restriction to novel situations and independence from "on-line" processing. J. Neurosci. *17*, 9285–9297.

Dickinson, A., Watt, A., and Griffiths, W.J.H. (1992). Free-operant acquisition with delayed reinforcement. Q. J. Exp. Psychol. *45*, 241–258.

Doll, B.B., Hutchison, K.E., and Frank, M.J. (2011). Dopaminergic genes predict individual differences in susceptibility to confirmation bias. J. Neurosci. *31*, 6188–6198.

Fellows, L.K. (2007). The role of orbitofrontal cortex in decision making: a component process account. Ann. N Y Acad. Sci. *1121*, 421–430.

Izquierdo, A., Suda, R.K., and Murray, E.A. (2004). Bilateral orbital prefrontal cortex lesions in rhesus monkeys disrupt choices guided by both reward value and reward contingency. J. Neurosci. *24*, 7540–7548.

Jenkinson, M. (2003). Fast, automated, N-dimensional phase-unwrapping algorithm. Magn. Reson. Med. *49*, 193–197.

Jenkinson, M., and Smith, S. (2001). A global optimisation method for robust affine registration of brain images. Med. Image Anal. *5*, 143–156.

Jenkinson, M., Bannister, P., Brady, M., and Smith, S. (2002). Improved optimization for the robust and accurate linear registration and motion correction of brain images. Neuroimage *17*, 825–841.

Jocham, G., Hunt, L.T., Near, J., and Behrens, T.E. (2012). A mechanism for value-guided choice based on the excitation-inhibition balance in prefrontal cortex. Nat. Neurosci. *15*, 960–961.

Jones, B., and Mishkin, M. (1972). Limbic lesions and the problem of stimulus–reinforcement associations. Exp. Neurol. *36*, 362–377.

Kamin, L.J. (1961). Trace conditioning of the conditioned emotional response. J. Comp. Physiol. Psychol. *54*, 149–153.

Kennerley, S.W., Walton, M.E., Behrens, T.E., Buckley, M.J., and Rushworth, M.F. (2006). Optimal decision making and the anterior cingulate cortex. Nat. Neurosci. *9*, 940–947.

Kringelbach, M.L., and Rolls, E.T. (2004). The functional neuroanatomy of the human orbitofrontal cortex: evidence from neuroimaging and neuropsychology. Prog. Neurobiol. *72*, 341–372.

Lara, A.H., Kennerley, S.W., and Wallis, J.D. (2009). Encoding of gustatory working memory by orbitofrontal neurons. J. Neurosci. *29*, 765–774.

Luk, C.H., and Wallis, J.D. (2013). Choice coding in frontal cortex during stimulus-guided or action-guided decision-making. J. Neurosci. *33*, 1864–1871.

Mishkin, M. (1964). Perseveration of central sets after frontal lesions in monkeys. In The Frontal Granular Cortex and Behavior, J.M. Warren and K. Akert, eds. (McGraw-Hill), pp. 219–241.

Morrison, S.E., and Salzman, C.D. (2009). The convergence of information about rewarding and aversive stimuli in single neurons. J. Neurosci. *29*, 11471–11483.

Padoa-Schioppa, C., and Assad, J.A. (2008). The representation of economic value in the orbitofrontal cortex is invariant for changes of menu. Nat. Neurosci. *11*, 95–102.

Redgrave, P., and Gurney, K. (2006). The short-latency dopamine signal: a role in discovering novel actions? Nat. Rev. Neurosci. *7*, 967–975.

Rudebeck, P.H., and Murray, E.A. (2008). Amygdala and orbitofrontal cortex lesions differentially influence choices during object reversal learning. J. Neurosci. *28*, 8338–8343.

Rudebeck, P.H., Behrens, T.E., Kennerley, S.W., Baxter, M.G., Buckley, M.J., Walton, M.E., and Rushworth, M.F. (2008). Frontal cortex subregions play distinct roles in choices between actions and stimuli. J. Neurosci. *28*, 13775–13785.

Schoenbaum, G., Chiba, A.A., and Gallagher, M. (1998). Orbitofrontal cortex and basolateral amygdala encode expected outcomes during learning. Nat. Neurosci. *1*, 155–159.

Schoenbaum, G., Roesch, M.R., Stalnaker, T.A., and Takahashi, Y.K. (2009). A new perspective on the role of the orbitofrontal cortex in adaptive behaviour. Nat. Rev. Neurosci. *10*, 885–892.

Selemon, L.D., and Goldman-Rakic, P.S. (1985). Longitudinal topography and interdigitation of corticostriatal projections in the rhesus monkey. J. Neurosci. *5*, 776–794.

Seo, H., Barraclough, D.J., and Lee, D. (2007). Dynamic signals related to choices and outcomes in the dorsolateral prefrontal cortex. Cereb. Cortex *17* (*Suppl 1*), i110–i117.

Skinner, B.F. (1948). Superstition in the pigeon. J. Exp. Psychol. *38*, 168–172.

Smith, S.M., Jenkinson, M., Woolrich, M.W., Beckmann, C.F., Behrens, T.E., Johansen-Berg, H., Bannister, P.R., De Luca, M., Drobnjak, I., Flitney, D.E., et al. (2004). Advances in functional and structural MR image analysis and implementation as FSL. Neuroimage *23* (*Suppl 1*), S208–S219.

Stalnaker, T.A., Franz, T.M., Singh, T., and Schoenbaum, G. (2007). Basolateral amygdala lesions abolish orbitofrontal-dependent reversal impairments. Neuron *54*, 51–58.

Sutton, R.S., and Barto, A.G. (1998). Reinforcement learning: an introduction (MIT Press).

Takahashi, Y.K., Roesch, M.R., Wilson, R.C., Toreson, K., O'Donnell, P., Niv, Y., and Schoenbaum, G. (2011). Expectancy-related changes in firing of dopamine neurons depend on orbitofrontal cortex. Nat. Neurosci. *14*, 1590–1597.

Thorndike, E.L. (1933). A proof of the law of effect. Science *77*, 173–175.

Thorpe, S.J., Rolls, E.T., and Maddison, S. (1983). The orbitofrontal cortex: neuronal activity in the behaving monkey. Exp. Brain Res. *49*, 93–115.

Tremblay, L., and Schultz, W. (1999). Relative reward preference in primate orbitofrontal cortex. Nature *398*, 704–708.

Tsujimoto, S., Genovesio, A., and Wise, S.P. (2009). Monkey orbitofrontal cortex encodes response choices near feedback time. J. Neurosci. *29*, 2569–2574.

Walton, M.E., Behrens, T.E., Buckley, M.J., Rudebeck, P.H., and Rushworth, M.F. (2010). Separable learning systems in the macaque brain and the role of orbitofrontal cortex in contingent learning. Neuron *65*, 927–939.

Wilson, R.C., Takahashi, Y.K., Schoenbaum, G., and Niv, Y. (2014). Orbitofrontal cortex as a cognitive map of task space. Neuron *81*, 267–279.

Woolrich, M.W., Ripley, B.D., Brady, M., and Smith, S.M. (2001). Temporal autocorrelation in univariate linear modeling of FMRI data. Neuroimage *14*, 1370–1386.

Yagishita, S., Hayashi-Takagi, A., Ellis-Davies, G.C., Urakubo, H., Ishii, S., and Kasai, H. (2014). A critical time window for dopamine actions on the structural plasticity of dendritic spines. Science *345*, 1616–1620.

Yin, H.H., and Knowlton, B.J. (2006). The role of the basal ganglia in habit formation. Nat. Rev. Neurosci. *7*, 464–476.

Neuron, Volume *90*

Supplemental Information

Reward-Guided Learning with

and without Causal Attribution

Gerhard Jocham, Kay H. Brodersen, Alexandra O. Constantinescu, Martin C. Kahn, Angela M. Ianni, Mark E. Walton, Matthew F.S. Rushworth, and Timothy E.J. Behrens

SUPPLEMENTAL DATA

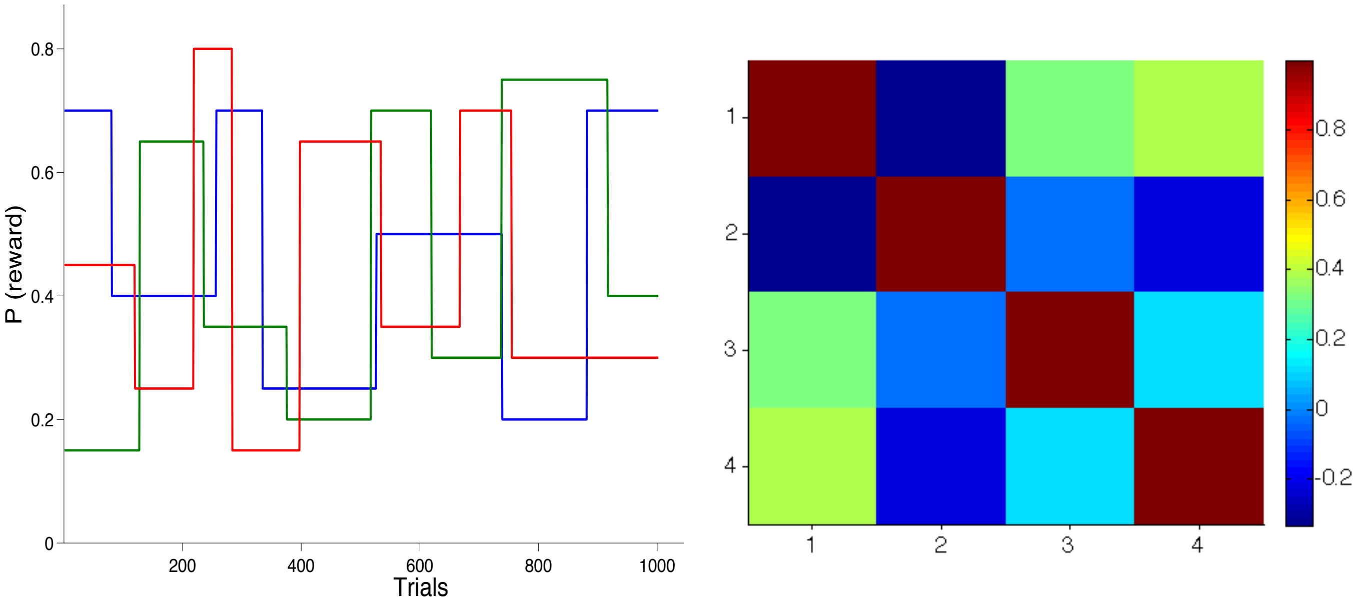

Figure S1 (related to main figure 2). Left: Underlying reward probabilities for the three options in experiment 1. Right: correlation matrix for the behavioural effects in experiment 1 (1 = contingent learning, 2 = PROX, 3 = SoE_{Ch}, 4 = SoE_{Rew}). Subjects responded to 467 ± 21.8 (mean ±SEM) of the 1002 shape presentations and earned 237 ± 9 contingent rewards and 240 ± 6 non-contingent rewards. The number of contingent rewards earned did not differ from the number of non-contingent rewards ($p = 0.81$). Subjects allocated their choices primarily to the best option, where "best" option refers to the option with the highest reward probability of the underlying reward schedule. Even under this conservative performance criterion (the underlying probabilistic reward structure is unknown to participants and due to the frequent reversals several trials are required following each change to obtain a reliable estimate of the options' values) subjects allocated 49% (± 1.48) of their choices to the currently best option, 28% (± 0.61) to the second best, and the remaining 23% (± 1.07) to the third best option. Thus, while far from ceiling, subjects clearly were able to perform the task: selection of the best option was higher than chance (33%) level ($t_{29} = 10.64$, $p < 0.0000000001$) and subjects allocated a higher percentage of their choices to the best compared to the second best option ($t_{29} = 10.22$, $p < 0.0000000001$) and in turn a higher percentage to the second compared to the third best option($t_{29} = 5.96$, $p < 0.00001$).

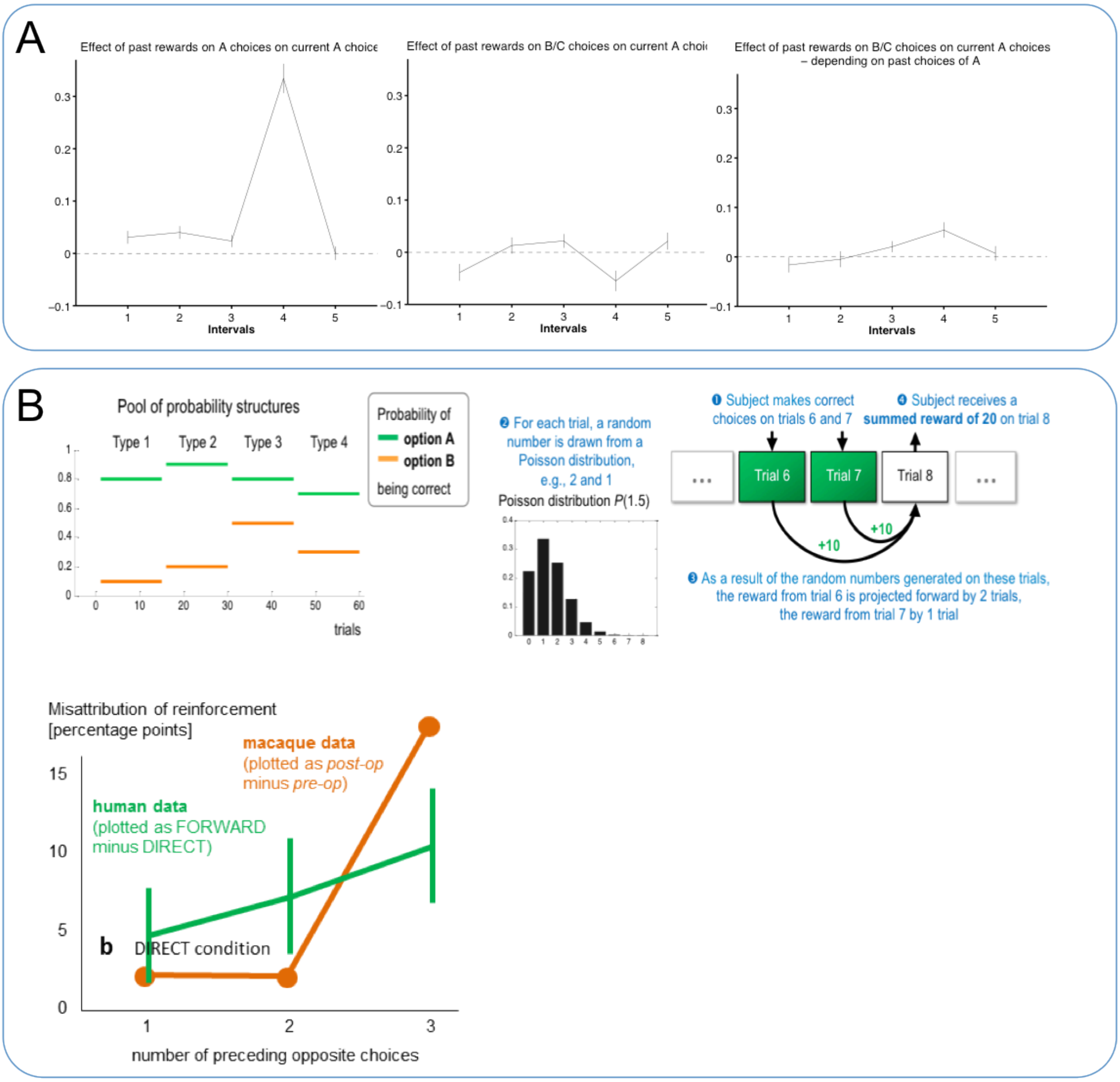

Figure S2 (related to main figure 2). A) Same analysis as reported in main figure 2 A–C replicated without the seven pilot subjects (Considering only the subjects that performed the fMRI experiment, n = 23). Again, rewards depended on the time bin in which they fell (ANOVA, effect of bin, $F_{4,88} = 71.6$, $p < 0.0001$). Rewards had a particularly strong influence on the later selection of the choice made 3 seconds prior to the reward ($t_{22} = 11.94$ $p < 0.0001$, bin 4). Likewise, rewards that occurred in the early bins 1 and 2 ($t_{22} = 2.12$ and $t_{22} = 3.38$, $p < 0.02$) and even in the intermediate bin 3 ($t_{22} = 2.12$, $p = 0.046$) increased the likelihood of repeating these same choices. Rewards in late bin 5 had no effect on behaviour ($p > 0.98$). Furthermore, the effects in the early bins 1 ($t_{22} = 2.05$, $p = 0.026$, one-tailed) and bin 2 ($t_{22} = 2.35$, $p = 0.014$, one-tailed) were bigger compared to late bin 5. Contingent rewards following *B or C* choices increased future *A* choices as an increasing function of the frequency of A choices in the past 30 trials (ANOVA effect of bin $F_{4,88} = 3.29$, $p = 0.0146$, t-test for bin 4: $t_{22} = 3.58$, $p = 0.0017$). The separate regression testing the effects of the time-averaged rate of contingent and noc-contingent rewards on the time-averaged rate of responding showed that the rate of responding strongly depended on the rate of non-contingent rewards ($t_{22} = 5.28$, $p < 0.00005$). The number of contingent rewards obtained (247 ± 10) did not differ from the number of non-contingent rewards (226 ± 4.33, $p > 0.14$). Subjects allocated 48% (± 1.64) of their choices to the currently best option, 28% (± 0.73) to the second best, and the remaining 24% (± 1.16) to the third best option. Selection of the best option was higher than chance (33%) level ($t_{22} = 9.15$, $p < 0.00000001$) and subjects allocated a higher percentage of their choices to the best compared to the second best option ($t_{22} = 8.68$, $p < 0.0000001$) and in turn a higher percentage to the second compared to the third best option($t_{29} = 4.7$, $p < 0.0002$).

B) Top: Block design, probability structure and forward projection of rewards in experiment 2. Bottom: Learning strategies under DIRECT and FORWARD instructions. We used the measure described in the section “validation of instruction manipulation” to delineate the two hypothesized ways of forming associations. When adopting normal contingency learning (as intended in DIRECT blocks), subjects should associate any credit or blame they received after the B choice with B itself; this means that the above measure would be negative. By contrast, when adopting temporal contingency learning (as intended in FORWARD blocks), subjects should associate any credit or blame after their B choice with the history of previous choices, that is, with A; the above measure would be positive. Particularly strong evidence for temporal association learning would be provided by a positive correlation between the length of the history of A choices and the above measure. It would be strongly suggested that a subject forms indirect associations if it was observed that their likelihood of returning to A (staying away from A) increased with a growing number of A choices before a single rewarded (unrewarded) B choice. This is exactly what we observed (bottom). All error bars represent SEM.

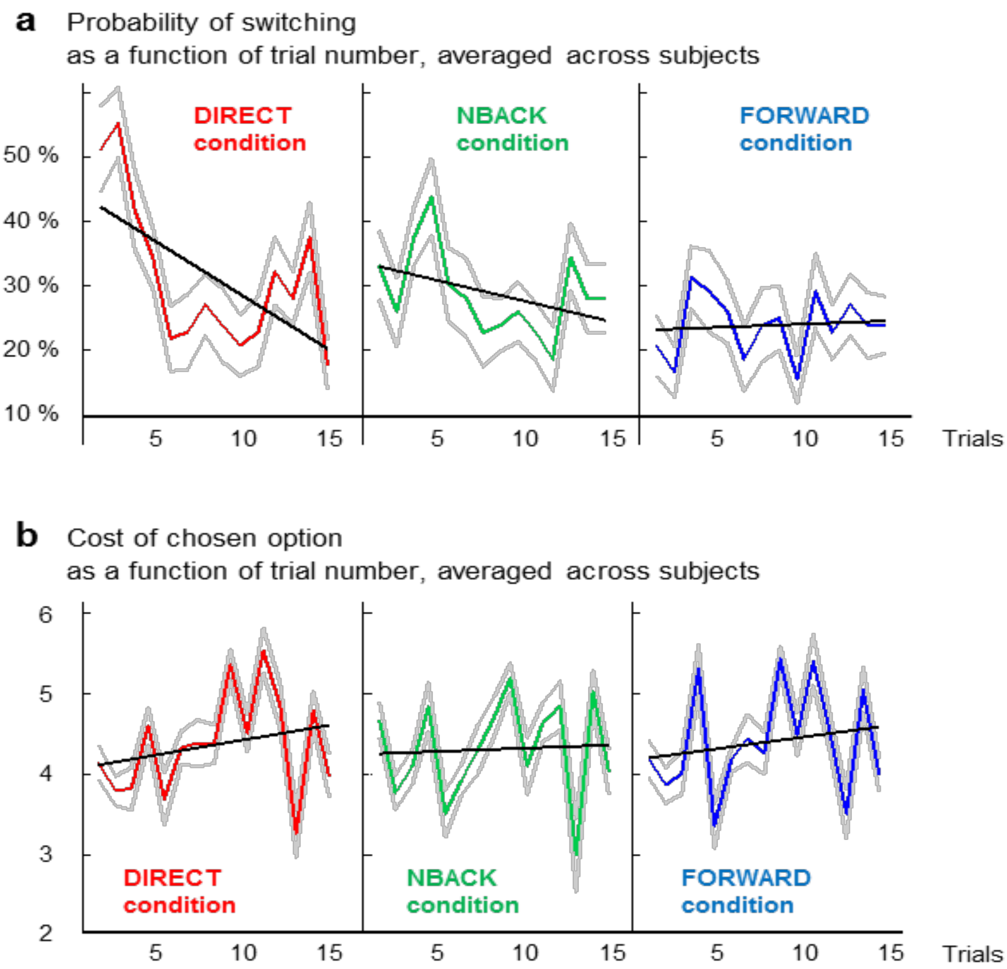

Figure S3 (related to main figure 2). Validation of switching behaviour and costs. Subjects were expected to adopt different policies in different blocks. We tested three hypotheses about how likely subjects should be to switch options *within* a block of trials. In DIRECT blocks, subjects should initially sample the two options freely, potentially attracted towards low costs. Then, towards the end of a block, they were expected to have learned which option had a higher reward probability, and should consequently switch much less frequently. In FORWARD blocks, in which the outcome of an option could only be judged on a longer temporal scale, subjects were expected to stick with an option for a longer time. Their switching probability should be lower than in DIRECT blocks. In NBACK blocks, finally, subjects were expected to display some intermediate switching behaviour in between DIRECT and FORWARD blocks. To test these hypotheses, we averaged switching likelihoods for each trial within a block (Figure S3A). The figure shows the relative number of blocks in which subjects switched from option A to option B, or vice versa, on a given trial. Values are plotted as mean (coloured) +/– one standard error (grey). Only in the case of DIRECT instructions is the slope of a linear least-squares regression through the data (black) significantly different from zero ($p_D < 0.001$, $p_F = 0.73$, $p_M = 0.12$). The diagrams are based on data obtained during fMRI scanning (n = 24). These data allowed us to confirm all three hypotheses. In particular, DIRECT blocks, but not FORWARD or NBACK blocks, showed a significant decline in switching likelihood over the course of a block. In addition, an intriguing effect was observed in the first few trials. In DIRECT blocks, a strong initial peak was followed by a subsequent fall of the average switching likelihood. This pattern reoccurred precisely, albeit in a slightly weaker form, in NBACK blocks – with a delay of about 2 trials (Figure S3A).

Care was taken in the design of the experiment to control for random variations in costs: the same randomly generated sequence of costs was used exactly once within each type of block. In the fMRI variant, for example, with its total number of twelve blocks, only four different sequences of cost pairs were generated for each subject. Therefore, if the costs of both cards offered on a particular trial happened to be very high, this should directly show up in the averaged chosen cost on that trial, across all instructions (Figure S3B). To test this, the figure shows the cost of the chosen card on each trial, averaged for DIRECT, NBACK, and FORWARD blocks, respectively. Since the same randomly generated sequences of costs were used in all block types, the same pattern of peaks and troughs shows up in all subplots, so that

differences between the blocks are solely due to subtle variations in subjects' decision policies. An additional test was made about the evolution of costs during a block, examining the following hypothesis: the easier it is for subjects to learn, the more quickly should they be prepared to choose expensive options which they believe lead to a reward that justifies the cost. The hypothesis was confirmed: DIRECT blocks were the only blocks in which the slope of a linear least-squares regression was significantly greater than zero ($p < 0.05$, figure S3B).

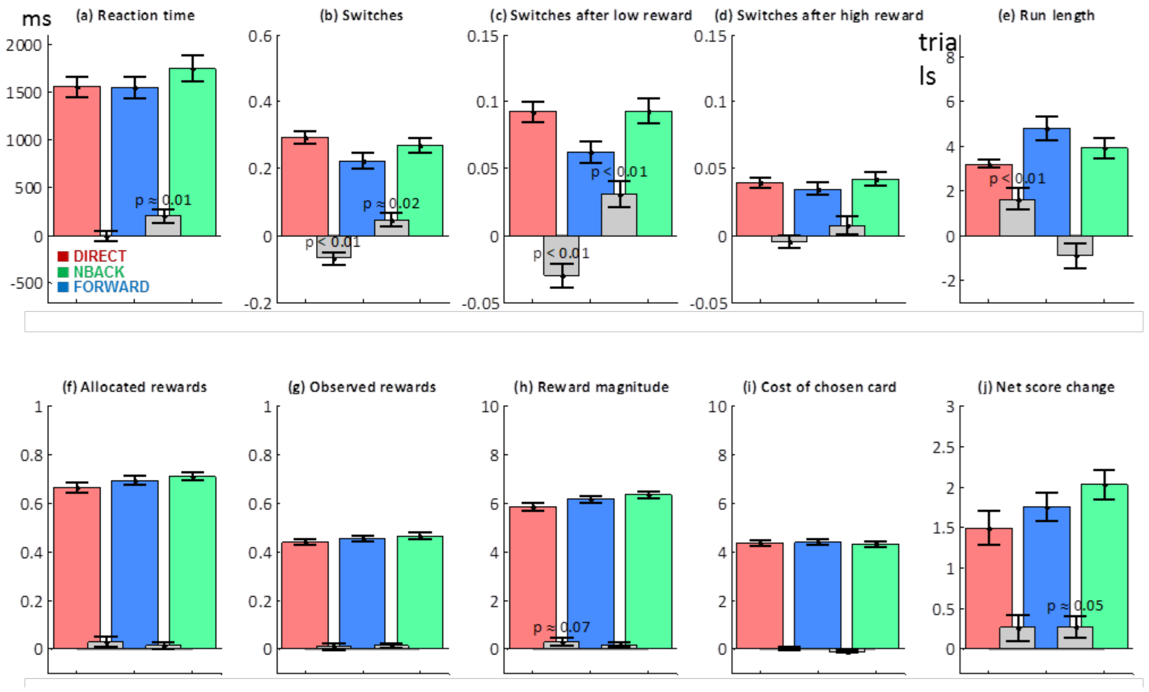

Figure S4 (related to main figure 2). Validation of additional behavioural indices. All experimental blocks were based on the same principle of forward projection of rewards, whereas instructions differed from block to block. As shown above, instructions predicted the form of association learning induced in human subjects. However, in order to allow for meaningful interpretation of any fMRI results, the paradigm had to control for several additional behavioural characteristics that might otherwise introduce confounds in the measured haemodynamic response. Each diagram shows how a particular behavioural descriptor differs between blocks of different instruction types. All values are given as mean +/– one standard error and, unless indicated otherwise on the y-axis, were calculated as `average per-trial' quantities. The three coloured bars represent DIRECT blocks (red), FORWARD blocks (blue), and NBACK blocks (green). The grey bars represent the averaged paired differences between DIRECT and FORWARD (first grey bar) and between FORWARD and NBACK blocks (second grey bar). Wherever a difference was significantly different from zero (with respect to $\alpha=0.1$), the p-value of a two-tailed t-test is given above the respective bar. The diagrams are based on behavioural data obtained during fMRI scanning (n=24).

(a) Reaction times. The average reaction time was calculated as the mean per-trial time between cue and response, that is, between the display of the two options and the subject making a decision. Subjects took longest to respond in NBACK blocks ($p = 0.01$). Increased response times in these blocks could be considered a result of more time required to access working memory and associate the outcome of the previous trial with the choice that led to it. By contrast, no significant difference in reaction times was found between DIRECT and FORWARD blocks, providing a strong control for the fMRI experiment.

(b, e) Switches and run lengths. The average number of switches per trial represents the subjects' mean likelihood of switching from one option to the other on any given trial. It is inversely proportional to the run length, the number of trials for which subjects would, on average, stick with the same option. In order to find out about the reward probabilities of the two options, subjects were expected to sample the same option for longer consecutive periods of time in FORWARD blocks than in DIRECT blocks, in which subjects were expected to choose among the two options in a more volatile way. This is exactly what was found: the average switching likelihood was highest in DIRECT blocks and lowest in FORWARD blocks; equivalently, the mean run length was found to be shortest in DIRECT blocks and longest in FORWARD blocks, differing by approximately two trials. This shows that the paradigm significantly influenced subjects' behaviour but, at the same time, provided a one-way control for the fMRI experiment: should a brain area be found to be more active in FORWARD blocks than in DIRECT blocks, then this finding could not be explained away by a higher frequency of behavioural switches (Crone et al., 2006).

(c, d) Switches after low/high reward. An unexpected result was obtained in the dependency of switches on reward magnitudes. In DIRECT and NBACK blocks, subjects had been instructed only to take into account whether a particular option was rewarded, but not to be distracted by the reward magnitude - an ostensibly 'random' number $r \in \{10, 20, 30,..\}$. However, splitting up trials into those following a low reward ($r = 10$) and those following a high reward ($r > 10$) revealed that subjects, perhaps unconsciously, did take into account reward magnitudes: they were more likely to switch away from their current option after a low reward than after a high reward. This effect is easiest to interpret in DIRECT blocks, in which a switch represents a rejection of the last option.

(f, g, h) Rewards. The experimental paradigm was designed to lead to similar reward levels under DIRECT and FORWARD conditions. Here, this notion was once more confirmed by looking at three more detailed descriptors of reward. In these diagrams, the number of allocated rewards describes how often a rewarded card was chosen, whereas the number of observed rewards describes how often a reward was actually displayed on the screen. This number necessarily had to be smaller than the number of allocated rewards: rewards were allowed to pile up on the same trial, and were sometimes projected forward beyond the end of a block. Reassuringly, no significant differences were found between different instructions in allocated rewards, observed rewards, or (observed) reward magnitudes. This finding provided a strong control for the fMRI experiment: differences in activity between the different instructions would not be explicable in terms of differences in rewards.

(i) Costs. An ever more stringent test of the validity of the experimental paradigm was made by looking at the costs of the chosen cards, which were, unlike rewards, under the subjects' direct control. As before, no significant differences between the blocks were found. In particular, no type of instruction led subjects to ignore reward probabilities and behave in a mere cost-minimizing fashion. Both findings provided strong controls for the fMRI experiment.

(j) Net score changes. The difference, on each trial, between the observed reward and the cost yielded the net score change. Although slightly more frequent observed rewards, slightly higher reward magnitudes, and slightly lower average costs of the chosen card led to a net score change in NBACK blocks that was higher than in both other types of block ($p < 0.05$), no significant difference was found between DIRECT and FORWARD blocks, providing a strong control for fMRI.

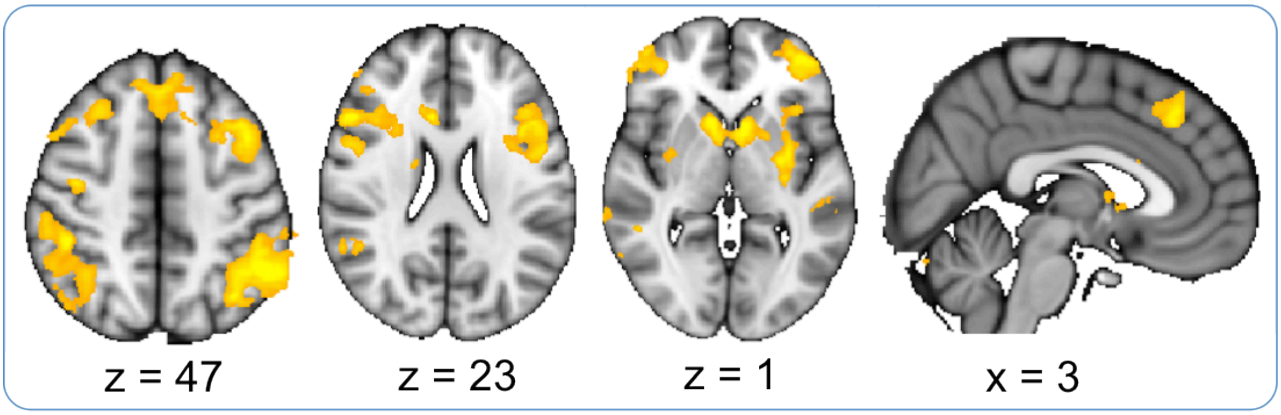

Figure S5 (related to main figure 3). Additional areas found active in the main contrast contingent – non-contingent rewards in experiment 1.

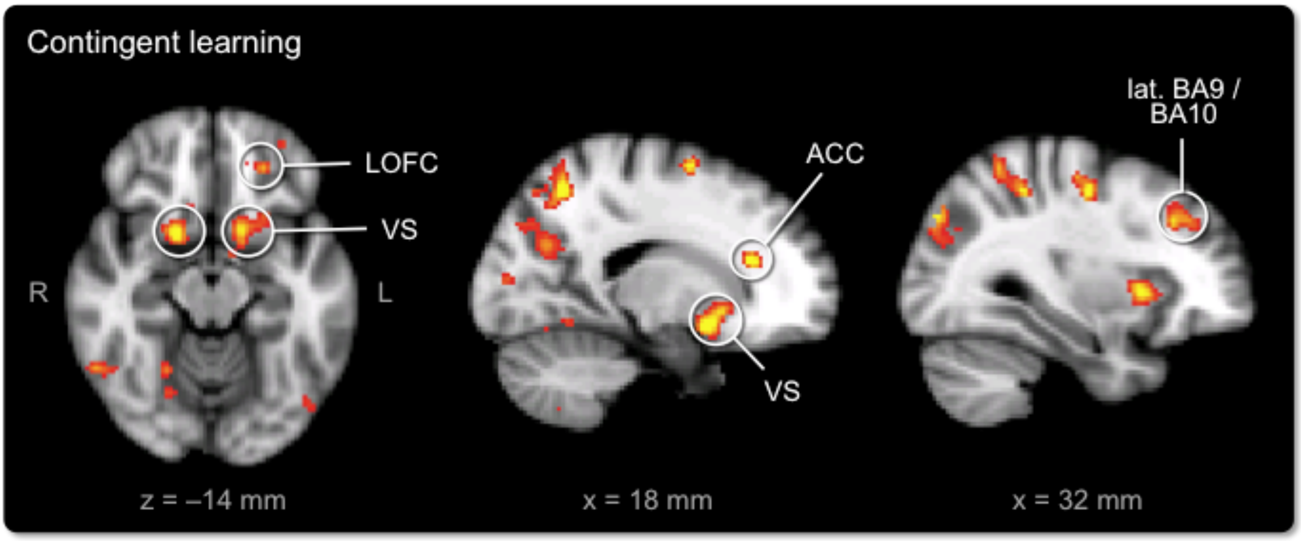

Figure S6 (related to main figure 4). Whole-brain contingency effects (experiment 2). Several areas were found to reflect, at the time of observing the outcome of a decision, whether a correct choice-outcome-update contingency is being applied ($z > 3.1$). Of particular interest in this result are the ventral striatum (VS), lateral orbitofrontal cortex (OFC), anterior cingulate cortex (ACC), and the border between lateral Brodmann areas (BA) 9 and 10.

Table S1 (related to main figure 3). List of peak activation for clusters found in the main contrast [contingent – non-contingent rewards in experiment 1. This table provides a full list of cluster maxima for this contrast. Please note however that despite the conservative thresholding approach ($p < 0.001$ activation threshold, cluster-based threshold at $p < 0.05$), many of the activations found here are rather large and span more than a single brain area. For this reason, the lables we assigned to these clusters should be taken with care. We additionally display several views (figure S5) to give a more comprehensive picture of the pattern of brain activity found here.

Region	MNI coordinates (xyz)	peak Z-score	cluster size (mm3)
Lateral prefrontal cortex	-33 8 20	5.24	54563
Posterior parietal cortex	-51 -51 44	5.4	14510
Posterior parietal cortex	45 -38 49	4.85	8352
Inferior/middle temporal gyrus	-58 -36 -12	4.96	6502
Lateral orbitofrontal cortex	-24 35 -12	5.1	5192
Inferior/middle temporal gyrus	63 -39 -6	5.13	3802
Cerebellum	33 -64 -32	4.77	2360
Posterior superior temporal sulcus	-56 -52 17	4.46	1365
Visual cortex (V2/V3/V4)	-20 -87 -11	5.22	1325
Primary motor cortex	41 -9 48	4.4	1030
Temperoparietal junction	57 -50 23	3.77	811

SUPPLMENTAL EXPERIMENTAL PROCEDURES

Experiment 1

Behavioural task

Subjects observed geometrical shapes (circle, square, triangle) moving across the screen from left to right in random succession. Every shape appeared from underneath a "curtain" (a grey rectangle on the left side) and moved rightwards during a period of 1.5 s until it disappeared under another "curtain" on the right-hand side. Subjects could choose to either select the shape present on the screen or ignore it. A shape could be chosen by pressing its associated button (index, middle, and ring finger) with the right hand. Shapes were associated with a probability of reward, when selected, that was independent between options (shapes) and changed several times during the experiment (Figure S1). If a shape was chosen, a response cost equivalent to 1/3 point was deducted which was visualised to subjects by shrinking of the progress bar at the bottom of the screen. This manipulation was implemented to discourage subjects from responding to every option. If a reward was earned for a choice, the equivalent of 1 point was added to the progress bar. The subjects' aim was to hit the gold target line to the lower right of the screen to win £2, after which the progress bar was reset to zero and subjects started afresh. Importantly, if a subject's choice resulted in a reward, this reward was delayed by a fixed interval of 3 s. This meant that whenever participants observed a reward, they had to link this with the option chosen 3 s before, not with the shape currently onscreen. In addition to these contingent rewards,

there was also a fixed probability p = 0.3 during every symbol presentation that a non-contingent reward could be delivered. If such a non-contingent reward was scheduled, it was present with random timing during the shape presentation. Both types of rewards were presented for 400 ms and consisted of a picture of Scrooge McDuck diving in gold. To make contingent and non-contingent rewards visually distinguishable, they were presented in different colours. For half the participants, contingent rewards were in blue and non-contingent rewards were in red, and the reverse was true for the other half of volunteers. Subjects were explicitly instructed about all these task features. Before they entered the scanner, they first performed a time estimation task where they learnt to estimate a 3 s interval. Next, they learnt the mapping of response fingers to shapes. Following this, they performed a training version of the task that included only the contingent rewards. Thereafter, they performed a version of the task that was identical to the experimental task, including contingent and non-contingent rewards, only with a simpler probabilistic structure. The experimental task consisted of 1002 shape presentations and lasted approximately 25 minutes.

Linear regession testing the effect of average reward rate on average rate of responding

To test whether subjects' average rate of responding depended on the average rate of rewards, we set up the following linear model:

$$Y = \beta_0 X_0 + \beta_1 X_{NCR} + \beta_2 X_{CR}$$

where the dependent variable Y is response on each trial obtained by smoothing with a Gaussian window of 30 trials, X_0 is a constant, and X_{NCR} and X_{CR} represent the rate of non-contingent and contingent rewards, respectively, again obtained by smoothing with a Gaussian window of 30 trials.

Task-related functional connectivity (PPI) analyses between ventral striatum and OFC

Raw BOLD signal timecourses were extracted from the ventromedial striatum (VMS) at the peak coordinate identified in the main contrast of contingent – non-contingent rewards in experiment 1 (see main text). Timecourses were extracted from a maks that contained the peak coordinates in both hemispheres. A general linear model was set up that was identical to that described in the main text for experiment 1, but additionally contained the (demeaned) VMS BOLD timecourse and two PPI regressors that were generated by interacting the contingent and non-contingent reward regressors, respectively, with the VMS timecourse. Differential connectivity during contingent versus non-contingent rewards was then assessed by directly contrasting the two PPI regressors.

Experiment 2

Behavioural task

To test competing hypotheses about the neural mechanisms underlying contingency learning, we designed a novel probabilistic sequential decision-making task. In this task, participants had to learn, by trial and error, the reward probabilities of two options. While undergoing fMRI scanning, each subject completed 12 blocks of our learning task with 15 trials each. In every block, two previously unseen fractals had different probabilities of being associated with a reward; these probabilities varied between blocks. On each trial, participants were asked to choose between two alternative options, represented by two fractal stimulus patterns (Figure 1, right). Unknown to participants, the two

options had different probabilities of leading to a reward. For example, if one of the options had a reward probability of 80%, then that card would, on average, be rewarded on 8 out of 10 trials. The reward probabilities of the two options were mutually independent. Thus, on any given trial, neither card, one card, or both cards could be rewarded. Probabilities varied throughout the experiment, inducing the requirement for continuous learning (Figure S2B).

To encourage subjects to switch options above and beyond normal exploration behaviour (Macready and Wolpert, 1998), the two cards were associated with costs randomly sampled from the interval [1....9]. Subjects began the experiment with an initial baseline score. When choosing a card, its associated cost would be deducted from this score. If the card was rewarded, the score would then be increased by the magnitude of the reward. The overall score subjects had reached by the end of the experiment was directly translated into monetary reimbursement. The task comprised three conditions. In the DIRECT condition, subjects were instructed that, whenever a rewarded option had been chosen, its reward was presented on the same trial. In the NBACK condition, subjects were told that rewards were projected forward by a known number of trials (one or two) such that any given outcome would have to be linked to a previous choice. In the FORWARD condition, finally, subjects were instructed that rewards were delayed by a random number of trials such that outcomes could no longer be linked to their underlying causative decisions (Figure 1, right). In other words, in FORWARD, subjects only know that one of four choices is likely responsible (forward projection of rewards by more than three trials is very unlikely, see supplementary figure S2B). While subjects in FORWARD thus do not know which action caused a particular reward, they can nevertheless learn by assigning credit to the average choice. Unknown to subjects, all three conditions (DIRECT, NBACK, and FORWARD) followed the FORWARD scheme. Thus, across all conditions, rewards were not delivered immediately; instead, they were delayed, or projected forward, by a small random number of trials. This number was, on each trial, drawn from a Poisson distribution P(1.5). The number of trials by which a reward was projected forward could be any non-negative integer, with an average of 1.5 trials. Individual rewards were worth 10 points. If two rewards happened to be projected forward to the same future trial, their values were summed (Figure S2B). This design ensured a delayed and interleaved order of reinforcements, preventing subjects from being able to benefit from forging direct associations (Figure S2B). Since the underlying structure was identical throughout (forward projection and summation of rewards), subjects would inevitably observe varying reward magnitudes in all blocks (10, 20, 30, …). In DIRECT and NBACK blocks, these were explained to them as random multiples of 10 generated on each rewarded trial. Only in FORWARD blocks were subjects accurately told that rewards greater than 10 were a result of the summation of multiple rewards that happened to be projected forward onto the same trial. The fMRI experiment consisted of 12 blocks with varying instructions. Each block contained 15 trials only. Their probabilities followed one out of four predefined probability schemes. Since left/right positions of the two options were randomized within and across subjects, probabilities were decorrelated both from the side of presentation and from the fractal patterns representing the two options.

Validation of instruction manipulation

For the purpose of the behavioural validation of our paradigm, we acquired an initial dataset from 12 healthy volunteers (8 male, 4 female), aged between 22 and 36. This initial validation experiment consisted of one DIRECT block, one NBACK block, and one FORWARD block, each containing 350 trials. The reward probabilities of the two options followed a scheme that was identical for all three blocks. It was designed to contain stretches of fixed, drifting, and reversing probabilities. As shown by this initial behavioural pilot, our instruction manipulation did not only robustly induce the different behaviours that one would expect under the three different instruction types. Critically, this design also provided a number of strong controls for the subsequent imaging analysis. Most importantly, there was no significant difference in reward levels across conditions.

The principal aim of the experimental paradigm was to induce different forms of contingency learning in human subjects. Specifically, FORWARD instructions, compared to DIRECT instructions, were to replicate the behavioural effect observed in OFC-lesioned monkeys . This had led to two specific questions. First, did DIRECT blocks make subjects form associations between choices and their immediate outcomes? Second, did FORWARD blocks lead subjects to associate a reinforcement outcome with the recent history of choices?

In order to answer these questions, a measure for the likelihood of choosing A after a series of A choices and a single B choice was calculated as

$$\frac{\#(\overbrace{\mathrm{A}\cdots\mathrm{A}}^{k}\mathrm{B}^{+}\mathrm{A})}{\#(\underbrace{\mathrm{A}\cdots\mathrm{A}}_{k}\mathrm{B}^{+})} - \frac{\#(\overbrace{\mathrm{A}\cdots\mathrm{A}}^{k}\mathrm{B}^{-}\mathrm{A})}{\#(\underbrace{\mathrm{A}\cdots\mathrm{A}}_{k}\mathrm{B}^{-})} \quad \forall k = 1,2,3,\ldots,$$

where B^+ denotes a rewarded, and B^- an unrewarded, choice of option B. The term $\#(\mathrm{AAB}^{+}\mathrm{A})$, for instance, denotes the number of times the sequence $\mathrm{AAB}^{+}\mathrm{A}$ was found in the entire sequence of choices of a subject. It represents all instances when a subject returned to option A after two consecutive A choices and a single rewarded B choice.

Assessment of how different instructions induce different forms of learning

Despite the true contingencies being identical in each block (FORWARD), we hoped that different instructed contingencies would induce different behaviours. In order to examine whether this was the case, we constructed a simple logistic regression model to explain each subject's choices in terms of their previous choices and rewards. If subjects were indeed learning according to our instructed contingencies, credit for a reward should be assigned: to the immediately preceding choice in the DIRECT condition; to the previous choice in the 1BACK condition; to the choice before the previous choice in the 2BACK condition; and credit should be distributed across the different choices in the FORWARD condition. Hence, in order to explain each choice, we included 4 regressors that indicated the previous 4 choices, and 3 sets of regressors that interacted these choices with the occurrence of rewards: at the current trial; at the following trial; and at the following-but-one trial. These regressors can be arranged into the lower quadrant of a square (Main figure 2E) where the lead diagonal represents DIRECT learning (red), the next lower diagonal represents 1BACK learning (green), and the third diagonal 2BACK learning (yellow).

When estimating this model, we found that a different set of regressors received loading in each instructed condition, despite the true contingencies being identical across all blocks. Specifically, when subjects were instructed that contingencies were DIRECT, the DIRECT regressors were loaded; when 1BACK and 2BACK instructions were given, 1BACK and 2BACK regressors were loaded, respectively; and when subjects were instructed that rewards would be projected FORWARD by a random number of trials, all three sets of regressors were loaded. This statistical interaction between instruction and regressor type was then assessed by a 2-way ANOVA on regressor loadings.

Selection of independent OFC ROI

Our design allowed us to compute contrast from one set of trials and use the peak location to extract data from a different set of trials, thus avoiding any selection bias. More specifically, the data used for the BA-contrasts shown in main figure 4 A, B, C and E are derived from a contrast obtained from using AA trials only. Conversely, the data used for the AA contrasts depicted in main figure 4D and F are derived from the peak coordinate of the contrast obtained from using BA trials only.

SUPPLMENTAL REFERENCES

Crone EA, Wendelken C, Donohue SE, Bunge SA (2006) Neural evidence for dissociable components of task-switching. Cereb Cortex 16:475-486.

Macready WG, Wolpert DH (1998) Bandit Problems and the Exploration/Exploitation Tradeoff. IEEE Transactions on Evolutionary Computations 2:2-22.
